# A Heterogeneous Acid‐Base Organocatalyst For Cascade Deacetalisation‐Knoevenagel Condensations

**DOI:** 10.1002/cssc.202400866

**Published:** 2024-09-03

**Authors:** Ashis Chhetri, Ashakiran Maibam, Subashani Maniam, Ravichandar Babarao, Karen Wilson, Adam F. Lee, Joyee Mitra

**Affiliations:** ^1^ Inorganic Materials & Catalysis Division CSIR-Central Salt & Marine Chemicals Research Institute Bhavnagar, Gujarat 364002 India; ^2^ Academy of Scientific and Innovative Research (AcSIR) Ghaziabad 201002 India; ^3^ School of Science, STEM College RMIT University Melbourne VIC 3000 Australia; ^4^ Physical and Materials Division CSIR-National Chemical Laboratory Pune 411008 India; ^5^ Centre for Advanced Materials and Industrial Chemistry (CAMIC) RMIT University Melbourne, VIC 3001 Australia; ^6^ CSIRO Melbourne VIC 3168 Australia; ^7^ Centre for Catalysis and Clean Energy School of Environment and Science Griffith University Gold Coast QLD 4222 Australia

**Keywords:** Organocatalysis, Chemical cascade, Charge-assisted hydrogen bonding, Antagonistic acid-base sites, Sustainable catalysis

## Abstract

Multifunctional heterogeneous catalysts are an effective strategy to drive chemical cascades, with attendant time, resource and cost efficiencies by eliminating unit operations arising in normal multistep processes. Despite advances in the design of such catalysts, the fabrication of proximate, chemically antagonistic active sites remains a challenge for inorganic materials science. Hydrogen‐bonded organocatalysts offer new opportunities for the molecular level design of multifunctional structures capable of stabilising antagonistic active sites. We report the catalytic application of a charge‐assisted, hydrogen‐bonded crystalline material, bis(melaminium)adipate (**BMA**), synthesised from melamine and adipic acid, which possesses proximate acid‐base sites. **BMA** exhibits high activity for the cascade deacetalisation‐Knoevenagel condensation of dimethyl acetals to form benzylidenemalononitriles under mild conditions in water; **BMA** is amenable to large‐scale manufacture and recycling with minimal deactivation. Computational modelling of the melaminium cation in protonated **BMA** explains the observed catalytic reactivity, and identifies the first demethoxylation step as rate‐limiting, which is in good agreement with time‐dependent ^1^H NMR and kinetic experiments. A broad substrate scope for the cascade transformation of aromatic dimethyl acetals is demonstrated.

## Graphical abstract

Bis(melaminium)adipate, comprising charge‐assisted, hydrogen‐bonded melamine and adipic acid co‐crystals, is an effective acid‐base organocatalyst for the cascade transformation of (aromatic) dimethyl acetals to benzylidenemalononitriles under mild reaction conditions with excellent sustainability metrics.

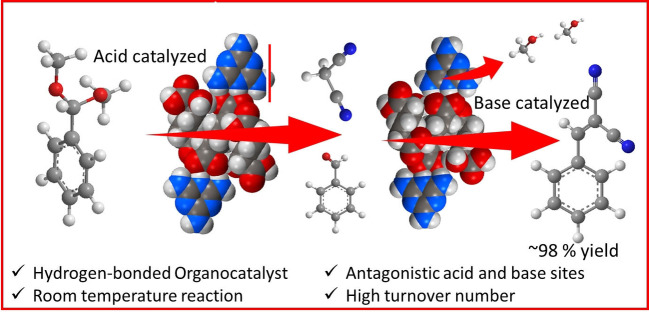

## Introduction

Catalytic transformations involving cascade reactions have garnered interest from green chemistry perspective, as pathways to accomplish multistep organic transformations, which precludes the necessity of costly and time‐consuming isolation/purification of intermediates, minimizing waste generation.^[1]^ Improved atom‐economy, energy‐efficiency and diminished operational costs are the added benefits of cascade reactions.^[4]^ Orthogonal tandem catalysis involves multiple, non‐interfering catalytic sites participating in diverse sequential transformations (a chemical cascade). Rational design of catalysts for tandem heterogeneous catalytic transformations has proven arduous, as the process necessitates antagonistic active sites in close proximity, which operate independently without mutual intrusion.^[5]^ A rigid architecture is necessary in the catalyst to spatially segregate the antagonistic sites. These traits are critical in the catalyst for antagonistic reactions as cross‐reactions are highly possible where one component of the reaction mixture may interfere with the reactivity of the other components. Multistep orthogonal reactions are common in nature, as the enzyme active sites are pre‐organized with precise control over the spatial distribution and compartmentalisation of active sites for sequential reactions giving rise to the desired product with minimal diffusion of the reactants between active sites.^[8]^ Inspired by the compartmentalisation of antagonistic active site practiced by nature, researchers have adapted the approach of incorporating antagonistic functionalities (e. g., acidic and basic sites) in different catalytic fragments to ensure site‐isolation, including star polymers, core‐shell structures, hydrogels, zeolites, silica, graphene oxide‐based systems and so on.[^4^, ^9^] Though these bifunctional acid‐base catalysts have proven effective in incorporating chemically incompatible sites in a single material and in controlling the reaction sequence, they often suffer from low active site density and limited accessibility of the embedded active sites between layers.^[20]^ Subsequently, attempts have been made to reduce the diffusion path by adjusting the distance between the antagonistic catalytic centres by combining the catalytic sites in the same material, in order to diminish the reaction time and improve reaction efficiency.[^4^, ^22^]

Bifunctional mesoporous catalysts containing organic acid/base, or mixed organic base/inorganic acid functions, have shown promise for the tandem deacetalization‐Henry reaction of benzaldehyde dimethylacetal with nitromethane.[^20^, ^24^] However, such amine and sulfonic/phosphotungstic acid functionalised silicas possess acid/base loadings ≤1 mmol.g^−1^ and require temperatures ~50 °C under an inert atmosphere. Core‐shell catalysts, comprising a porous sulfonic acid silica shell encapsulating an amine silica core, are reported for the same deacetalization‐Henry reaction at even higher temperature (90 °C) and with slower kinetics, possibly due to rate‐limiting diffusion to and from the core.^[25]^ Synthesis of antagonistic acid‐base catalysts is also cumbersome, requiring multistep protocols and often structure‐directing agents, or protection‐deprotection strategies,[^20^, ^24^, ^28^] that produce significant waste and are inconsistent with green chemistry principles.

Microporous organic polymers functionalised with either sulfonic acid or benzylamine have been demonstrated for cascade deacetalization‐Henry and deacetalization‐Knoevenagel reactions at 80 °C, but as physical mixtures of discrete acid and base polymer catalyst particles.^[30]^ Composites of metal‐organic frameworks (MOFs) and covalent organic frameworks (COFs) have also been exploited for the deacetalization‐Knoevenagel condensation cascade of benzaldehyde dimethylacetal with malononitrile, although the optimal PCN‐222‐Co@TpPa‐1 (MOF@COF) catalyst was still sluggish at 50 °C.^[34]^ Furthermore, MOFs are notoriously unstable in acids, while the stability of mesoporous silica is limited to acidic pH and organic media.^[36]^ Carboxyl groups at the edges of graphene oxide are reported to act in concert with amine groups introduced to the basal planes post‐synthesis for the deacetalization‐Knoevenagel condensation of benzaldehyde dimethyl acetal with ethyl cyanoacetate, but required a toluene solvent at 80 °C.^[16]^ A porous polymer containing immobilized imidazolium cations and hydroxyl anions (forming Lewis acid‐base pairs) was recently reported for the deacetalization‐Knoevenagel condensation of benzaldehyde dimethylacetal and malononitrile at 60 °C in water under air,^[37]^ however acid/base loadings were not quantified hence absolute performance cannot be benchmarked. In light of the above, there is a significant need for low cost, one‐pot synthetic routes to antagonistic acid‐base heterogeneous catalysts offering high activity and stability in green solvents such as water for chemical cascades.

Hydrogen‐bonded organic systems have recently emerged as a fascinating class of easily synthesizable crystalline materials with tunable chemical compositions that are fabricated by the assembly of organic building blocks by hydrogen‐bonding interactions.^[30]^ Reversible nature of hydrogen bonds results in easy solution processibility and regeneration of these materials, which enable them to serve as perfect heterogeneous catalysts.^[34]^ Hence, the utility of these systems span storage, separation of gas mixtures and chemicals, in energy, catalytic applications as supports and so on.[^30^, ^33^, ^36^] The advantages of these hydrogen‐bonded systems from the perspective of heterogeneous catalysis involves the diverse incorporation of functional groups through precursor‐engineering, high chemical stability, insolubility in common organic solvents, interaction and pre‐activation of substrates and their self‐repairing ability due to the reversible nature of hydrogen bonds.^[37]^ Though substantial progress has been achieved in hydrogen‐bonded organic systems, their development still lags behind those of MOFs and COFs/COPs. Though it is known that the activation of substrates is improved with the incorporation of multiple hydrogen bond donors,^[40]^ but the utility of hydrogen‐bonded systems have mostly been restricted to use as porous supports for heterogeneous catalysts, and rarely as catalysts themselves.[^36^, ^41^] Considering the stability of organocatalysts in air and water,[^40^, ^42^] separation of antagonistic catalytic sites ascertained by hydrogen‐bonded systems while keeping them in close proximity, facile cost effective synthesis and convenient tailoring of functionality, we envisage hydrogen‐bonded organic materials as an excellent choice for heterogeneous catalyst for antagonistic reactions, with enhanced performance and recyclability. However, hydrogen‐bonded organic systems have not been used as bifunctional catalysts for chemical cascades to the best of our understanding.

In the present work, we have employed inexpensive melamine and adipic acid precursors to design a charge‐assisted hydrogen‐bonded system (**BMA**) via a straightforward hydrothermal strategy,^[43]^ as an efficient heterogeneous acid‐base bifunctional catalyst for the execution of a one‐pot cascade deacetalisation‐Knoevenagel condensation in water and under mild conditions. Adipic acid was selected due to its: (i) low cost and ready availability (as a commodity chemical for the synthesis of Nylon‐66); (ii) relatively low toxicity as compared to e. g., terephthalic acid; (iii) high melting point (152 °C) as an unbranched dicarboxylic acid with an even number of carbon atoms (compared to odd numbered homologues e. g., glutaric acid 95–98 °C or pimelic acid 103–105 °C) and hence expected higher thermal stability while providing sufficient structural rigidity to prevent intramolecular condensation; (iv) buffering ability in acidic pH (adipates are used as acidity regulators) to act as a mild acid alongside basic melamine; and (v) solubility in water which facilitates purification by washing. We anticipated that **BMA**, with both acidic and basic sites would be effective as a heterogeneous bifunctional catalyst, as the first deacetalisation step requires an acid catalyst whereas the second condensation step requires a base catalyst. Though melamine‐containing systems have been reported for Knoevenagel condensation, utilising their basicity, such systems have not been explored for catalytic cascades.^[44]^ The deacetalisation‐Knoevenagel condensation cascade is useful to synthesise intermediates for cosmetics, perfumes and pharmaceuticals.^[46]^ **BMA** is shown as an effective and atom‐efficient catalyst, with mechanistic insight from quantum mechanical calculations supporting deacetalisation at a protonated triazine ring NH^+^ followed by condensation at an exocyclic amine.

## Experimental Section

### Synthesis of BMA

Melamine (0.001 mol, 0.1261 g) and adipic acid (0.003 mol, 0.4384 g) were thoroughly mixed by grinding in an agate mortar pestle. The resulting solid mixture was transferred to a Teflon bomb to which 15 mL of deionised water was added. The autoclave was then placed in an oven and heated at 160 °C for 72 h. After cooling, colourless block‐shaped crystals were obtained by gravity filtration, thoroughly washed with deionised water and air dried overnight. The isolated yield of the crystals was ~85 % on a molar basis.

### Representative Procedure for Catalytic Cascade Reaction

1 mmol of benzaldehyde dimethyl acetal and 1.02 mmol of malononitrile were added to a glass vial containing 3 mL of DI water and the solution was stirred at room temperature for 5 min under air. Subsequently, 10 mg of **BMA** (0.0207 mmol) were added to the mixture stirring continued for 1 h. The initially colourless solution turned pale yellow, possibly due to formation of the benzaldehyde intermediate before producing an off‐white precipitate. The precipitate was collected, extracted in CHCl_3_, and dried over anhydrous Na_2_SO_4_ to produce an off‐white powder. The final product yield was calculated by ^1^H NMR (98 %) and was in good agreement with the isolated yield.

## Results and Discussion

### Synthesis and Characterization of BMA

**Figure 1:**
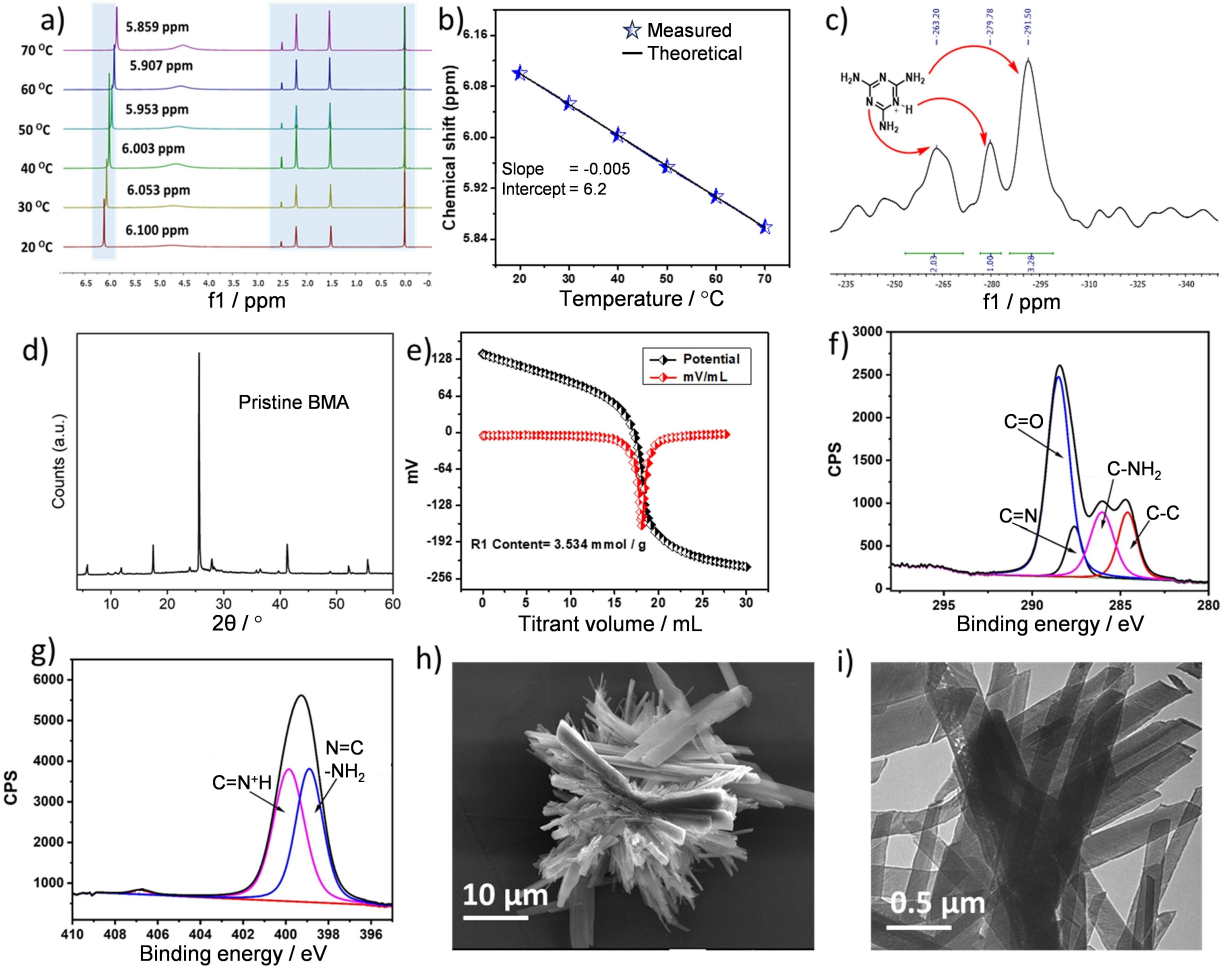
Temperature‐dependent (a) ^1^H NMR spectra and (b) associated chemical shifts of ‐NH_2_ protons in **BMA**, (c) solid‐state ^15^N CP‐MAS NMR spectrum of **BMA**, (d) powder XRD diffractogram of **BMA**, (e) potentiometric titration of **BMA** with NaOH, (f) C 1s and (g) N 1s XP spectra of **BMA**, and corresponding (h) FE‐SEM and (i) TEM images of **BMA** nanocrystals.

Bis(melaminium)adipate (**BMA**) crystals were prepared by the hydrothermal reaction of melamine and adipic acid at 160 °C, according to our previously reported protocol (Scheme S1).^[43]^ The resulting colourless crystalline solid was insoluble in water and common organic solvents, which single crystal XRD analysis identified as comprising one adipate dianion, two melaminium cations, one adipic acid and two water molecules (Figure 1a‐b). Adipic acid acts as an interlayer mediator between successive layers of planar melaminium and adipate ions, with water molecules propagating hydrogen bonds by forming O−H⋅⋅⋅O contacts and as a donor for the melaminium N−H, together stabilising an extended hydrogen‐bonded organic network (Figure S1). Electrostatic interactions between melaminium and adipate ions likely provide additional structural stability. The small, positive ζ‐potential of **BMA** in water (+3.4 mV) indicates a near‐neutral surface termination (exposing both melaminium cations and adipate anions) and tendency to coalesce/crystallise. Physicochemical properties of **BMA** were determined by FTIR, solution and solid‐state NMR, powder X‐ray diffraction, potentiometric titration, FE‐SEM, TEM, and X‐ray photoelectron spectroscopy. Powder XRD indicated that **BMA** was obtained by the reaction of one equivalent of melamine with one, two or three equivalents of adipic acid; three equivalents of adipic acid gave the maximal yield with excess adipic acid removed by washing with water (in which **BMA** is insoluble).

^1^H NMR of **BMA** (recorded in DMSO‐*d_6_*) presented six ‐NH_2_ proton resonances as a singlet at 6.05 ppm, with a NH^+^ proton resonance at 2.07 ppm (Figure S3). Corresponding ^13^C NMR (Figure S4) identified four types of carbon atoms, assigned to the C=O of adipic acid/ adipate (174.3 ppm), −C=N− of melaminium (167.2 ppm), and aliphatic −CH_2_ species (33.3 and 23.9 ppm). Variable temperature ^1^H NMR (in DMSO‐*d_6_*) was performed using TMS (δ=0 ppm) as an internal reference to calculate the chemical shifts of protons engaged in hydrogen bonding.^[47]^ As expected, the ^1^H NMR signal for ‐NH_2_ protons of the melaminium cation shifted upfield (from ~6.10 ppm at 20 °C) on heating. The temperature‐dependence of this chemical shift (−4.84 ppb/°C) was indicative of a strongly hydrogen‐bonded ‐NH_2_ (Figure 1a‐b). A similar temperature dependence was observed for protons in lattice water molecules consistent with their participation in interlayer hydrogen bonding (Figure S6); higher temperatures promote movement of lattice water, which also results in a stronger ^1^H NMR signal. Protons in the −CH_2_ backbones of adipic acid/adipate were temperature‐invariant and hence not involved in hydrogen bonding. Complementary ^15^N cross polarisation‐magic‐angle spinning (CP‐MAS) NMR (Figure 1c) revealed three main peaks at −291, −279 and −263 ppm, assigned to exocyclic ‐NH_2_, protonated endocyclic ‐NH^+^ and unprotonated triazine ring nitrogen, respectively; the −279 ppm signal confirms that melamine is protonated in **BMA**.^[49]^ Powder XRD confirmed the successful synthesis of crystalline **BMA** (Figure 1d), with a weak reflection at 5.72° attributed to the melaminium cation (being absent in melamine) and an intense reflection at 25.83° attributed to the triazine core in accordance with a previous report.^[43]^ TGA showed the loss of lattice water at ~187 °C, followed by decomposition of the remaining organic constituents’ ≥365 °C (Figure S5). The FTIR spectrum of **BMA** confirmed the presence of melaminium and adipate ions: a ‐NH^+^ stretch was present at 2931 cm^−1^, with a melaminium ring deformation at ~715 cm^−1^.^[50]^ Additional bands at 1550 cm^−1^ and 1341 cm^−1^ are ascribed to ν_symm_ and ν_asymm_ modes of the adipate anion (Figure S2),^[43]^ while those attributed to melamine ‐NH_2_ and carboxylic acid ‐OH functions in **BMA** were shifted to higher wavenumbers compared to their respective precursors due to hydrogen bonding.

The acid strength and loading of **BMA** were quantified by potentiometry (Figure S7) and potentiometric titration with 0.01 M NaOH, respectively. **BMA** possessed a surface potential of 135 mV, lower than the potentials of 0.01 N HCl (278 mV) or H_2_SO_4_ (307 mV), or pure adipic acid (206 mV), but significantly more acidic than that of melamine (−30 mV). An acid loading of 3.534 mmol/g (Figure 1e), similar to our estimate of 3.44 mmol/g based on 1 proton per melamine unit (Figure 1c) was recorded. Unfortunately, it was challenging to quantify the basicity by similar methods, possibly due to the adsorption of atmospheric CO_2_.^[51]^

The acid character of **BMA** was also explored by DFT calculations comparing the electronic structure of free melamine, its conjugated acid melaminium (present in **BMA**) and the free energies of formation of some inorganic acids from their conjugate bases (Table S1). The melaminium ‐NH^+^ function is predicted to possess a similar acidity to HCl. Corresponding electrostatic potential maps of melamine and melaminium (Figure S8) revealed a uniform electrostatic potential distribution (pale green ~0 e^−^) in the case of unprotonated melamine, whereas the protonated ‐N triazine in **BMA** is electron deficient (blue region) and susceptible to nucleophilic attack (Figure S8b). Frontier molecular orbital analysis of melamine confirms that the HOMO of melamine extends across the triazine ring whereas the protonated triazine nitrogen in **BMAH** ^+^ is electron‐deficient (Figures S9‐S10).

Only carbon, nitrogen and oxygen were observed at the surface of **BMA** by XPS (Figure S11a), in the expected concentrations of 44.3 atom% C, 36.0 atom% N and 19.7 atom% O. The C 1s XP spectrum of **BMA** (Figure 1f) exhibited four chemical environments assigned to the C−C bond in adipic acid (284.54 eV binding energy), C−NH_2_ of melaminium (286.05 eV), C=N in the triazine ring (287.61 eV) and C=O functional groups in adipic acid/adipate (288.54 eV). The corresponding N 1s XP spectrum (Figure 1g) exhibited two chemical environments assigned to protonated triazine ring nitrogen (~401.9 eV), and exocylic ‐NH_2_ functions and unprotonated triazine ring nitrogen (398.9 eV) the latter species having similar binding energies.^[52]^ Two oxygen species in equimolar amounts were observed in the O 1s XP spectrum (Figure S11b), assigned to O=C−[O−] (532.4 eV) and [O] =C−O‐ (531.2 eV) in the adipate anion, and a third weaker species at 534.0 eV. The latter binding energy is similar to that for acid anhydrides, and hence is assigned to deprotonated oxygen in the adipate anion that are H‐bonded with adipic acid in **BMA**. FE‐SEM (Figure 1h and Figure S12) and TEM (Figure 1i) imaging of **BMA** revealed broad interconnected belt‐like crystals ~20 μm long and 5 μm wide, attributed to layers of melaminium adipate stabilised by adipic acid (Figure S1). EDX elemental mapping confirmed a uniform distribution of C, N and O throughout the crystals (Figure S12).

### One‐Pot Catalytic Cascade

**Figure 2:**
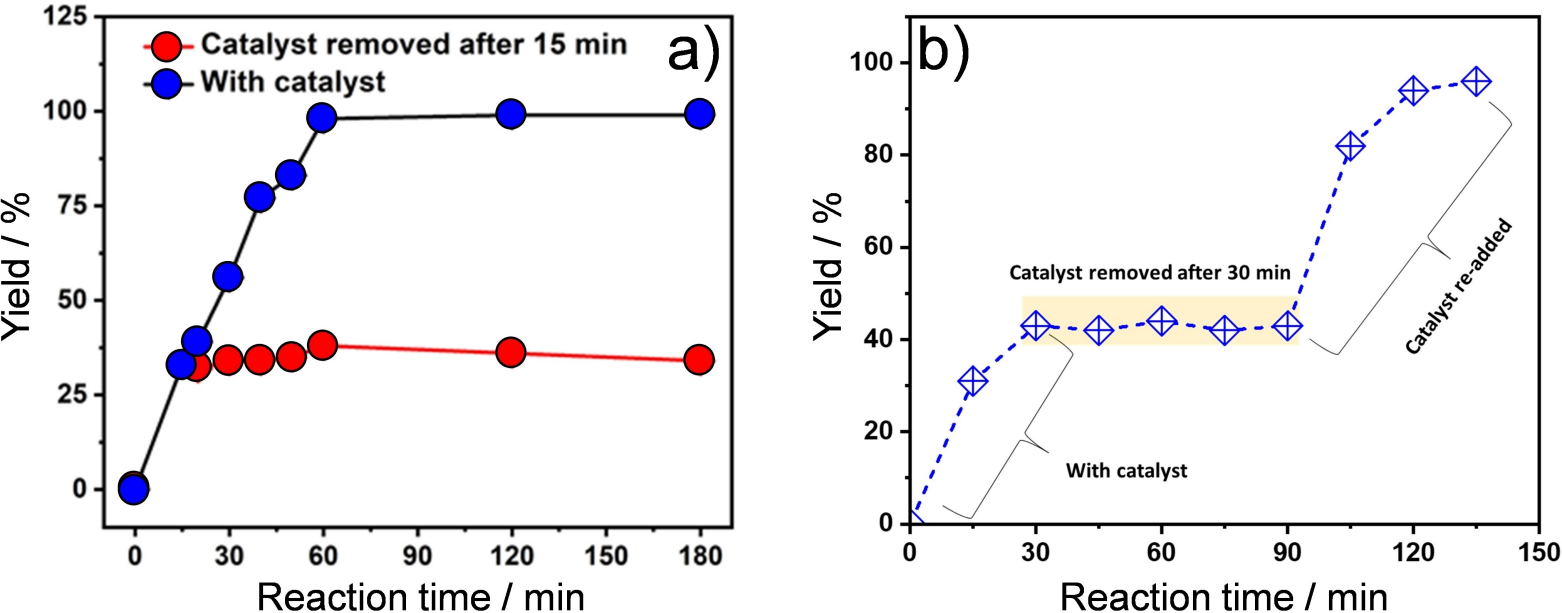
(a) Time‐dependent benzylidenemalononitrile production from benzaldehyde dimethyl acetal catalysed by **BMA** in water at 27 °C and (b) catalyst heterogeneity test.

Spatially orthogonal, bifunctional catalysts can be advantageous for chemical cascades, offering higher activity and selectivity to random arrangements of active sites or physical mixtures of individual catalysts.[^2^, ^54^] The spatial localisation of acid (protonated triazine N) and base (exocyclic ‐NH_2_) sites within the melaminium core of **BMA** was therefore assessed for the one pot deacetalisation of substituted acetals and subsequent Knoevenagel condensation of the resulting aldehydes with malononitrile, an attractive route to C−C bond formation in the synthesis of pharmaceuticals and natural products.^[55]^ Equimolar amounts (1 mmol each) of benzaldehyde dimethyl acetal and malononitrile were treated in water at room temperature for 1 h with 10 mg **BMA** (581 mol.g^−1^ molar mass, equating to 0.017 mol % catalyst); these being the optimal reaction conditions (Table S2). Benzylidenemalononitrile was isolated as white precipitate (~90 %),^[46]^ which was extracted in CHCl_3_, dried and quantified by ^1^H NMR (~98 %). No benzylidenemalononitrile product of the two‐step cascade was observed in the absence of **BMA**, or in the presence of **BMA** precursors (adipic acid and melamine individually or in combination). In contrast, under optimal conditions, **BMA** delivered a ~99 % yield of the desired benzylidenemalononitrile in 1 h (Figure 2 and Table S2, entry 4). The linear dependence of benzylidenemalononitrile production with reaction time indicates minimal catalyst deactivation. Catalyst heterogeneity was assessed by filtration of **BMA** from the reaction mixture after 15 min (Figure 2a); no further product was observed consistent with the insolubility of **BMA** in the reaction medium. Catalyst removal at 30 min and re‐addition at 90 min (Figure 2b) confirmed a heterogeneous process ; similar reaction profiles for the initial catalytic period and after **BMA** re‐addition evidencing negligible deactivation.

**Figure 3:**
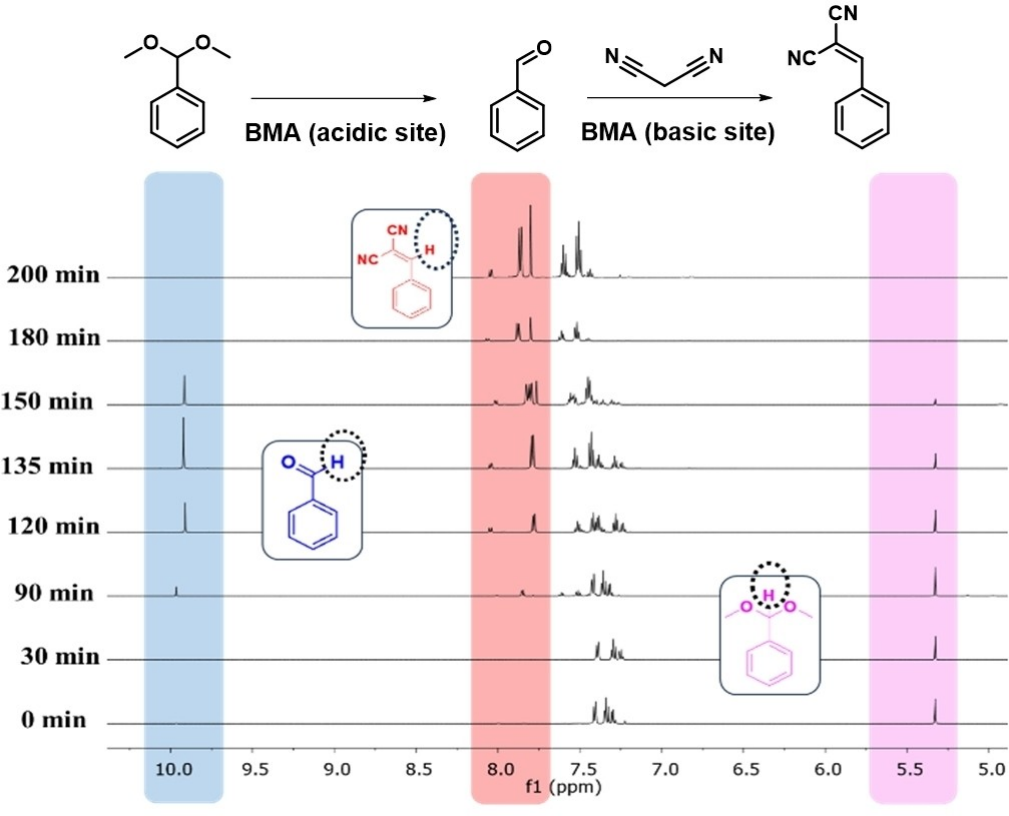
Time‐dependent ^1^H NMR spectra of **BMA** catalysed cascade reaction in CDCl_3_ at room temperature.

Time‐dependent consumption of the acetal, formation of the benzaldehyde intermediate, and reaction of the latter with malononitrile to form benzylidenemalononitrile were also directly studied by ^1^H NMR, using CDCl_3_ rather than water as the solvent (Figure 3). Note: reaction was slower in CDCl_3_ than water. Complete loss of the acetal (~5.3 ppm) occurred within 150 min, coincident with appearance of the benzaldehyde intermediate (~9.9 ppm) and benzylidenemalononitrile product (7.8 ppm) at 90 min. The benzaldehyde concentration peaked at 135 min before rapidly decreasing to zero ≥180 min, whereas the benzylidenemalononitrile concentration increased monotonically with reaction time.

**Figure 4:**
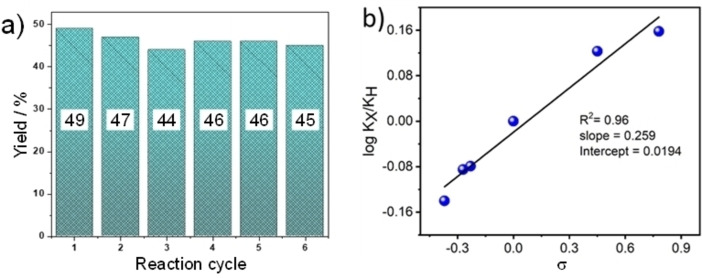
(a) Benzylidenemalononitrile yield versus reaction cycle (reactions were stopped at 50 % diacetal conversion). (b) Hammett plot illustrating linear free‐energy relationship between equilibrium ionisation constants of the para‐substituents and H, respectively, and Hammett substituent constants of substituted acetals.

Stability of the **BMA** catalyst was assessed over six consecutive reaction cycles (Figure 4a). Each reaction was intentionally only run to 50 % acetal conversion to ensure any deactivation would be detectable and not masked by mass‐transport limitations. After each reaction, the **BMA** powder was collected by filtration, washed with water and subsequently methanol to remove any organic residues, and air‐dried before adding to a fresh reaction mixture. The benzylidenemalononitrile yield only decreased by a maximum of 5 % (from a theoretical maximum of 50 %) over five recycles, evidencing excellent stability, confirmed by XRD and ^1^H NMR which exhibited negligible differences between the as‐prepared **BMA** and that recovered after six cycles (Figures S13‐S14).

Turnover frequencies (TOFs) for both steps of the cascade were calculated for the optimal reaction conditions, assuming an equal acid and base site loading (only the former being experimentally quantifiable). Deacetalisation occurred with a TOF of 29 h^−1^. This value is much lower than the 320 h^−1^ reported over a spatially orthogonal hierarchically porous acid‐base catalyst, however the hierarchically porous catalyst contained superacidic sulfated zirconia and reactions were performed at 50 °C (expected to increase the rate four‐fold relative to the present study). Literature benchmarking reveals significant performance advantages for **BMA** in cascade deacetalisation‐condensation reactions (Table S3), notably the greatly reduced catalyst loading (only 0.017 mol %), temperature (25 °C) and reaction time (1 h) to complete both steps. Advantages that complement the simple one‐pot synthesis of **BMA** from low cost and abundant precursors. A control reaction between benzaldehyde and malononitrile under the optimised conditions achieved ~97 % benzylidenemalononitrile in only 30 min, demonstrating that the base‐catalysed Knoevenagel condensation was twice as fast (TOF of 56 h^−1^) as the acid‐catalysed deacetalisation.

The substrate scope of **BMA** was further investigated for a range of aromatic and heterocyclic acetals (Table 1). In all cases, benzylidenemalononitrile products were obtained as solid powders and identified by CHNS and NMR (see Supporting Information), with the majority isolated in >80 % yields. Electron‐withdrawing substituents (O, halide, carboxylate etc.) resulted in the highest yields (>85 %), whereas electron‐donating substituents (−NMe_2_ etc.) resulted in more modest yields (>62 %). The good to excellent yields obtained for heterocyclic compounds illustrates the suitability of **BMA** for transforming biologically relevant substrates.^[56]^ The electronic effect of different substituents on the reactivity was examined by a Hammett correlation study (Figure 4b). A plot of log (K_X_/K_H_), where K_X_ and K_H_ are the ionisation constant of the para‐substituents and H, respectively, versus Hammett substituent constants σ (Table S4) was linear with a slope of 0.259, evidencing the applicability of the Hammett linear‐free‐energy relationship.^[57]^

**Table 1:** Substrate scope of deacetalisation‐Knoevenagel condensation over **BMA**.^[a]^

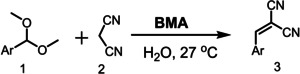
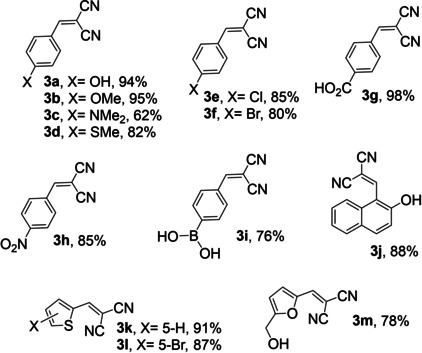

^[a]^ Reaction conditions: 1 mmol of acetal and malononitrile, 10 mg **BMA**, 3 mL water and 1 h.

### Reaction Mechanism

**Figure 5:**
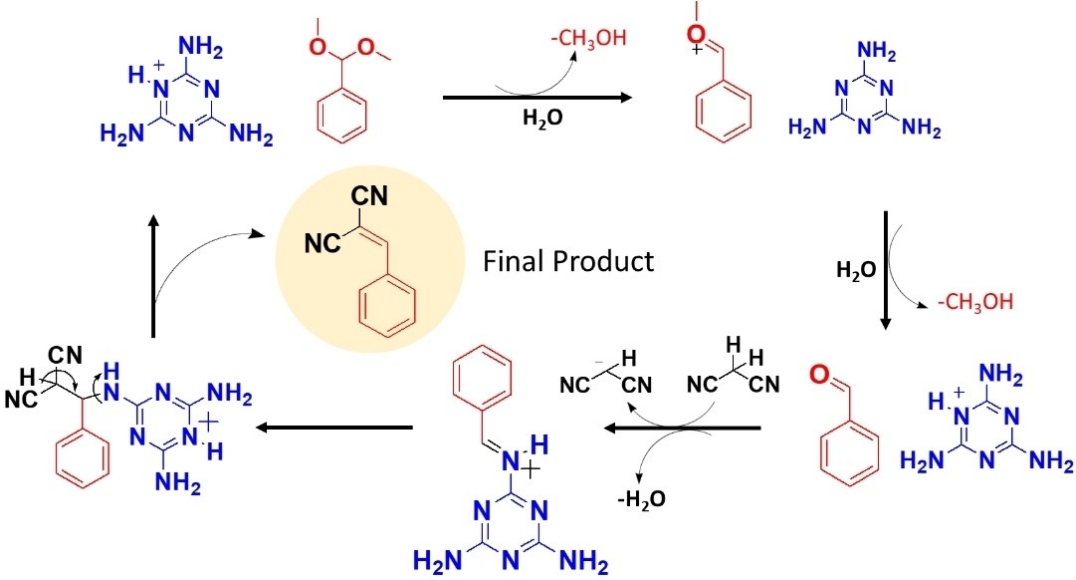
Possible deacetalisation‐Knoevenagel condensation mechanism over **BMA** melaminium cation.

Our hypothesis that the protonated component of **BMA** (a melaminium cation) is responsible for both steps of the catalytic cascade was subsequently explored by DFT computational modelling. Electrostatic mapping of the frontier orbitals in the melaminium cation (Figures S8‐S9) evidence that the exocyclic ‐NH_2_ group, para to the (Brønsted acidic) protonated N atom, is electron‐rich and hence should exhibit Lewis basicity. A mechanism can thus be postulated involving: (i) demethoxylation of an acetal at the Brønsted acidic NH^+^ centre of melaminium; and subsequent (ii) dehydration of the reactively‐formed benzaldehyde and (iii) carbon‐carbon bond‐forming with deprotonated malononitrile at the exocyclic ‐NH_2_ group (Figure 5).

**Figure 6:**
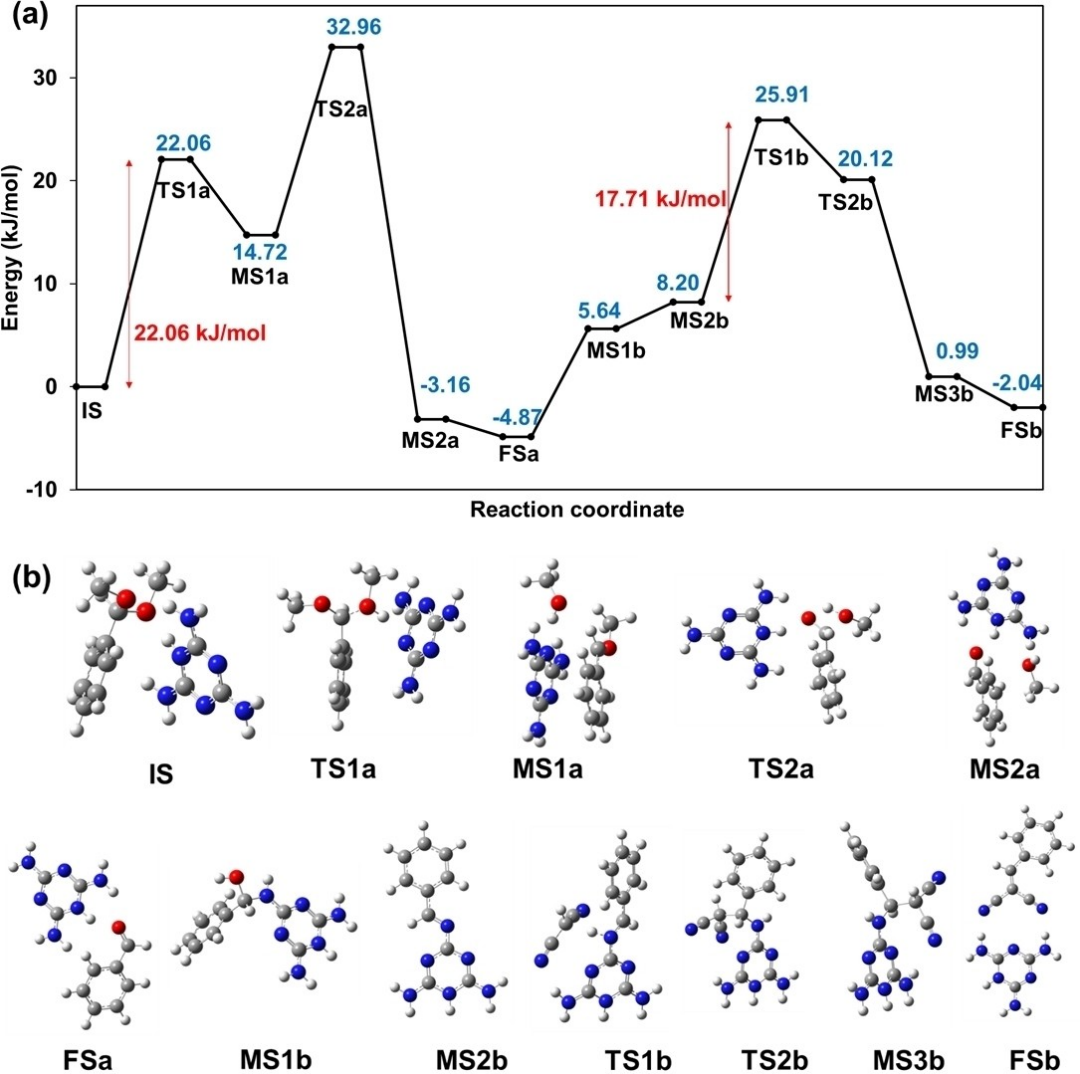
(a) Computed free energy profile for the deacetalisation of benzaldehyde dimethyl acetal over a melaminium cation, and subsequent Knoevenagel condensation with malononitrile to form benzylidenemalononitrile. (b) Initial state (IS), metastable intermediate state (MS), transition state (TS), step 1 product (FSa) and step 2 product (FSb) structures. Free energy values in kJ.mol^−1^.

A computed free energy profile and optimised structures (based on the reaction intermediates in Figure 5) are illustrated in Figure 6 and Figure S15. In the initial state (IS), a melaminium cation reacts with benzaldehyde dimethyl acetal to eliminate methanol and form an oxonium intermediate (MS1a) via a transition state (TS1a, energy barrier ~22 kJ.mol^−1^). This oxonium intermediate possesses an activated carbonyl carbon centre that is highly electron deficient and susceptible to nucleophilic attack by the water solvent in an endergonic reaction (~18 kJ.mol^−1^) to form a new transition state (TS2a). Loss of a second methanol unit (MS2a), results in benzaldehyde and regeneration of the melaminium cation (FSa); the overall reaction is exergonic by nearly 5 kJ.mol^−1^. Benzaldehyde then reacts with the melaminium cation (MS1b) to eliminate water and form an imine intermediate (MS2b). Malononitrile interacts with the imine‐melaminium intermediate through a first transition state (TS1b, energy barrier ~18 kJ.mol^−1^) which undergoes resonance stabilisation with another transition state (TS2b, exergonic by ~6 kJ.mol^−1^) in which benzylidenemalononitrile is formed (MS3b) and subsequently released to regenerate the melaminium cation (FSb). The first acetal demethoxylation (TS1a) is predicted to be rate limiting for the overall cascade, in accordance with our experimental TOFs for deacetalisation (29 h^−1^) versus condensation (56 h^−1^).

**Figure 7:**
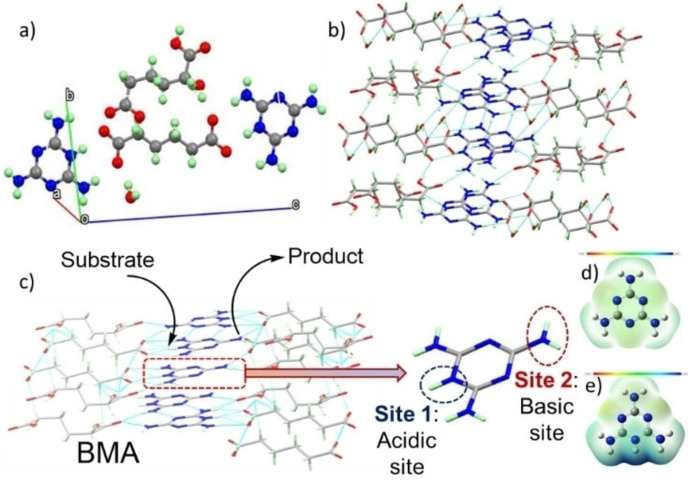
(a) Unit cell of crystalline **BMA**, (b) hydrogen bonding network between adipic acid and melamine within crystal, (c) schematic of acid‐base catalysis and associated active sites at melamine, and electron density across (d) melamine (from −0.2 eV in red to +0.2 eV in blue) and (e) a melaminium cation (from 0.0 eV in red to +0.25 eV in blue): red=electron‐rich and blue=electron‐deficient.

A schematic representation of the catalytic cascade over the acid and base sites within **BMA** is presented in Figure 7 wherein the acidic triazine ring proton promotes deacetalisation and exocyclic amines promote condensation.

### Green Metrics

The sustainability and the environmental impact of the catalytic process was evaluated from a range of metrics (calculations detailed in the Supporting Information).^[58]^ The E‐factor of 0.024 was extremely low (neglecting water as a waste product), the atom‐economy >89 %, the mass intensity ~1, the process mass intensity (which includes the mass of water solvent ~1.8, and the reaction mass intensity ~98 % (Table S5). Collectively these demonstrate an economic and effective reaction, which requires no reagents and generates minimal waste.

## Conclusions

The hydrothermal synthesis of a charge‐assisted, hydrogen‐bonded bis(melaminium)adipate (**BMA**), organocatalyst and its catalytic application to an ambient, aqueous phase acid‐base cascade reaction is reported. Density functional theory (DFT) modelling predicts that protonation of the parent melamine precursor is thermodynamically favoured in water, resulting in an electron‐deficient (Brønsted acidic) protonated N in the triazine ring of the melaminium cation, in close proximity to an exocylic Lewis base primary amine. The presence of antagonist acid‐base sites in the same organocatalyst was exploited for the cascade transformation of (aromatic) dimethyl acetals to benzylidenemalononitriles. Time‐dependent ^1^H NMR measurements, kinetic studies and DFT modelling, evidence that the reaction of benzaldehyde dimethyl acetal over **BMA** proceeds via: (i) deacetalisation at the Brønsted acid site (eliminating methanol and forming an oxonium intermediate) to form benzaldehyde; (ii) formation of an imine intermediate (by Knoevenagel condensation) between reactively‐formed benzaldehyde and the exocyclic NH_2_ of the melaminium cation; and (iii) subsequent interaction of malononitrile with the imine‐melaminium intermediate to form the benzylidenemalononitrile product. The first acetal demethoxylation is predicted to be rate‐limiting for the overall cascade, with a predicted activation barrier of 22 kJ.mol^−1^ versus 18 kJ.mol^−1^ for the condensation step, consistent with experimentally determined turnover frequencies of 29 h^−1^ versus 56 h^−1^.

A broad substrate scope was demonstrated, with electron‐withdrawing substituents resulting in the highest product yields (>85 %), whereas electron‐donating substituents resulted in more modest yields (>62 %). Heterocyclic aromatic malononitrile products were also obtained in high yields (>78 %) unlocking catalytic routes to bioactive molecules. Substrate reactivity over the **BMA** catalyst was predicted by Hammett linear‐free‐energy relationships. Excellent catalyst stability was observed, with 90 % of the original acetal yield from benzaldehyde dimethyl acetal obtained following six consecutive reaction cycles (with simple solvent washing between cycles). The high intrinsic activity of **BMA** permits very low catalyst loadings and extremely mild reaction conditions (ambient, air, in water), resulting in very high sustainability metrics for the cascade deacetalisation and Knoevenagel condensation, with water as the only by‐product. We envisage hydrogen‐bonded organocatalysts will find broad application as low‐cost, energy‐ and atom‐efficient heterogeneous catalysts for the sustainable chemical manufacturing of fuels and chemicals.

## Supporting Information Summary

Additional experimental details and characterisation of the catalyst and molecular species are available as supporting information.

## Author Contributions

AC: methodology (BMA synthesis, characterization, cascade reaction), formal analysis, data curation, writing original draft; AM: methodology, formal analysis (DFT calculations), data curation, validation; SM: supervision (AC), writing‐ review & editing; RB: supervision (AM), writing‐ review & editing; KW: fund acquisition, supervision (AC), writing – review & editing; AFL: fund acquisition, supervision (AC), writing – review & editing; JM: fund acquisition, supervision (AC), writing – review & editing.

## Conflict of Interests

The authors declare no competing interests.

1

## Supporting information

As a service to our authors and readers, this journal provides supporting information supplied by the authors. Such materials are peer reviewed and may be re‐organized for online delivery, but are not copy‐edited or typeset. Technical support issues arising from supporting information (other than missing files) should be addressed to the authors.

Supporting Information

## Data Availability

The data that support the findings of this study are available from the corresponding author upon reasonable request.
