# Supplementary material for: A Heterogeneous Acid‐Base Organocatalyst For Cascade Deacetalisation‐Knoevenagel Condensations
Source: ChemSusChem. 2024 Sep 3;17(24):e202400866. doi: 10.1002/cssc.202400866 (PMC11660745; doi:10.1002/cssc.202400866)
Supplement: Supplementary file 1 — Supporting Information [file CSSC-17-e202400866-s001.pdf]

ChemSusChem

Supporting Information

A Heterogeneous Acid-Base Organocatalyst For Cascade Deacetalisation-Knoevenagel Condensations

Ashis Chhetri, Ashakiran Maibam, Subashani Maniam, Ravichandar Babarao,* Karen Wilson,* Adam F. Lee,* and Joyee Mitra*

A heterogeneous organocatalyst containing antagonistic acid-base sites for cascade synthesis of benzylidenemalononitriles

Ashis Chhetri, [a,b,c] Ashakiran Maibam,[b,d,e] Subashani Maniam,[c] Ravichandar Babarao,*[e,f] Karen Wilson,*[g] Adam F. Lee,*[g] Joyee Mitra*[a,b]

[a]Inorganic Materials & Catalysis Division, CSIR-Central Salt & Marine Chemicals Research Institute, Gijubhai Badheka Marg, Bhavnagar 364002, Gujarat, India.

[b]Academy of Scientific and Innovative Research (AcSIR), Ghaziabad 201002, India.

[c]School of Science, STEM College, RMIT University, Melbourne, VIC, 3000, Australia.

[d]Physical and Materials Division, CSIR-National Chemical Laboratory Pune 411008, India.

[e]Centre for Advanced Materials and Industrial Chemistry (CAMIC), RMIT University, Melbourne, VIC, 3000, Australia.

[f]CSIRO, Clayton VIC 3168, Australia.

[g]Centre for Catalysis and Clean Energy, School of Environment and Science, Griffith University QLD 4222, Australia.

Pages: 35 (S1-S35)

Figures: 47 (S1-S47)

Tables: 5 (S1-S5)

Experimental details

Chemicals

Melamine (99 %), adipic acid (99 %), benzaldehyde dimethyl acetal (99 %), benzaldehyde (99 %), DMSO-d_6 (99.8 %) and $CDCl_3$ (99 %) were purchased from Sigma Aldrich whereas malononitrile (98 %) was purchased from Spectrochem and were used without further purification.

Synthesis

BMA was synthesised by adapting the literature protocol in **Scheme 1**.[1] Melamine (0.001 mol, 0.1261 g) and adipic acid (0.003 mol, 0.4384 g) were thoroughly mixed by grinding in an agate mortar pestle. The resulting solid mixture was transferred to a Teflon bomb to which 15 mL of deionised water was added. The autoclave was then placed in an oven and heated at 160 °C for 72 h. After cooling, colourless block-shaped crystals were obtained by gravity filtration, thoroughly washed with deionised water and air dried overnight. The isolated yield of the crystals was ~85 % on a molar basis.

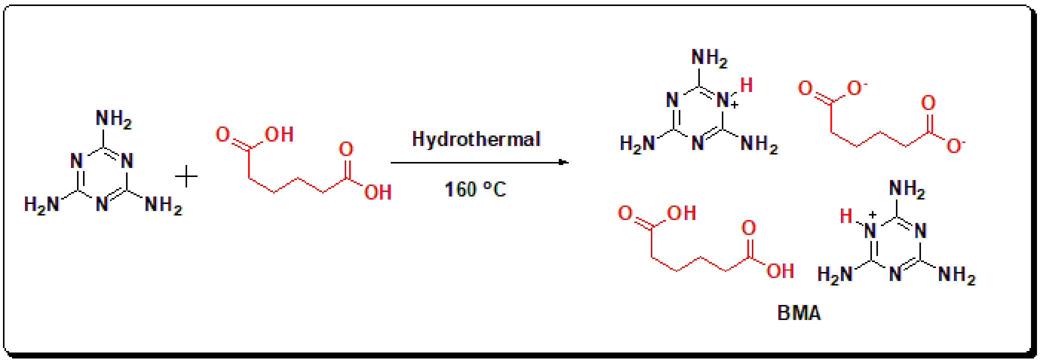

Scheme S1. Hydrothermal synthesis of **BMA**.

Characterisation

Powder X-ray diffractograms were recorded on a PANalytical X'PERT-PRO diffractometer with Cu K_α radiation (λ = 1.78901 Å). Data were collected from 5 to 50° with 0.013° steps. Surface morphologies were analysed using a JEOL FE-SEM with an accelerating voltage of 30 kV equipped with energy dispersive X-ray analysis (EDX). X-ray photoelectron spectroscopy (XPS) was performed on a Thermo Fisher Scientific ESCA-LAB Xi+ instrument with 50 mV pass energy. Binding energies were referenced against adventitious carbon at 284.8 eV, with high-resolution spectra fitted to a mixed Gaussian-Lorentzian lineshape (70-30%) and common FWHM for all components of each element. CP-MAS NMR spectra were recorded on an Oxford Instruments NMR AS500 Magnet with Agilent DD2 Console. The ^{15}N CP-MAS NMR spectrum of BMA was collected over 1024 scans acquired in 1.1 h. Thermogravimetric analysis was performed using a Mettler-Toledo TGA/DSC instrument under a constant flow of 5 mL.min^{-1} Ar while ramping at 5 °C.min^{-1} from 50-800 °C.

Computational modelling

Density Functional Theory (DFT) calculations were performed using the Gaussian 09 software package.[2] Structures were optimised using the B3LYP functional, with electronic interactions represented with the Grimme's D2 dispersion correction and a 6-311G+(d,p) basis set for all elements.[3] Solvent effects were incorporated for Conductor-like Polarizable Continuum Model (CPCM) model of water as implemented in the software.[4] Frequency calculations were performed to confirm that optimised structures exhibited non-negative vibrational frequencies, and transition states exhibited imaginary vibrational frequencies. The free energy profile for reaction coordinates was calculated by incorporating zero-point energy corrections for all the optimised structures at 298.15 K.

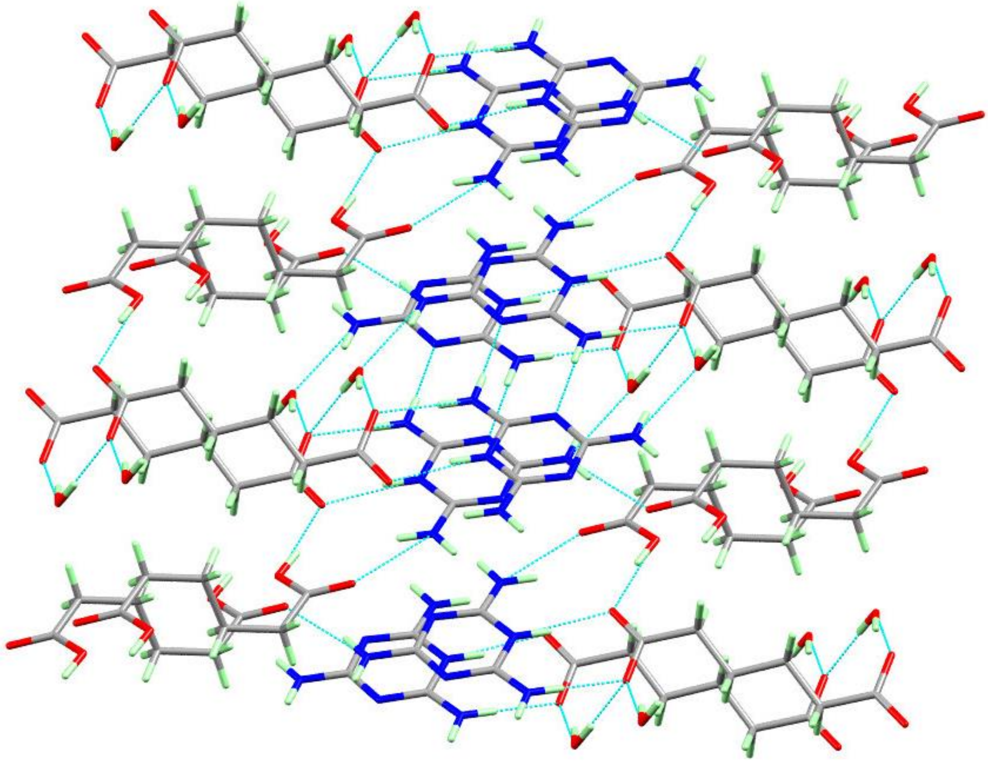

Figure S1. Adipate-adipic acid mediated hydrogen-bonded structure of **BMA** derived by single crystal XRD (from *ACS Appl. Polym. Mater.* **2021**, *3*, 651-660).

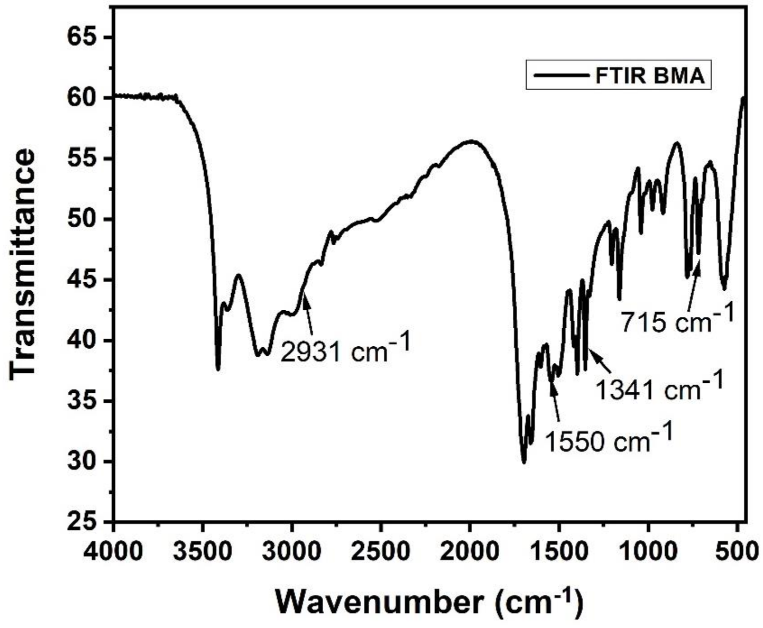

Figure S2. FTIR spectrum of **BMA**.

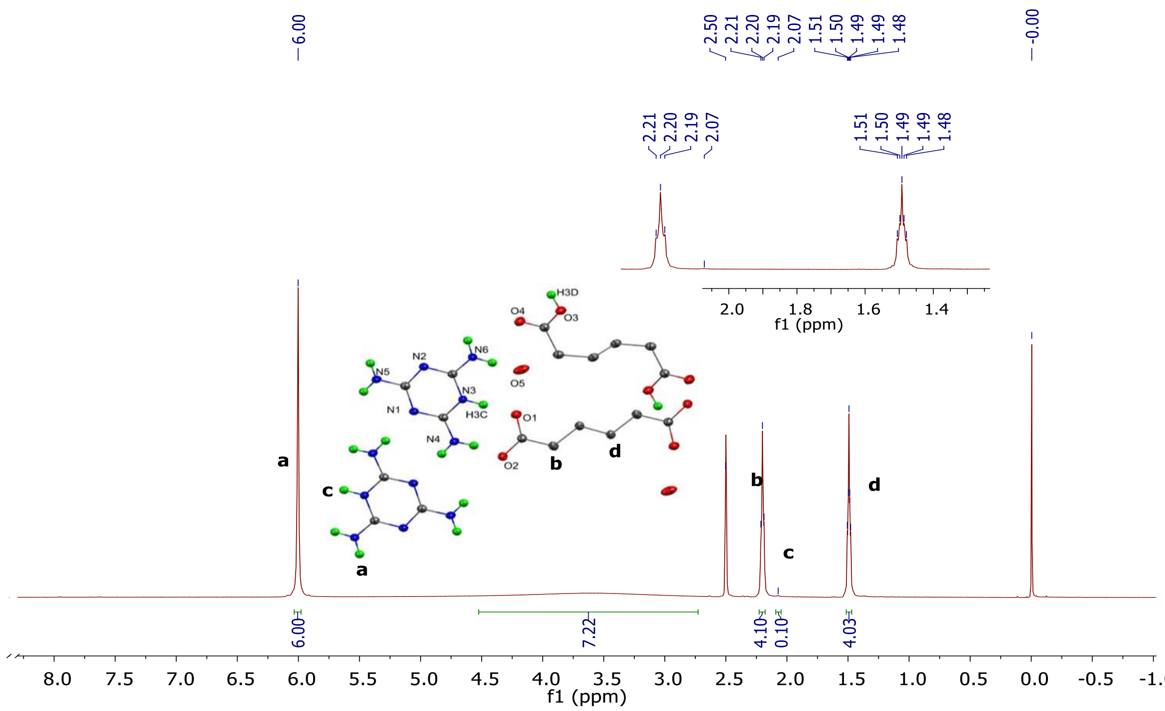

Figure S3. ^{1}H NMR of **BMA** in DMSO-d_6.

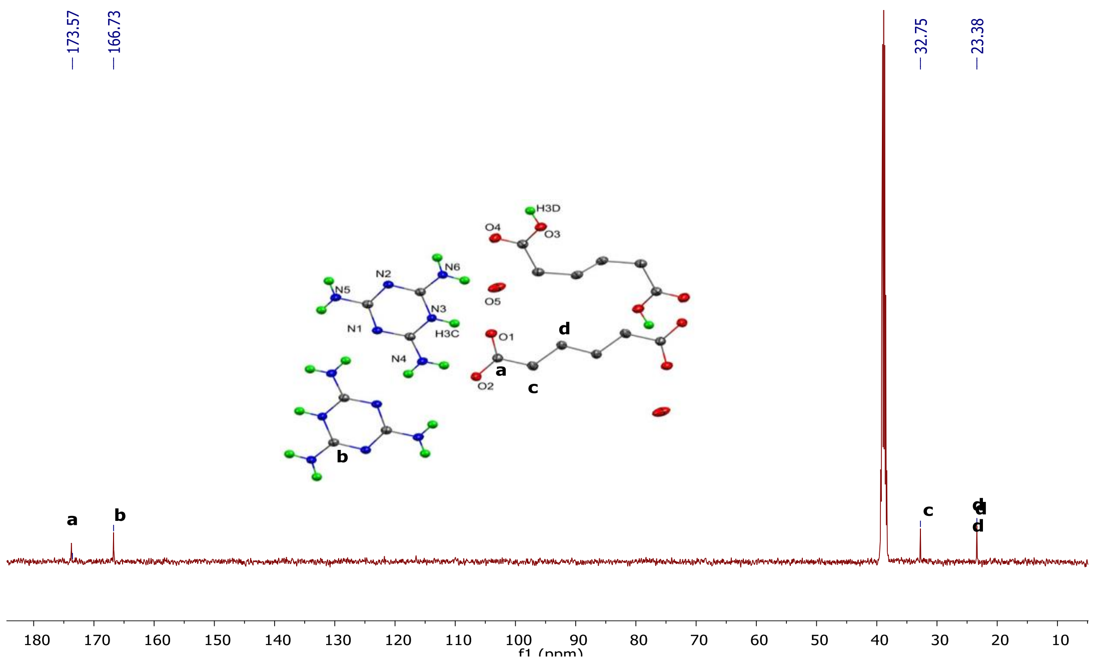

Figure S4. ^{13}C NMR of **BMA** in DMSO-d_6.

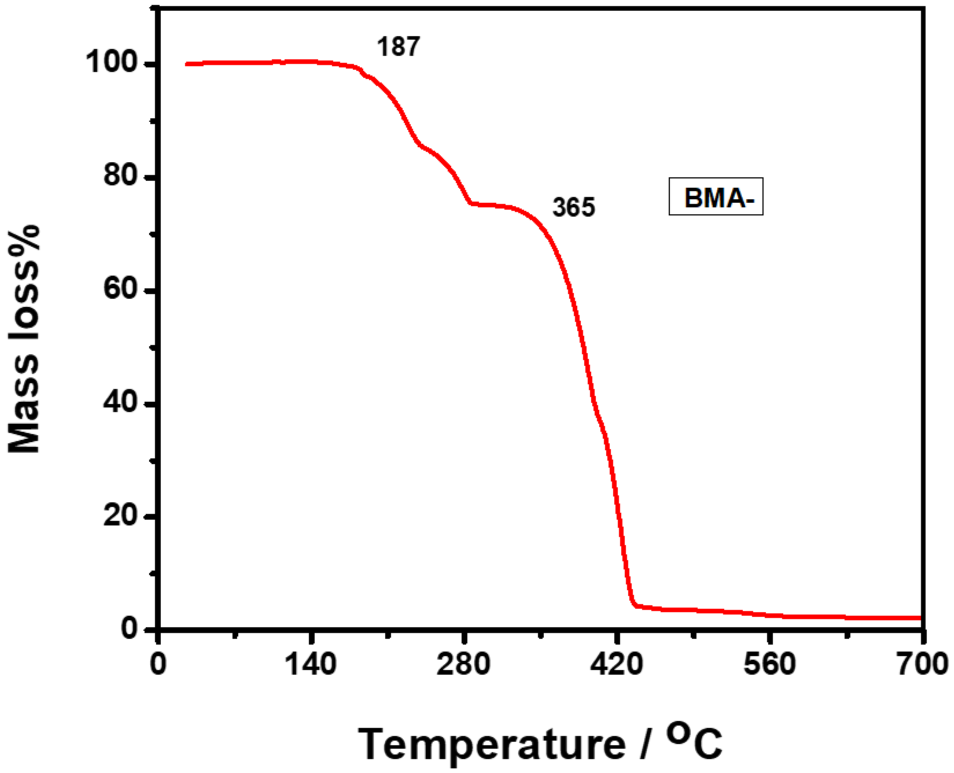

Figure S5. Thermogravimetric analysis of **BMA**.

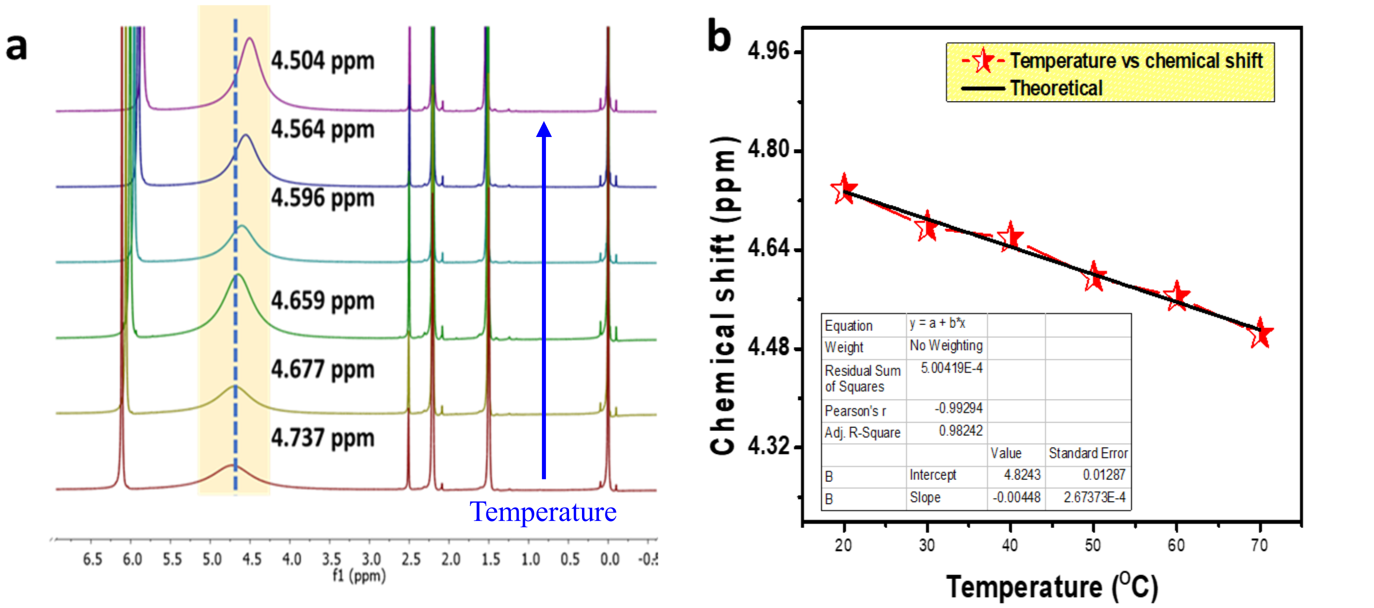

Figure S6. (a) Upfield shift of [1] H NMR signal from lattice H_2O molecules, and (b) chemical shift (δ ppm) dependence of corresponding protons. Theoretical values obtained from linear least-squares fitting.

Potentiometry

Acidic properties of **BMA** were quantified by potentiometry using a Mettler Toledo G10s Compact Titrator. The DGi115-SC electrode was calibrated against pH 4.02, 7.00 and 10.02 buffer solutions at 25 ±0.5 °C; the resulting response slope was in the standard range (-55 mV/pH to -65 mV/pH). Potentiometric curves were also determined for a range of acids and **BMA** precursors. In each case, 50 mg of solid or liquid acid (0.01 N) were dispersed in 50 mL deionised water, and the resulting time-dependent potentials recorded until steady state was attained (see below). The acid loading in **BMA** was also determined by titration using 0.01 M NaOH and potassium hydrogen phthalate (KHP, 8 mg in 50 mL) as a reference.

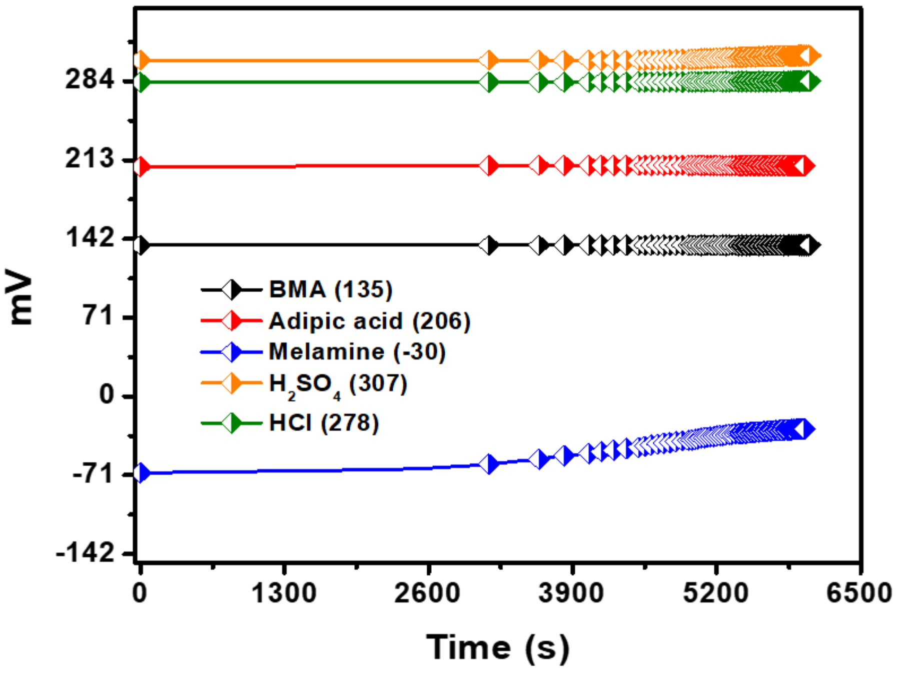

Figure S7. Potentiometric time profiles for **BMA**, its precursors and selected mineral acids.

Table S1. Free energy of formation of few inorganic and organic acids from their conjugate bases.

Conjugate base		Acid	Free energy of formation
HSO_4^-	⟶	H_2SO_4	**ΔG = 5.01 eV**
Cl^-	⟶	HCl	**ΔG = 4.68 eV**
CH_3COO^-	⟶	CH_3COOH	**ΔG = 3.70 eV**
Melamine	⟶	Melamine-H^+	**ΔG = 4.27 eV**

The relative trend in their acidic strength follows, H_2SO_4 > HCl> Melamine-H^+ > AcOH

Molecular electrostatic potential maps of **BMA** and **BMAH**$^+$ (**Figure S8**) shows a nearly neutral and uniform electrostatic potential distribution (pale green ~ 0 e) in the case of **BMA**, whereas the protonated -NH triazine in **BMAH**$^+$ is electron-deficient (blue region), and hence susceptible to nucleophilic attack.

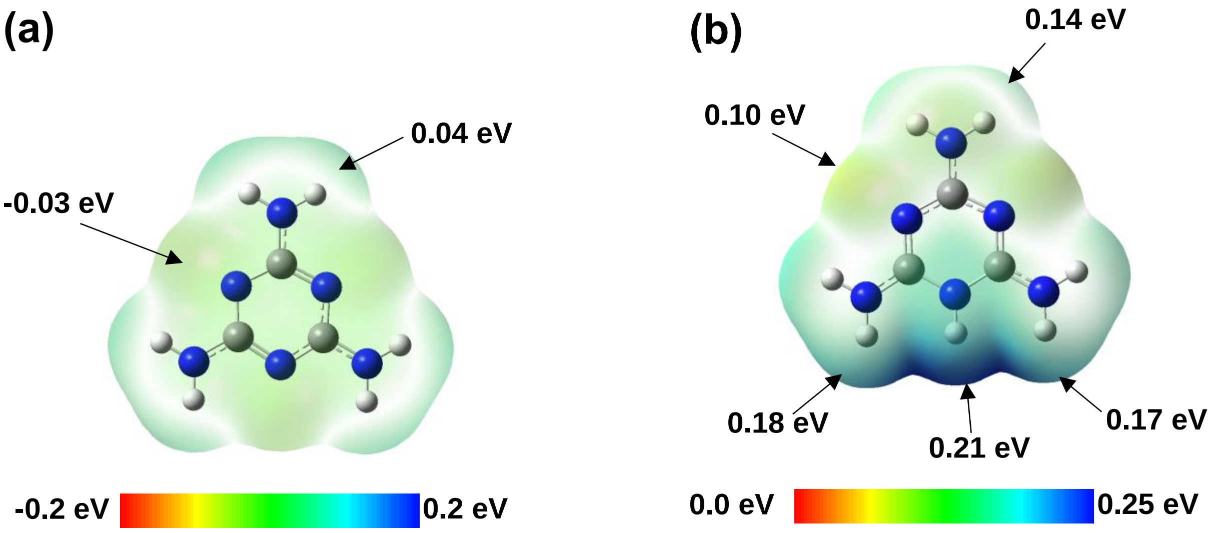

Figure S8. Molecular electrostatic potential (MEP) maps of (a) melamine/**BMA** and (b) melaminium ion/**BMAH**$^+$. Negative regions are related electron-rich and positive regions are electron-deficient.

Frontier molecular orbitals analysis of **BMA** confirm a high electron density in the triazine ring (**Figure S9**. HOMO), and missing electron density from the protonated -NH triazine of **BMAH**$^+$ (**Figure S10**, HOMO).

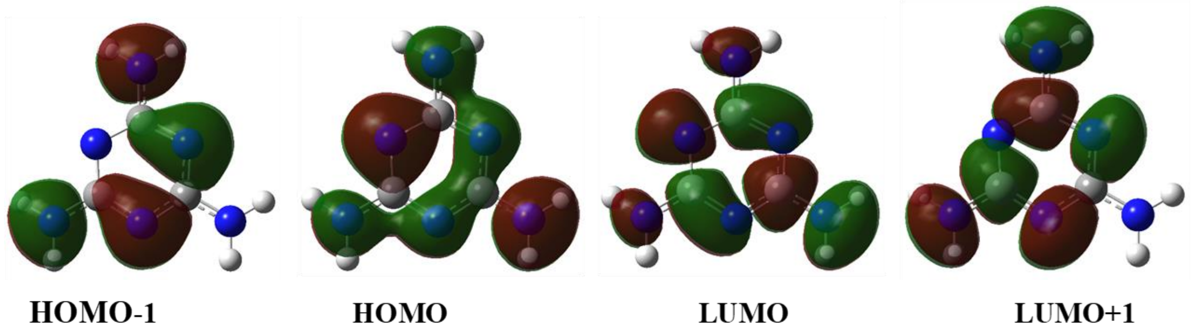

Figure S9. Highest Occupied Molecular Orbital (HOMO) and Lowest Unoccupied Molecular Orbital (LUMO) of melamine.

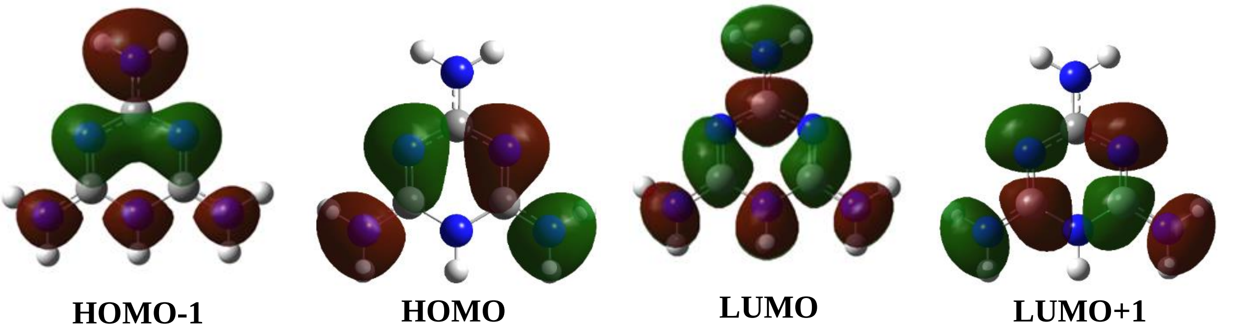

Figure S10. Highest Occupied Molecular Orbital (HOMO) and Lowest Unoccupied Molecular Orbital (LUMO) of the melaminium ion in **BMA**.

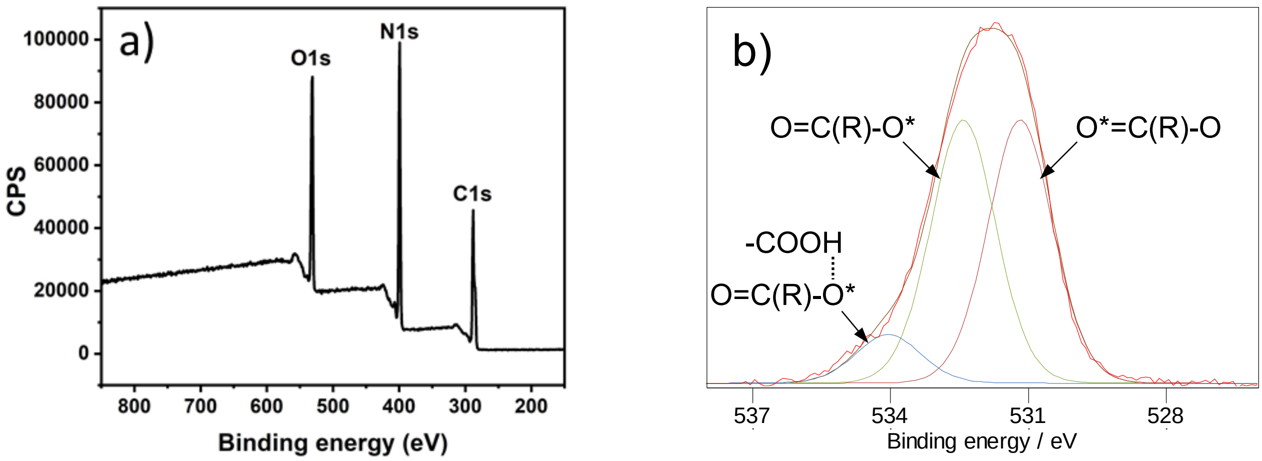

Figure S11. (a) XPS survey spectrum, and (b) fitted O 1s XP spectrum of **BMA**.

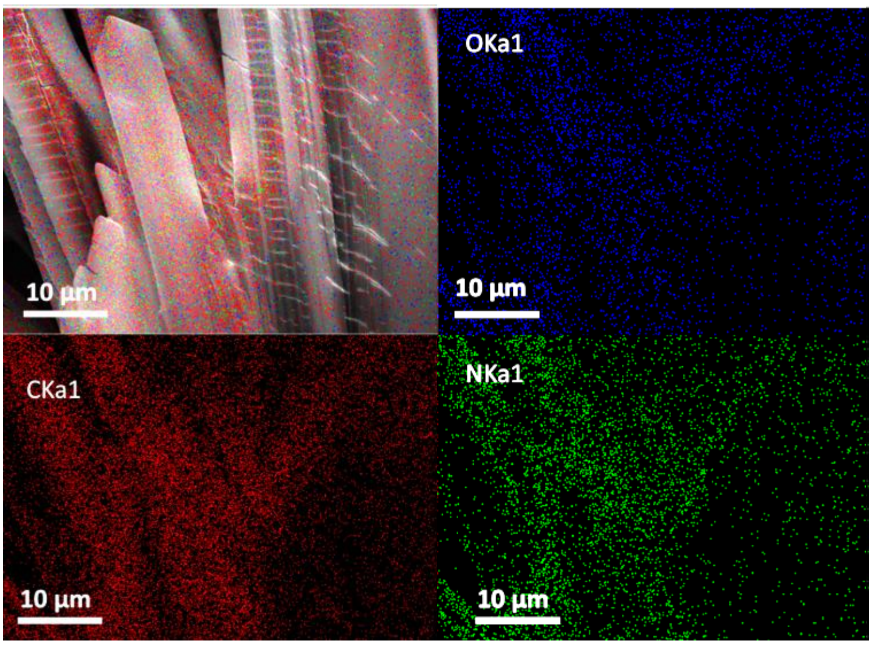

Figure S12. (top left) FE-SEM image of **BMA** crystals and (top right and bottom) corresponding EDX elemental maps evidencing a uniform distribution of C, N and O as expected for a co-crystal.

Deacetalisation-Knoevenagel condensation protocol

1 mmol of BDMA and 1.02 mmol of malononitrile were added to a glass vial containing 3 mL of DI water and the solution was stirred at room temperature for 5 min under air. Subsequently, 10 mg of **BMA** (0.0207 mmol) were added to the mixture stirring continued for 1 h. The initially colourless solution turned pale yellow, possibly due to formation of the benzaldehyde intermediate before producing an off-white precipitate. The precipitate was collected, extracted in $CHCl_3$, and dried over anhydrous Na_2SO_4 to form an off-white powder. The final product yield was calculated by ^{1}H NMR (98 %) and was in good agreement with the isolated yield (>90 %).

Calculation of yield and conversion

Characteristic methoxy peaks were observed for benzaldehyde dimethylacetal (δ 5.3 ppm), a -CHO peak for the intermediate benzaldehyde (10.0 ppm) and peak for the benzylidenemalononitrile product (8.5-8.7 ppm) in DMSO-d_6. Conversion and yield were calculated by integration of the benzylic protons of benzaldehyde dimethylacetal (a), benzaldehyde (b) and benzylidenemalononitrile (c) as below:

Conversion / % = (b+c)/(a+b+c) × 100

Yield / % = c/(a+b+c) × 100

Table S2. Optimisation of reaction conditions for deacetalisation-Knoevenagel condensation.

Entry	Catalyst loading / mg	Reaction time / min	Yield[a] / %
1	25	300	~99
2	25	60	~99
3	20	60	~99
4	10	60	~98
5	10	45	80
6	10	30	56
7	10	20	39
8	10	15	33
9	5	60	86
10	5	120	92
11	5	180	93
12[b]	--	180	--
13	25 mg adipic acid	180	--
14	25 mg melamine	180	trace

[a]From ^{1}H NMR. [b]No catalyst. Reaction conditions: 1 mmol benzyaldehyde dimethyl acetal, 1.02 mmol malononitrile, **BMA** catalyst (unless otherwise stated), 3 mL solvent deionised waster, room temperature.

Catalyst recycling

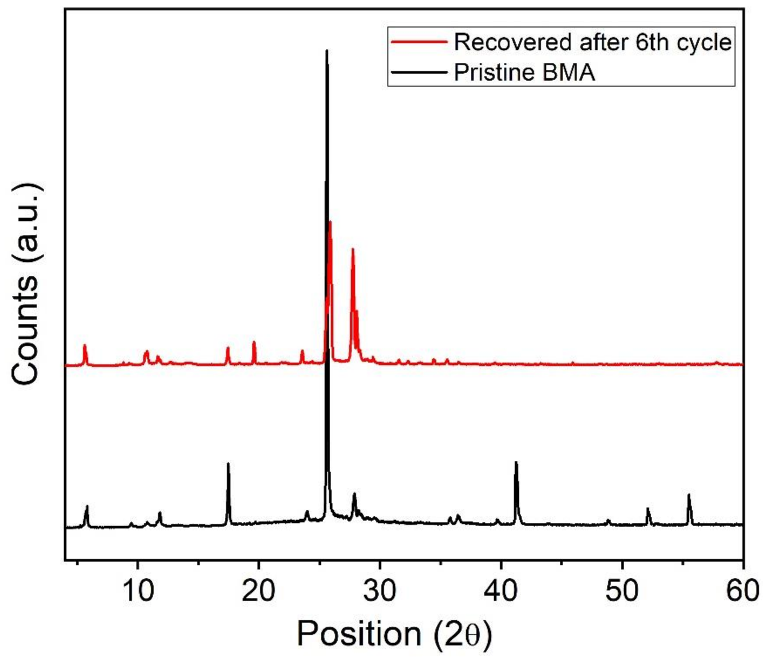

Figure S13. Powder X-ray diffractograms of as-prepared **BMA** and after six reaction cycle.

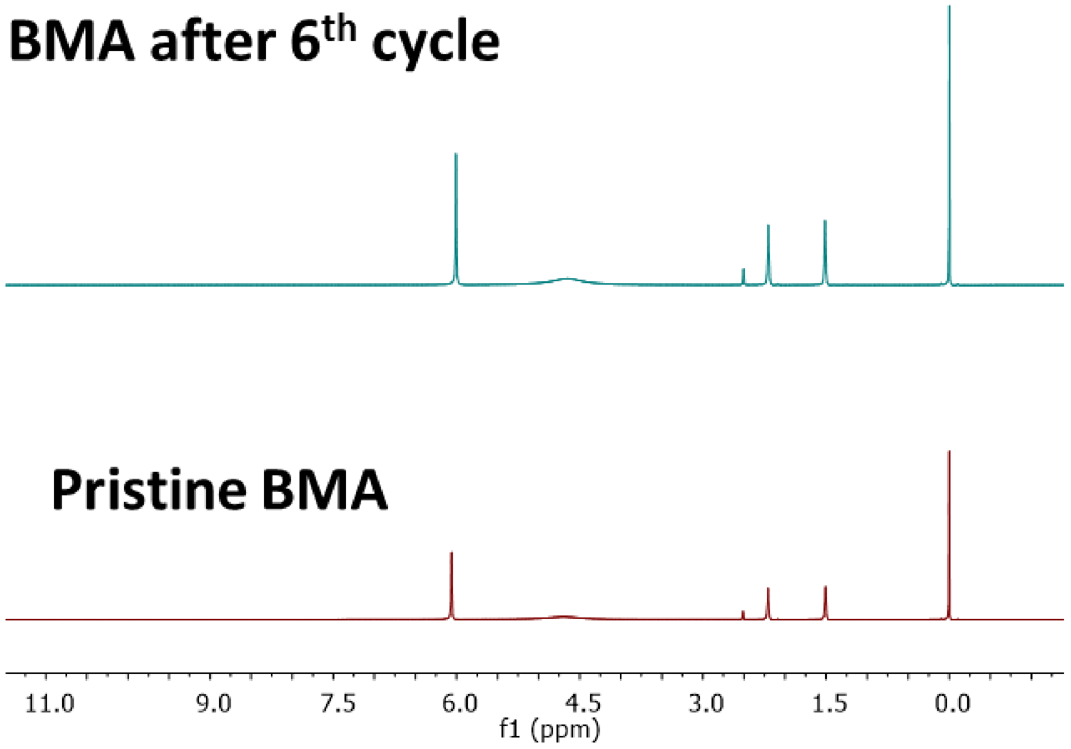

Figure S14. ^{1}H NMR of as-prepared **BMA** and after six reaction cycle in DMSO-d^6.

Table S3. Comparative analysis of reported catalysts for cascade deactalization-Knoevenagel condensation.

Entry	Catalyst	Catalyst amount	Temperature /°C	Solvent	Time/ h	Yield	References
1	1D Zn (II) coordination polymer	3 mol%	MW/ 80	Solvent Free	4	91	[5]
2	Zn-MOF	1 mol%	75	DMF	3	99	[6]
3	Zn(II) metallomacrocycle	1 mol%	80	DMF	3	93	[7]
4	Zirconium MOF (PCM 700)	1 mol%	50	Water	12	99	[8]
5	Amine-grafted H-MFI zeolite	5 wt.%	80	Solvent Free	4	100	[9]
6	Zeolitic porous liquid	10 wt. %	80	Water	3	99	[10]
7	Mesoporous silica with amine and phosphotungstic acid	30 mg	50	-	12	100	[11]
8	Aminopropyl + propyl sulfonic acid, and aminopropyl partly neutralized with phosphotungstic acid	50 mg	90	water	6	100	[12]
9	Proton sponge + propylsulfonic acid	-	80 - 110	water	6	98	[13]
7	Organic polymer (120MI@OH)	5 mg	60	Water	2	99	[14]
8	Amine functionalized GO	50 mg	80	DI water in Toluene	3	95	[15]
9	Core-shell ZSM-5@$Mg_3Si_4O_9(OH)_4$	50 mg	90	Toluene	10	100	[16]
10	MCM-41 with grafted propylamine and propylsulphonic acid	150 mg	80	Toluene	1	96	[17]
11	Melamine adipic acid-based hydrogen bonded system (BMA)	0.017 mol%	Room temperature	Water	1	98	This Work

Hammett correlation study

In six different sealed vials containing water, malononitrile and 5 mg of catalyst, were added para-substituted benzaldehyde dimethyl acetal separately and the reaction mixture was stirred at room temperature for 15 minutes.

Table S4. Substituents parameter values for deacetalization Knoevenagel condensation reaction for Hammett correlation study.

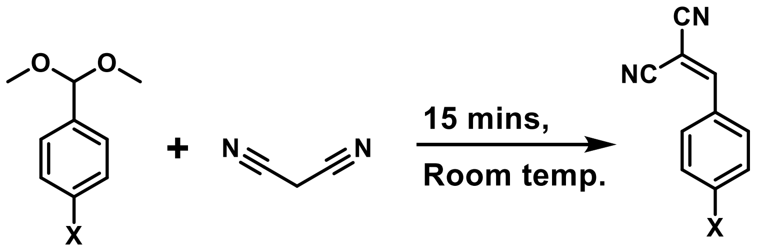

p-substituents	X=H	X=OMe	X=OH	X=Cl	X=CO_2H	X=NO_2
Yield (%)	31	25.5	22.3	37.2	41.3	44.6
K_X/K_H	1	0.822	0.719	1.2	1.33	1.45
σ	0	-0.27	-0.37	0.23	0.45	0.78
Log (K_X/K_H)	0	-0.085	-0.14	0.079	0.123	0.158

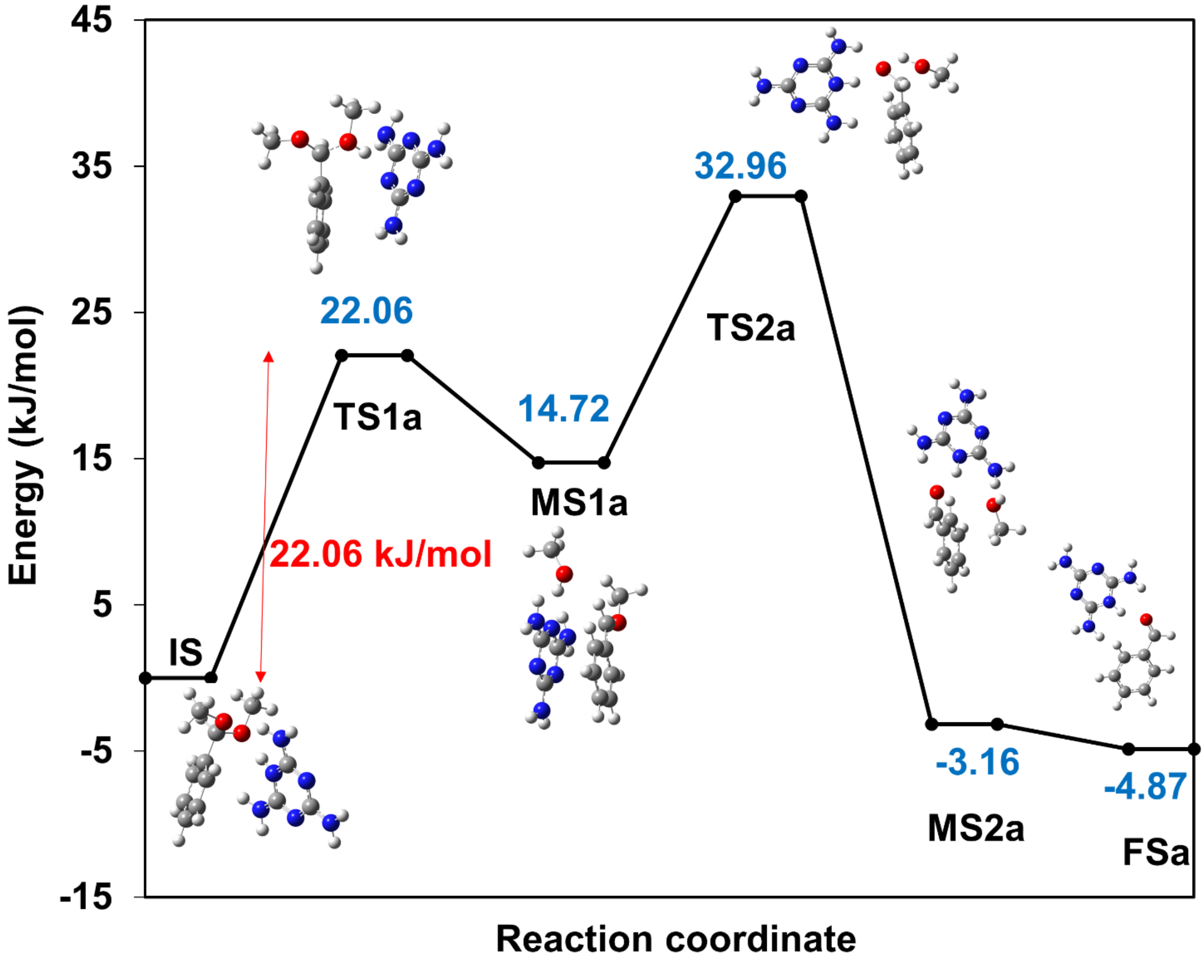

Figure S15. Computed free energy profile for the deacetalisation of benzaldehyde dimethyl acetal over a melaminium cation and associated initial state (IS), metastable intermediate state (MS), transition state (TS) and step 1 product (FSa) structures. Free energy values in kJ.mol^{-1}.

Environmental Factor (E-Factor): E- factor of a chemical reaction is defined as the ratio of the mass of the waste to the mass of the product. For an ideal green chemical reaction the E-factor will be zero.

Environmental Factor= $\frac{mass\ of\ waste}{mass\ of\ the\ product}$

Mass of the waste = total mass of the raw materials- total mass of the products

= 172.22 mg- 151.13 mg – mass of one mole water

= 21.09 mg- 18 mg

Therefore, $E - factor = \frac{mass\ of\ waste}{mass\ of\ the\ product\ (excluding\ water)}$

$= \frac{3.09}{151.13} = 0.0204$

Atom Economy: The efficiency of the chemical reaction is determined from the atom economy value. For an ideal chemical reaction, the value of atom economy should be 100 % as calculated from:

$$Atom\ Economy = \frac{Molecular\ weight\ of\ the\ product}{\sum mol.weight\ of\ stoichiometric\ reactants}\ x\ 100$$

$A.E = \frac{154.14}{172.22}$ x 100 = 89.54 %

Mass Intensity: Mass intensity of a chemical reaction is defined as the ratio of the total mass of reactants involves in the process to the mass of the product. A low value indicates a lower cost and more sustainable process.

$$Mass\ Intensity\ (M.I) = \frac{Total\ mass\ of\ raw\ materials\ in\ process}{Mass\ of\ the\ product}$$

$$M.I = \frac{172.22}{169.13} = 1.01$$

Reaction Mass Efficiency (R.M.E): Reaction mass efficiency is defined as the mass of the product divided by the sum of the total mass of stoichiometric reactants. RME measures the cleanness of the chemical reaction. Values of RME range from 0-100 % with a high value indicating a more sustainable process.

$$RME = \frac{mass\ of\ the\ products}{\sum mass\ \ of\ stoichiometric\ reactants}\ x\ 100$$

$$RME = \frac{169.13}{172.22}\ x\ 100 = 98.20\ \%$$

Process Mass Intensity (PMI): PMI is the ratio between the total mass used in the chemical reaction, including solvents and the mass of the products

$$P.M.I = \frac{Total\ mass\ in\ the\ process\ (including\ solvents)}{mass\ of\ the\ products}$$

$$P.M.I = \frac{172.22 + 3\ mL\ water}{169.13} = 1.337$$

$$P.M.I = \frac{218.21 + 3\ mL\ water}{149.55} = 1.82$$

Table S5. Summary of green metrices parameters calculated for deacetelization-Knoevenagel condensation and Knoevenagel condensation reactions.

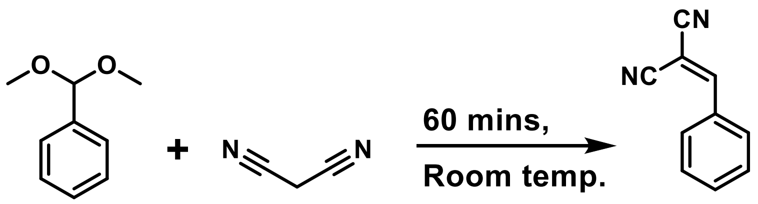

Entry	Metrics	Deacetalization Knoevenagel condensation
1	Environmental factor (E-factor)	0.02
2	Atom economy (AE)	100 %
3	Mass intensity (MI)	1
4	Reaction mass efficiency (RME)	69.23 %
5	Process mass intensity (PMI)	1.24

Substrate Scope

Different substituted acetal were treated with the malononitrile and moderate to excellent yield of the products were obtained irrespective of the substituents. The products were isolated as a pale yellow compounds. The elemental composition was performed using CHNS analysis. Mass of the product was calculated using HRMS(m/z) and GC-MS The sample was prepared in dry acetone. GCMS of the product was performed in the temperature range of -20.0 C to 320 C using SH-I-624Sil MS column of 30 m length, 0.25mm diameter. The obtained products were characterized in JEOL 500Hz NMR spectroscopy using DMSO-d_6 as solvent and tetra methyl silane as a standard reference.

Table S6. Spectral and elemental data of molecular substrates.

Compounds	^{1}H NMR	^{13}C NMR	m/z	CHNS	Reference for NMR
2-benzyledenemalononitrile	δ ppm 8.56 s (1H), 7.955 d (2H), 7.71 t (1H), 7.6 t (2H)	^{13}C NMR: 161.45, 134.17, 131.16, 130.24, 129.42, 113.95, 113.10, 81.41	154.05	C% (77.85), H% (4.02), N% (18.13)	DOI: 10.1016/j.molcata.2010.11.039<br>DOI: 10.1016/j.heliyon.2020.e05293
4-(2,2-dicyanovinyl) benzoic acid	10.10 s (1H), 8.63 s(1H), 8.12 d (2H), 8.0 d (2H)	165.56, 160.00, 134.87, 134.28, 129.57, 128.61, 113.16, 112.40, 83.42	198.15	C% (66.62), H% (3.08), N% (14.10)	
2-(4-Hydroxybenzylidene) malononitrile	9.75 s, 8.27 s (1H), 7.8 d(2H), 6.9 d (2H)	164.90, 160.38, 134.17, 122.97, 116.66, 115.40, 114.49, 75.21	170.15	C% (70.58), H% (3.43), N% (16.45)	
2-(4-Nitrobenzylidene) malononitrile	8.71 s(1H), 8.4 d (2H), 8.1 s (2H)	159.18, 149.99, 136.64, 131.64, 124.30, 114.10, 112.72, 86.02	199.17	C% (60.28), H% (2.52), N% (21.09)	
4-(2,2-Dicyanovinyl) phenyl) boronic acid	8.54 s (1H), 8.40 s (2H), 7.96 d(2H), 7.85 d (2H)	161.46, 134.61, 132.17, 129.66, 128.60, 114.21, 112.75, 81.37	$[198+Na]^{+}$	C% (60.62), H% (3.58), N% (14.06)	
2-(4-Methoxybenzylidene) malononitrile	8.37 s (1H), 7.95 d (2H), 7.17 d (2H), 3.87 s (3H)	164.23, 159.96, 133.24, 123.98, 115.06, 114.67, 113.77, 76.70, 55.61	184.2	C% (71.78), H% (4.32), N% (15.18)	
2-(Thiophen-2-yl-methylene) malononitrile	8.52 s (1H), 8.16 d (1H), 7.87 d (1H), 7.32 t (1H)	154.18, 141.20, 139.34, 135.83, 130.09, 114.91, 114.44, 76.67	160.01	C% (60.18), H% (2.50), N% (17.42), S% (19.90)	
2-(4-(Methylthio)benzylidene) malononitrile	8.42 s (1H), 7.87 d (2H), 7.48 d (2H), 2.56 s (3H)	160.01, 147.57, 130.29, 126.81, 124.99, 113.90, 112.83, 78.04, 13.19	$[200.04+Na]^{+}$	: C% (65.94), H% (4.11), N% (14.07), S% (15.88)	
2-((2-Hydroxy-napthalen-1-yl) methylene) malononitrile	10.73 s (1H), 8.83 s (1H), 8.08 d (1H), 7.8 t (1H), 7.5~7.4 m (2H), 7.18 d (1H)	193.00, 173.21, 164.81, 138.78, 133.71, 131.90, 129.55, 129.01, 127.51, 122.76, 121.94, 119.6, 112.38, 70.48	$[220+H]^{+}$	C% (76.53), H% (3.65), N% (12.58)	
2-((5-(Hydroxymethyl) furan-2-yl) methylene) malononitrile	8.22 s (1H), 7.4 d (1H), 6.73 d (1H), 5.66 s (1H), 4.53 s (2H)	164.86, 147.50, 144.20, 127.11, 114.52, 113.38, 111.85, 73.61, 56.16	174.04	C% (62.07), H% (3.39), N% (16.14)	
2-(4-Chlorobenzylidene) malononitrile	8.54 s (1H), 7.95 d (2H), 7.71 d (2H)	160.15, 139.08, 132.44, 129.94, 129.76, 114.09, 112.89, 82.28	$[188.01+Li]^{+}$	C% (63.58), H% (2.66), N% (14.92)	
2-((5-Bromo thiophen-2-yl) methylene malononitrile	8.66 s (1H), 7.7 d (1H), 7.5 d (1H)	152.37, 141.39, 136.86, 132.87, 125.33, 114.29, 113.78, 76.96	$[237.92+Na]^{+}$	C% (40.18), H% (1.23), N% (12.05), S% (13.38)	
2-(4-Bromobenzylidene) malononitrile	8.54 s (1H), 7.87 d (4H)	160.50, 132.47, 132.15, 130.11, 128.27, 114.02, 113.04, 82.34	$[235+Li]^{+}$	C% (51.54), H% (2.18), N% (12.05)	
4-(Dimethyl amino) benzaldehyde	9.66 s (1H), 7.69, 7.67 d (2H), 6.79, 6.77 d(2H)	189.92, 154.19, 133.47, 118.61, 110.92, 8.29	$[220+Na]^{+}$	C% (73.08), H% (5.68), N% (21.24)	

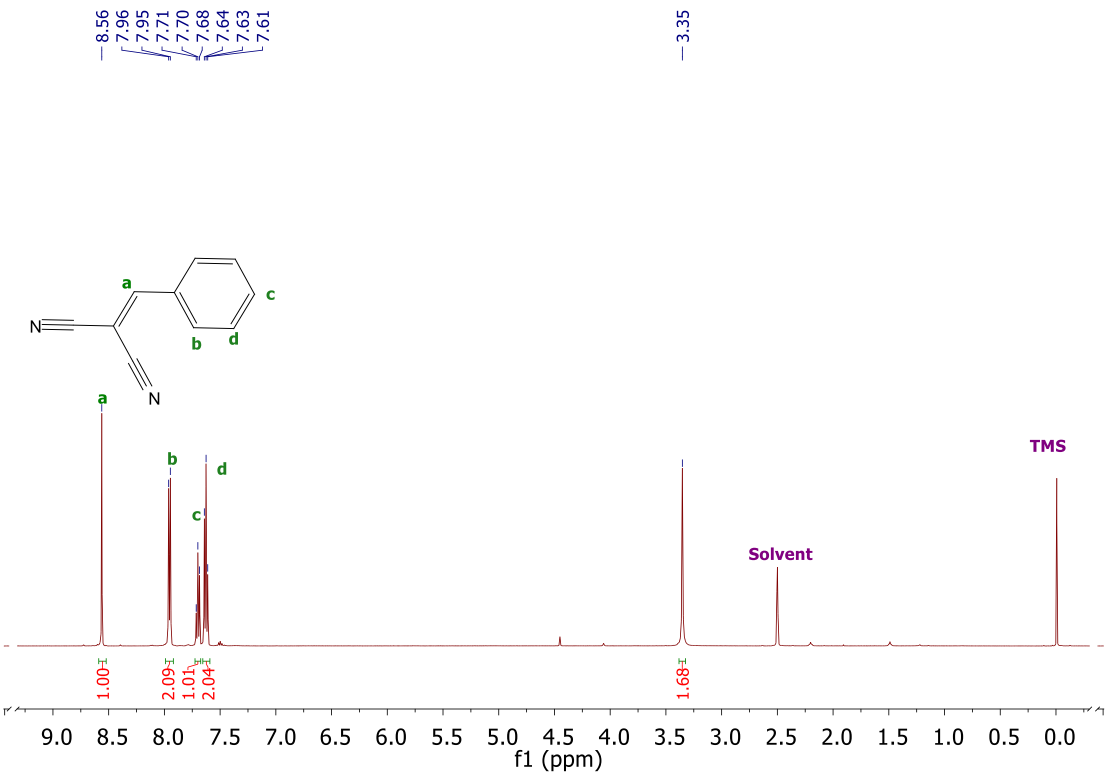

Figure S16. ^{1}H NMR of 2-benzyledenemalononitrile

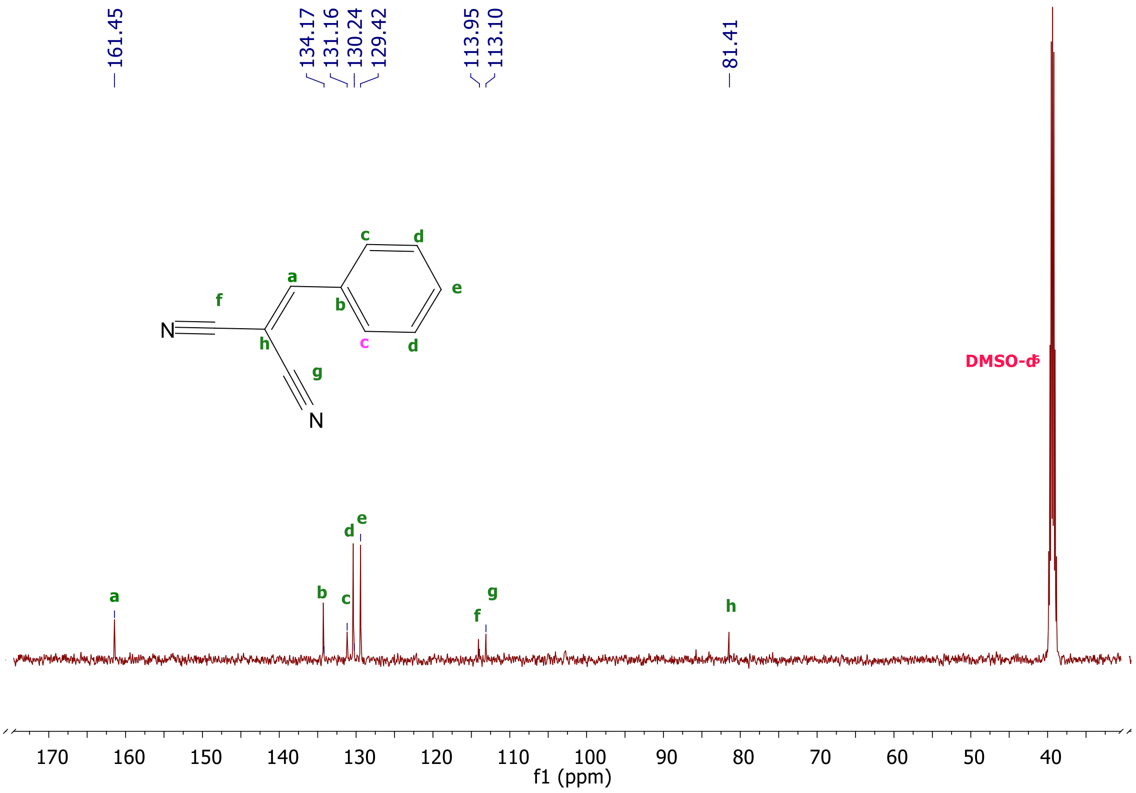

Figure S17. ^{13}C NMR of 2-benzyledenemalononitrile

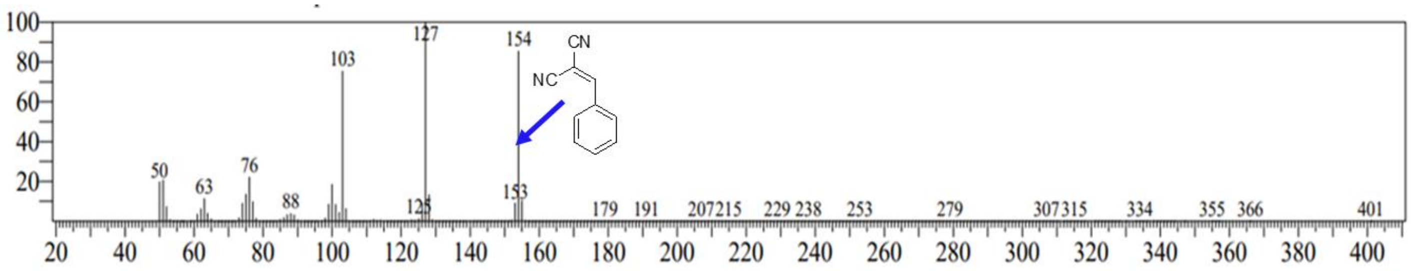

Figure S18. Mass of 2-benzyledenemalononitrile obtained from GCMS

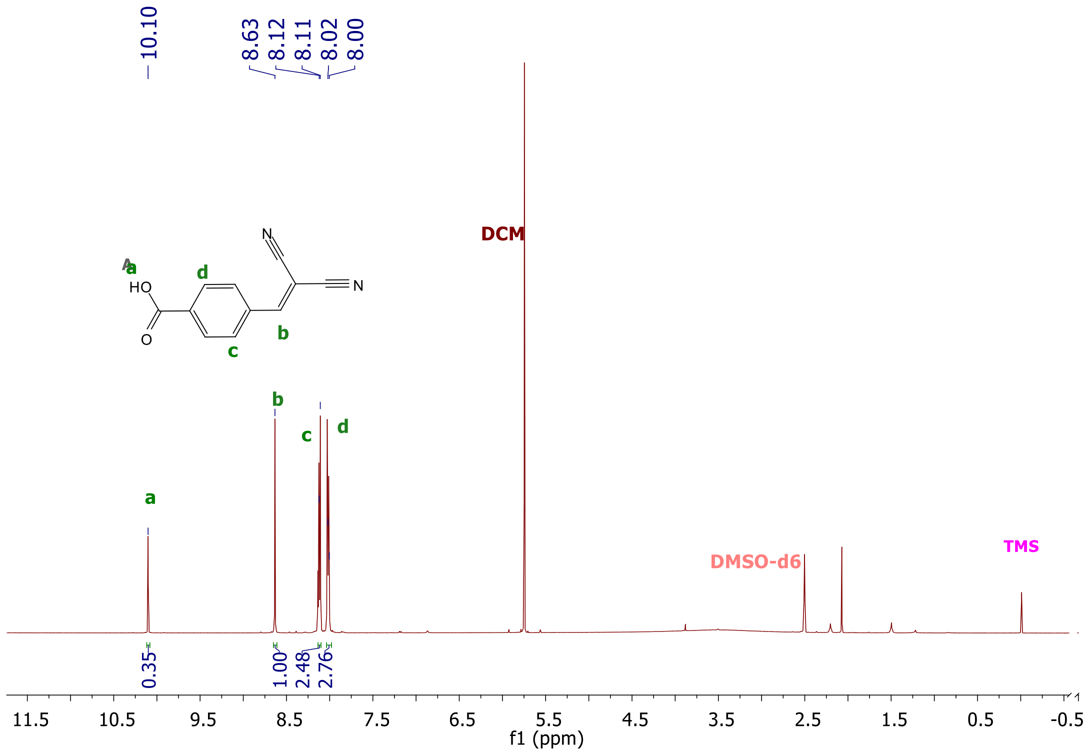

Figure S19. ^{1}H NMR of 4-(2,2-dicyanovinyl) benzoic acid

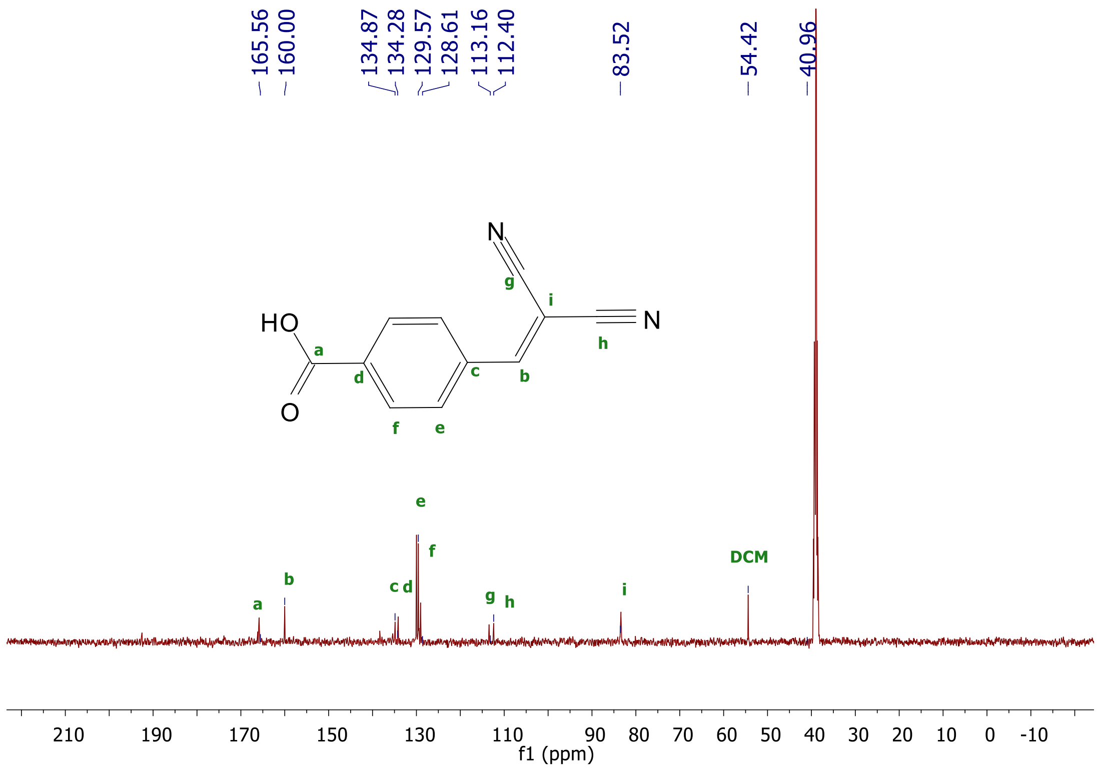

Figure S20. ^{13}C NMR of 4-(2,2-dicyanovinyl) benzoic acid

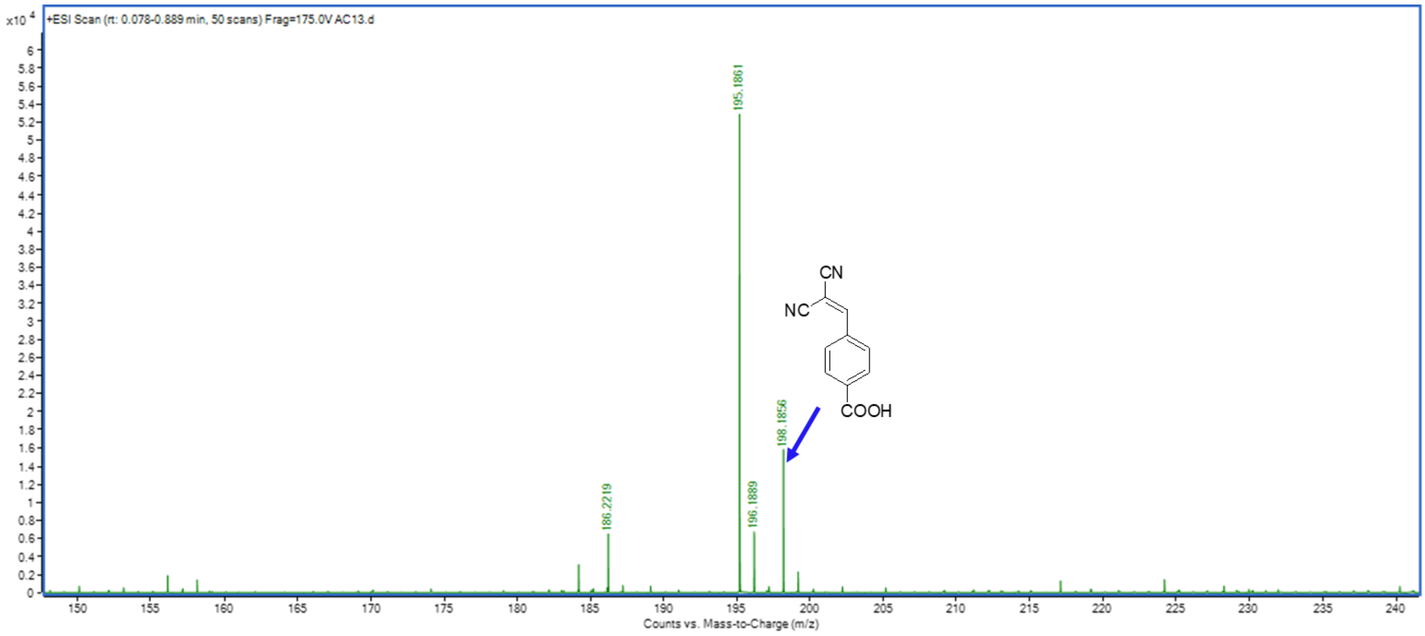

Figure S21. m/z spectra of 4-(2,2-dicyanovinyl) benzoic acid obtained from HRMS

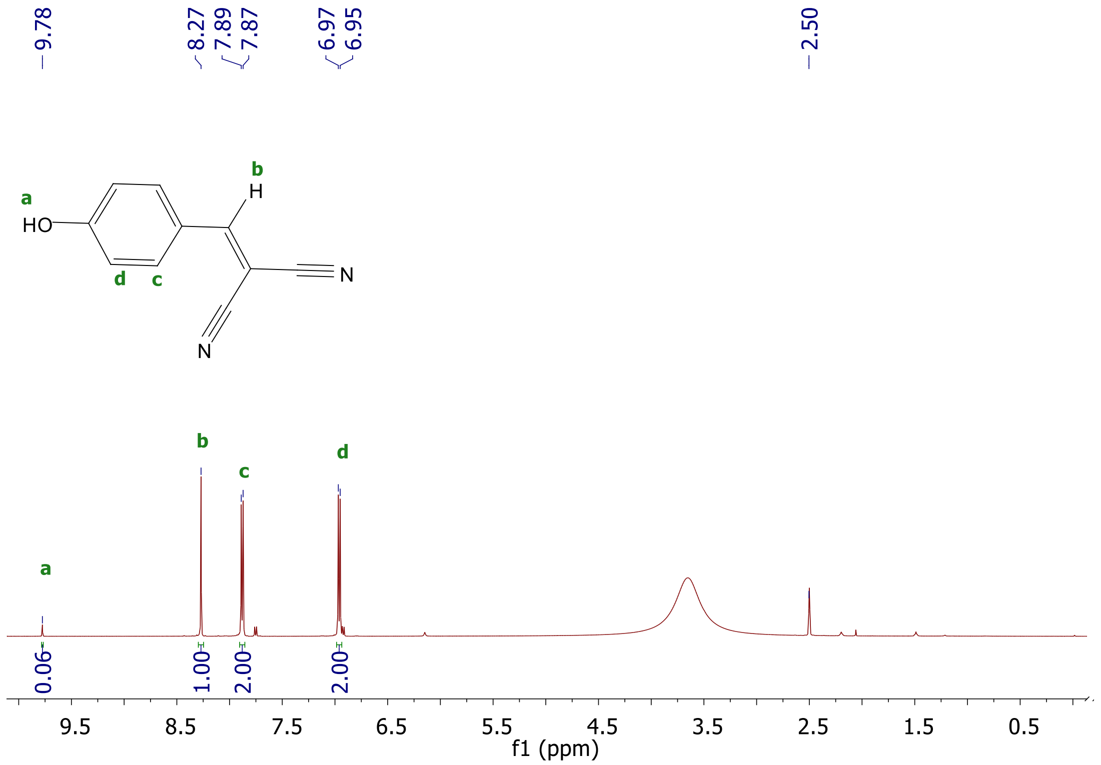

Figure S22. ^{1}H NMR of 2-(4-Hydroxybenzylidene) malononitrile

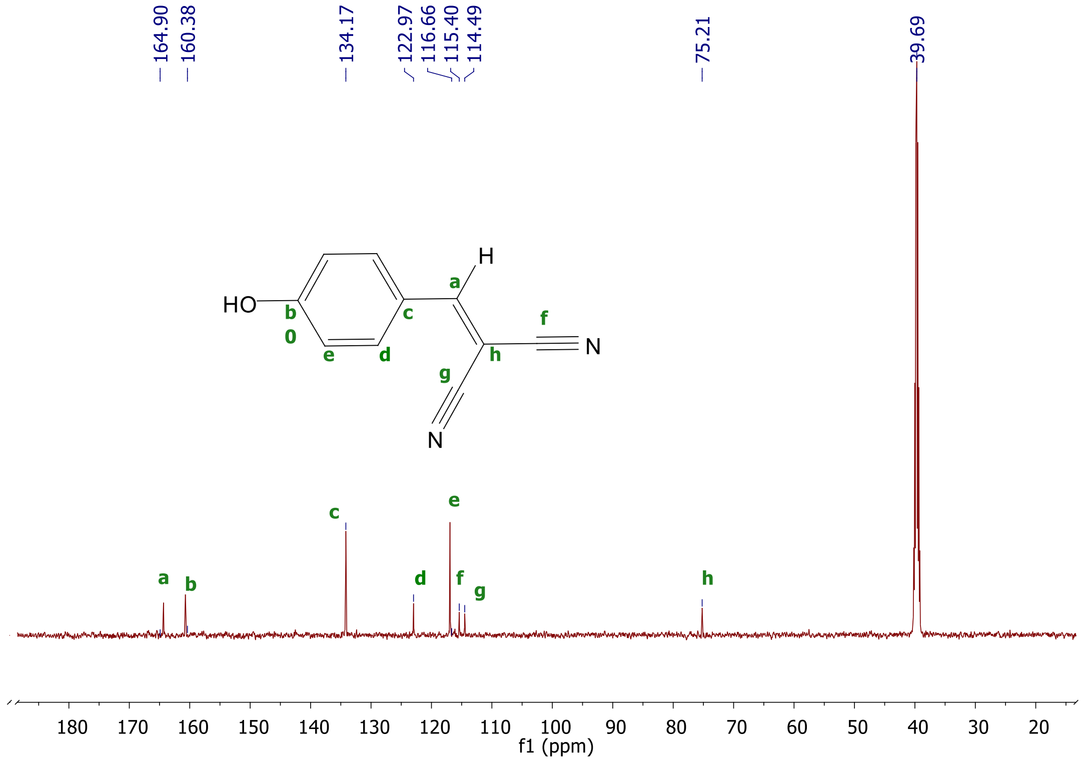

Figure S23. ^{13}C NMR of 2-(4-Hydroxybenzylidene) malononitrile

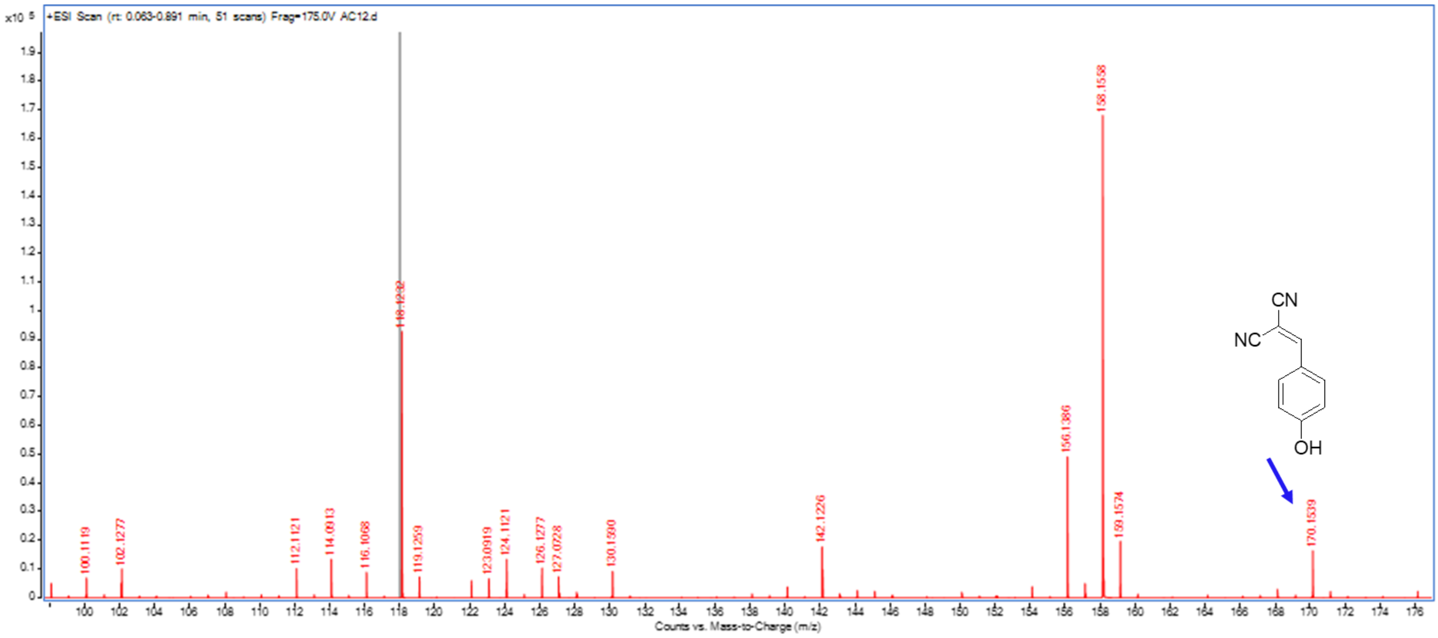

Figure S24. m/z spectra of 2-(4-Hydroxybenzylidene) malononitrile obtained from HRMS

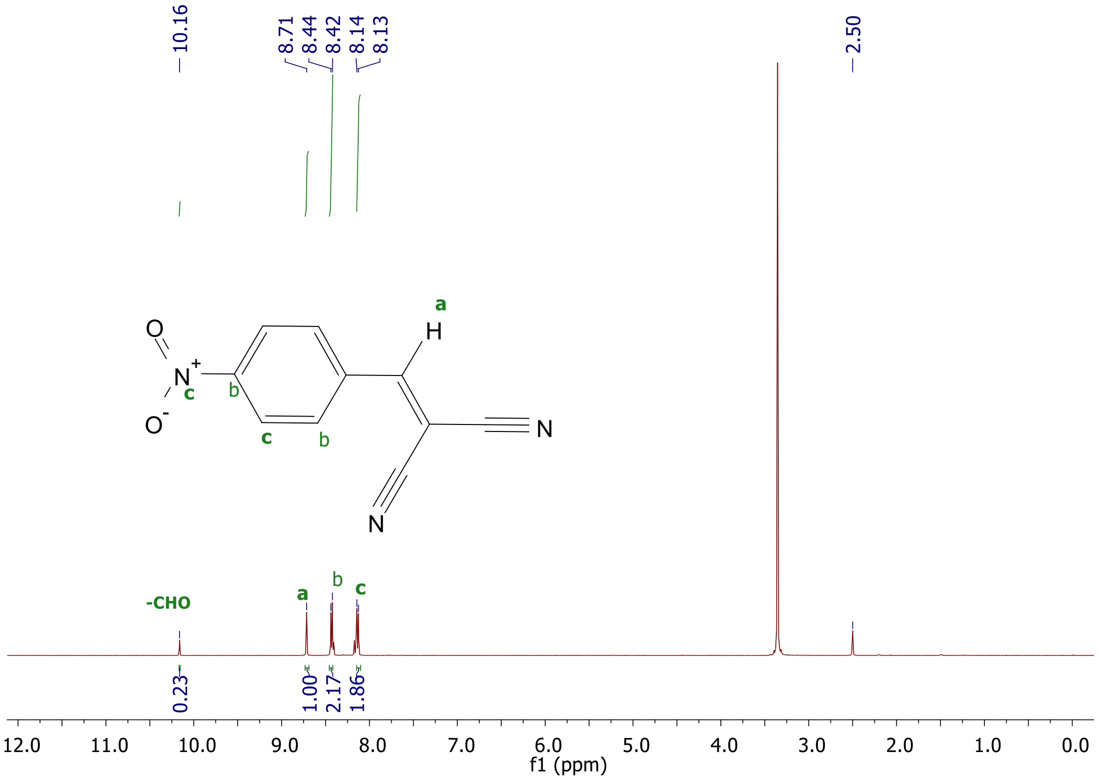

Figure S25. ^{1}H NMR of 2-(4-nitrobenzylidene) malononitrile

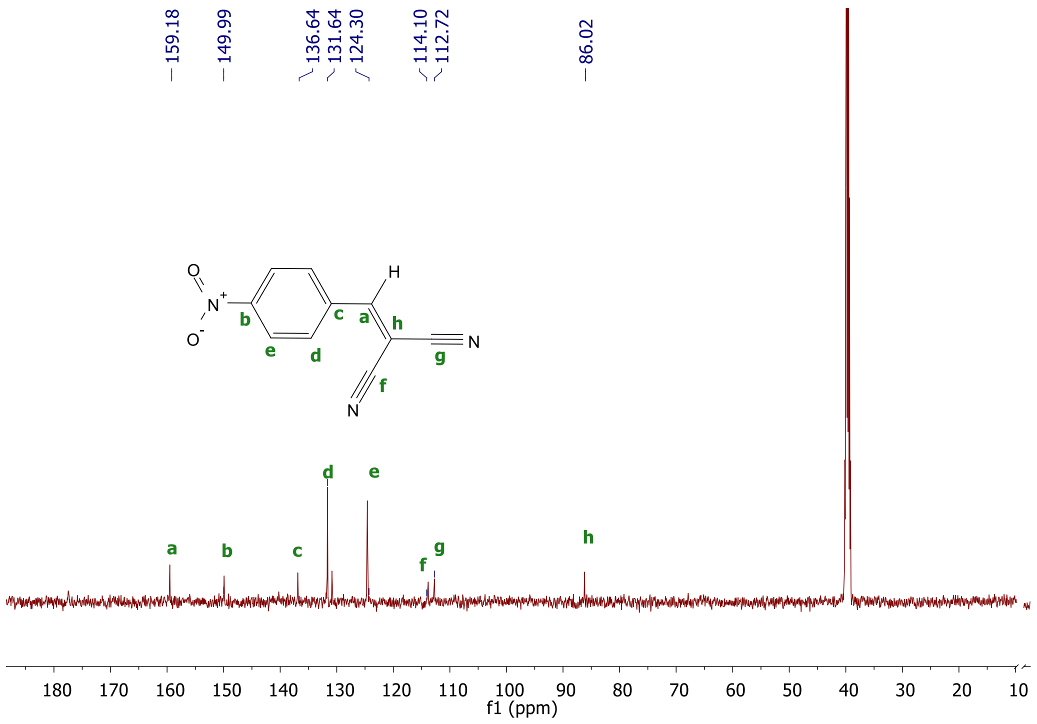

Figure S26. ^{13}C NMR of 2-(4-nitrobenzylidene) malononitrile

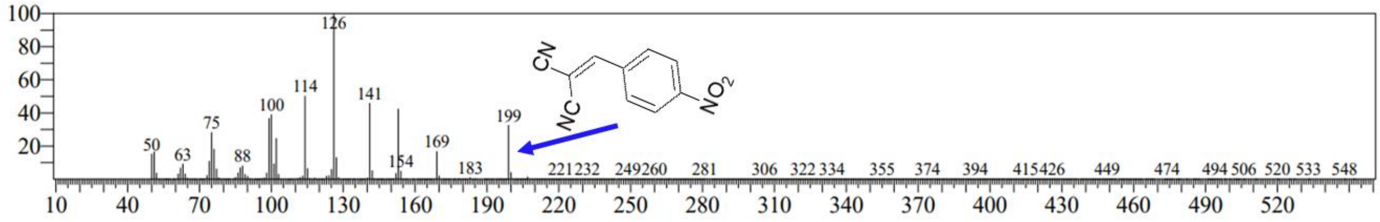

Figure S27. Mass of 2-(4-nitrobenzylidene) malononitrile obtained from GCMS

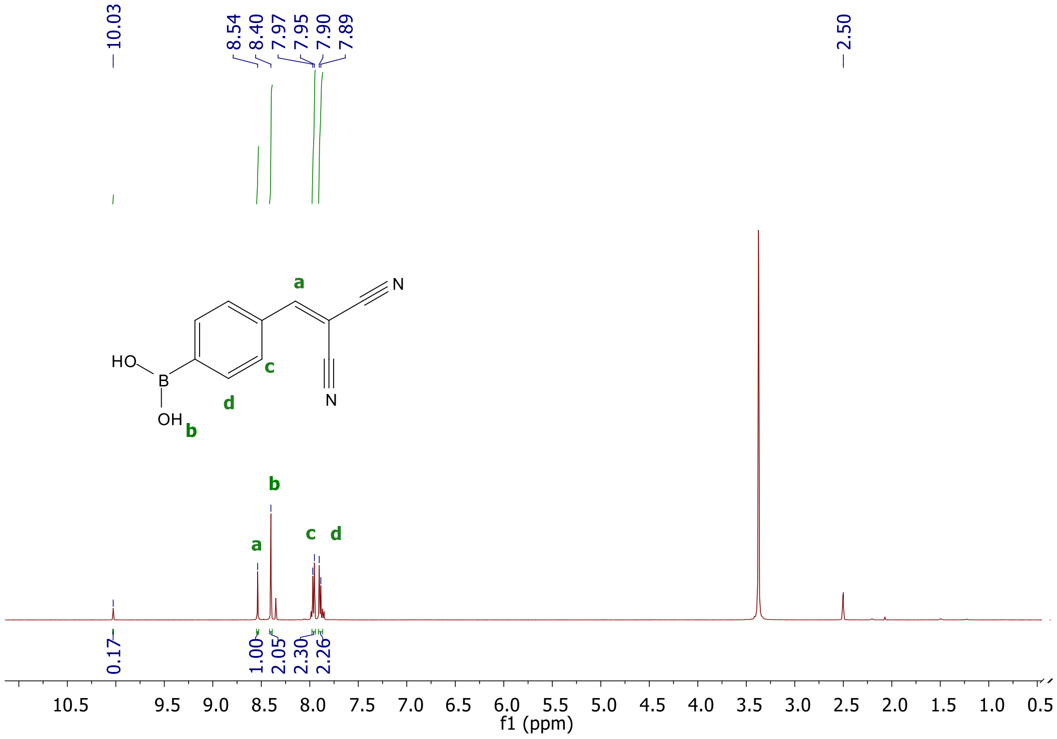

Figure S28. ^{1}H NMR of (4-(2,2-dicyanovinyl) phenyl) boronic acid

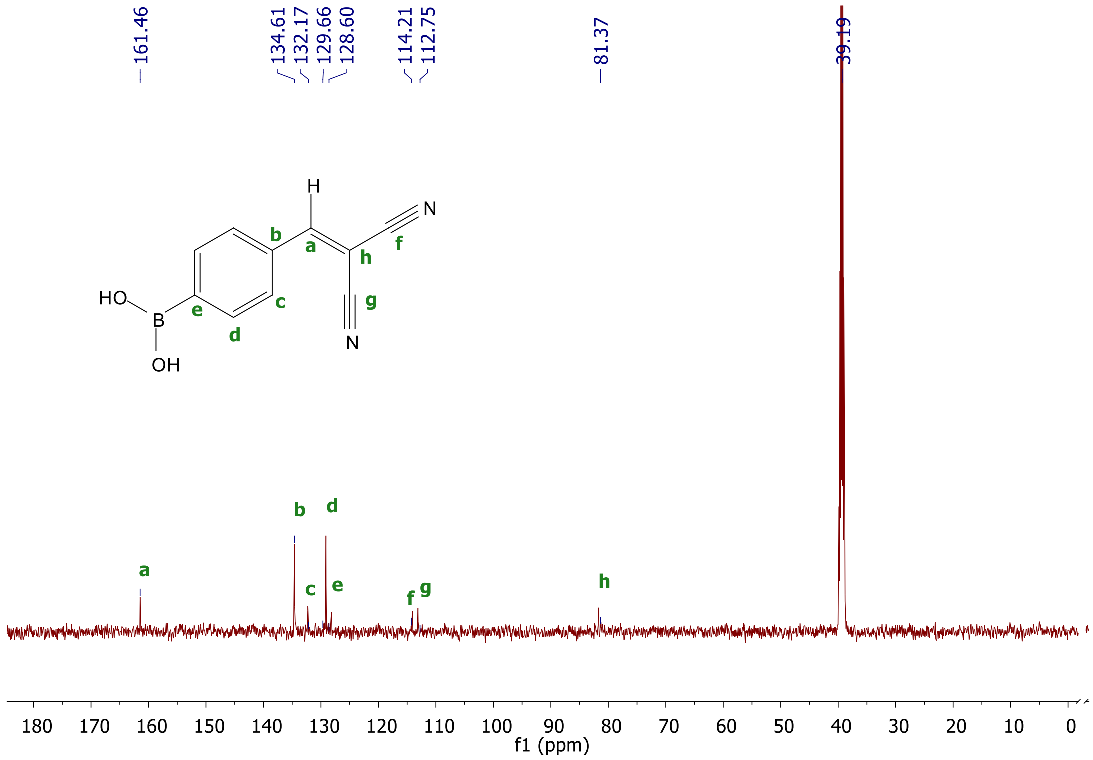

Figure S29. ^{13}C NMR of (4-(2,2-dicyanovinyl) phenyl) boronic acid

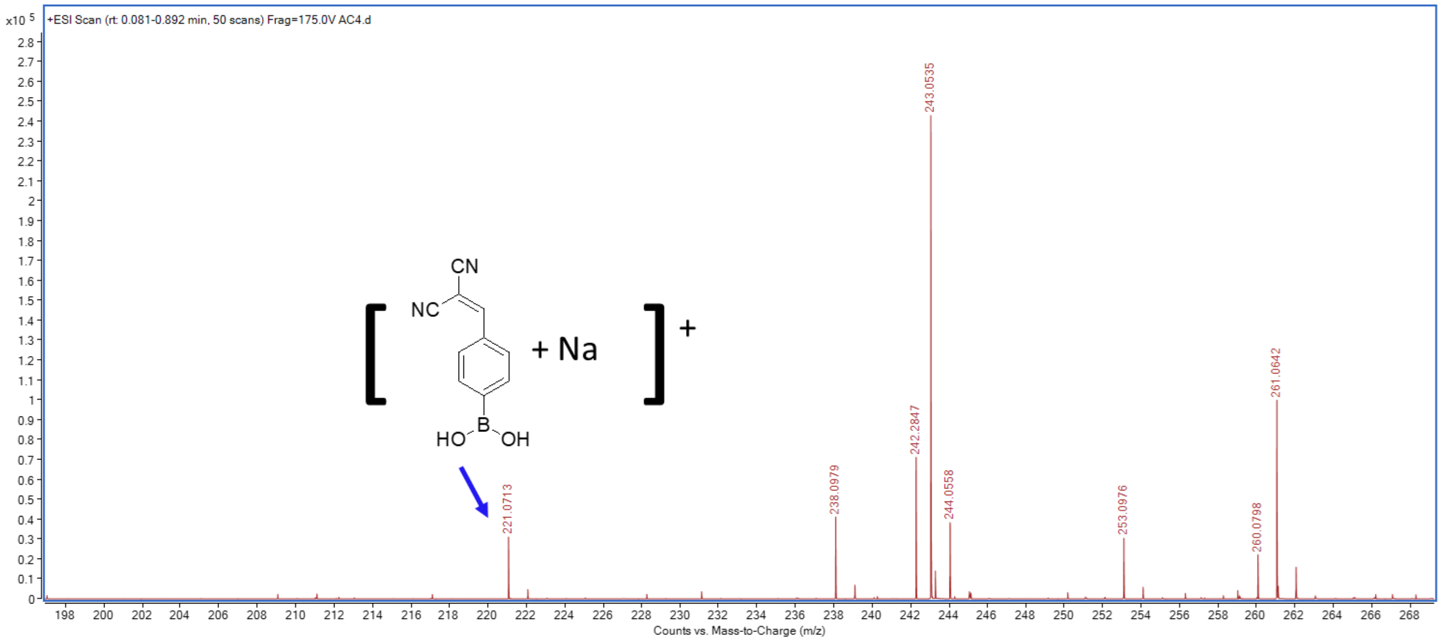

Figure S30. m/z spectra of (4-(2,2-dicyanovinyl) phenyl) boronic acid obtained from HRMS

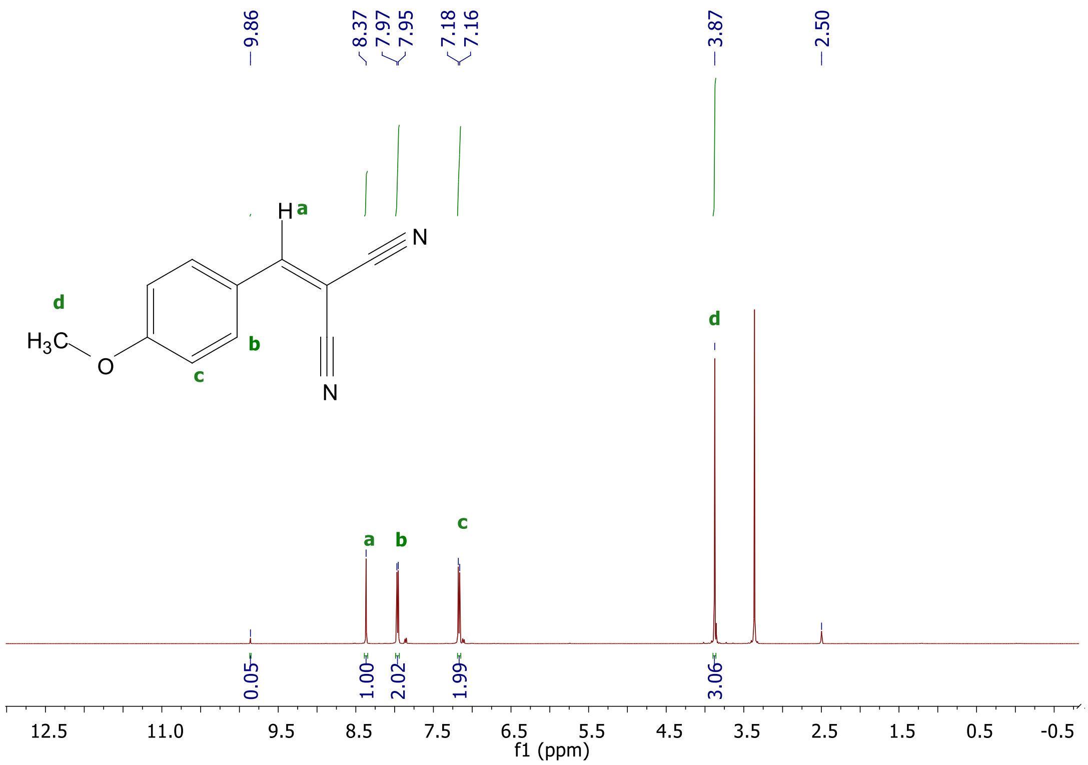

Figure S31. ^{1}H NMR of 2-(4-methoxybenzylidene) malononitrile

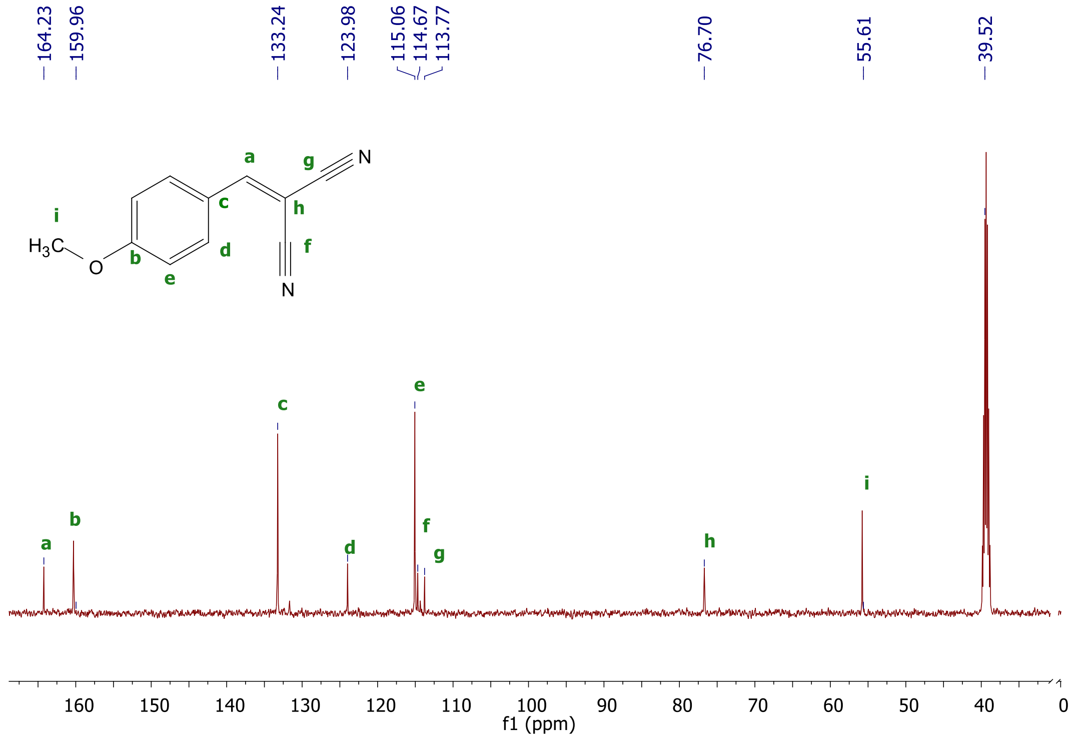

Figure S32. ^{13}C NMR of 2-(4-methoxybenzylidene) malononitrile

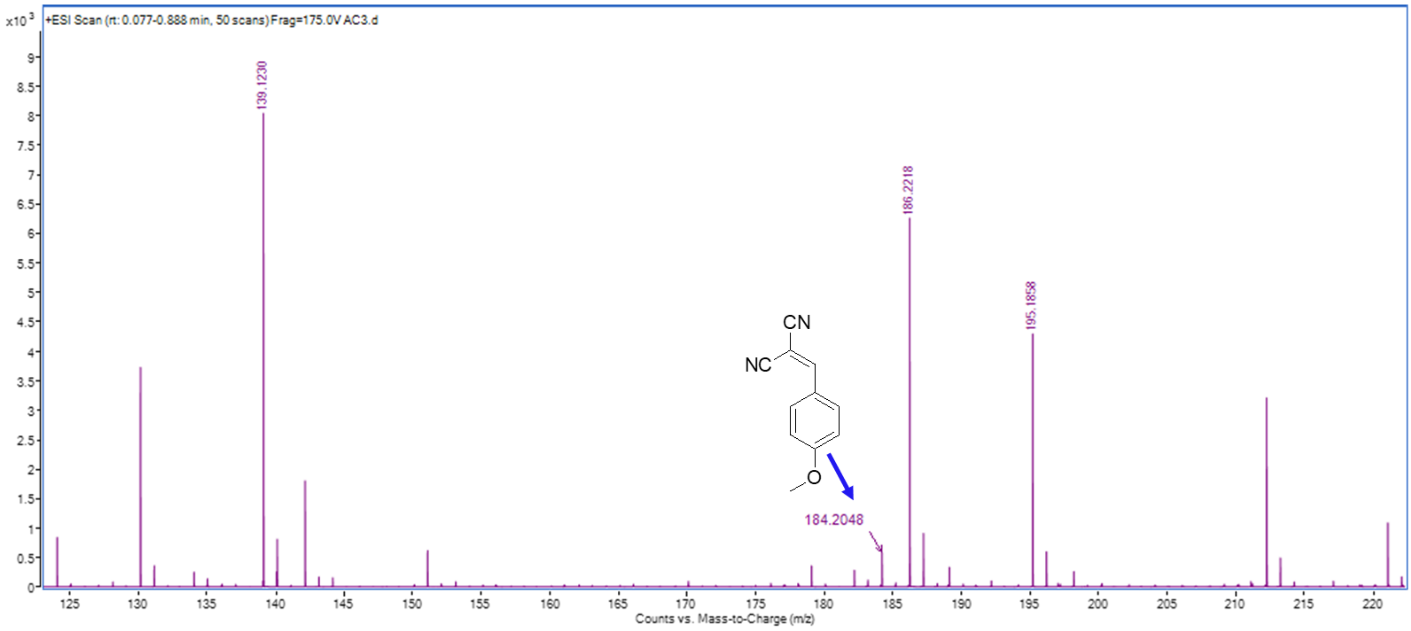

Figure S33. m/z spectra of 2-(4-methoxybenzylidene) malononitrile obtained from HRMS

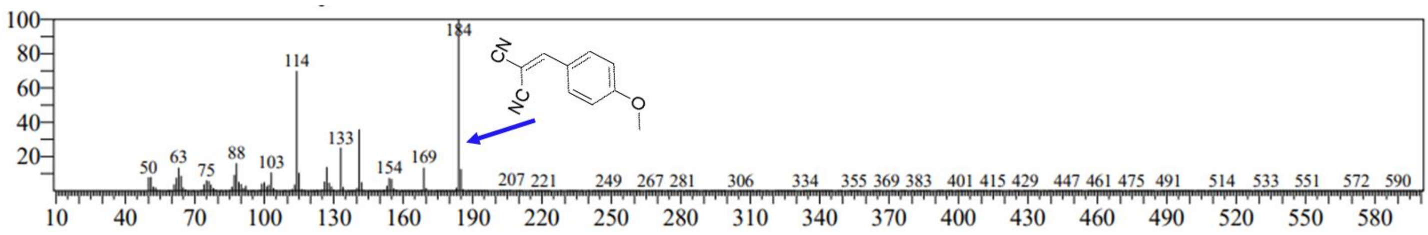

Figure S34. mass of 2-(4-methoxybenzylidene) malononitrile obtained from GCMS

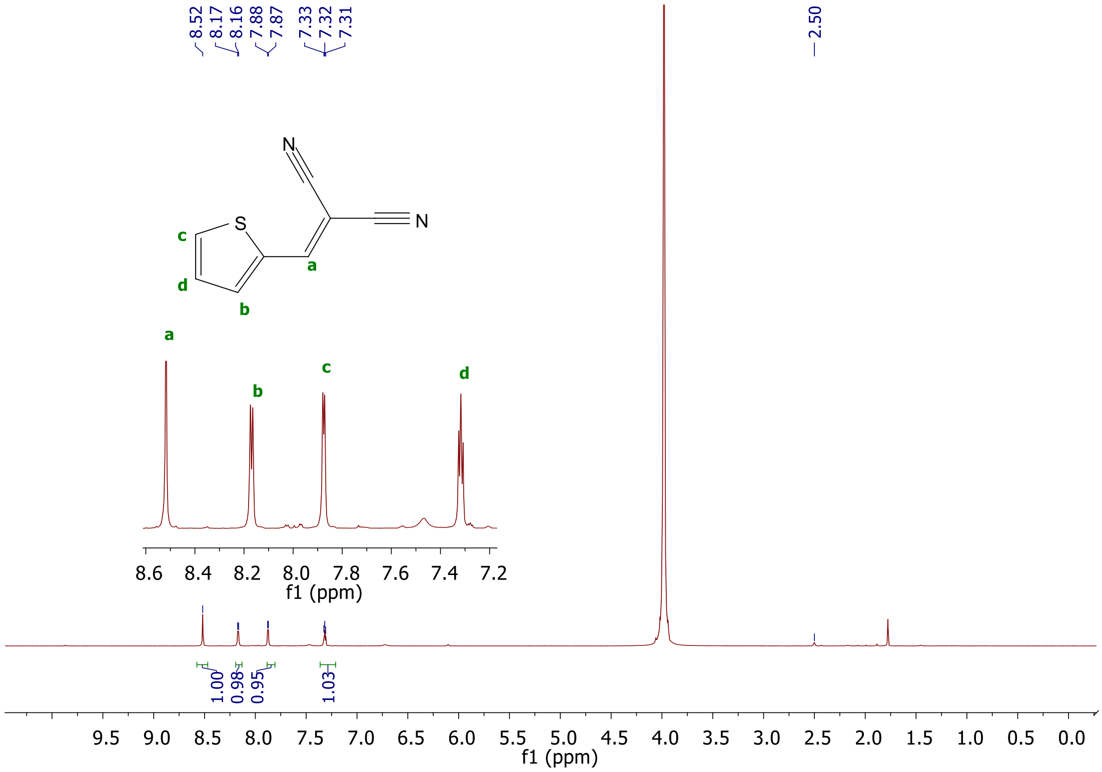

Figure S35. ^{1}H NMR of 2-(thiophen-2-yl-methylene) malononitrile

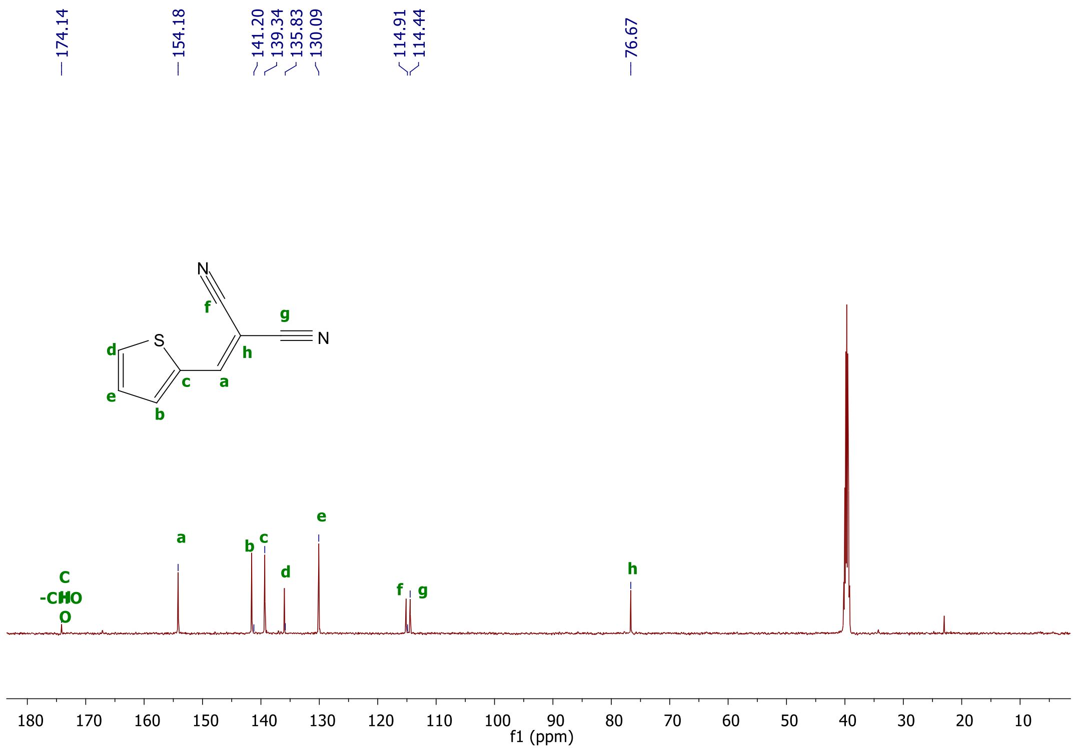

Figure S36. ^{13}C NMR of 2-(thiophen-2-yl-methylene) malononitrile

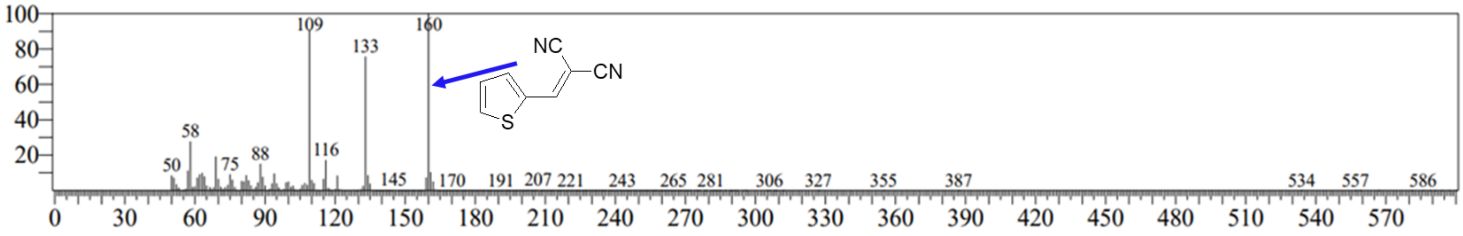

Figure S37. Mass of 2-(thiophen-2-yl-methylene) malononitrile obtained from GCMS

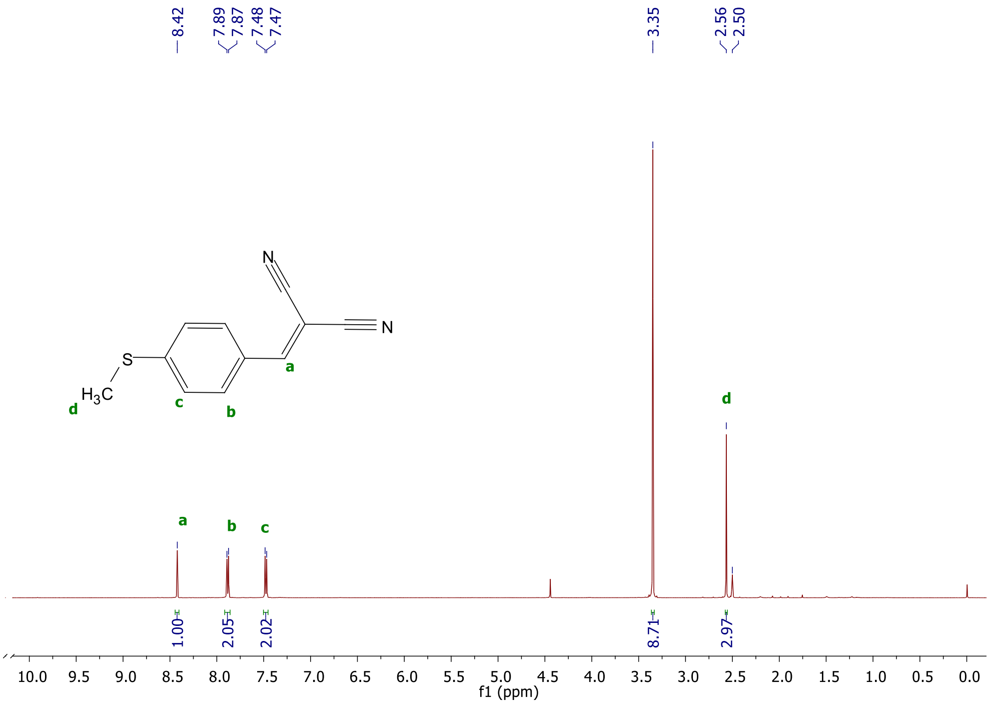

Figure S38. ^{1}H NMR of 2-(4-(methylthio)benzylidene) malononitrile

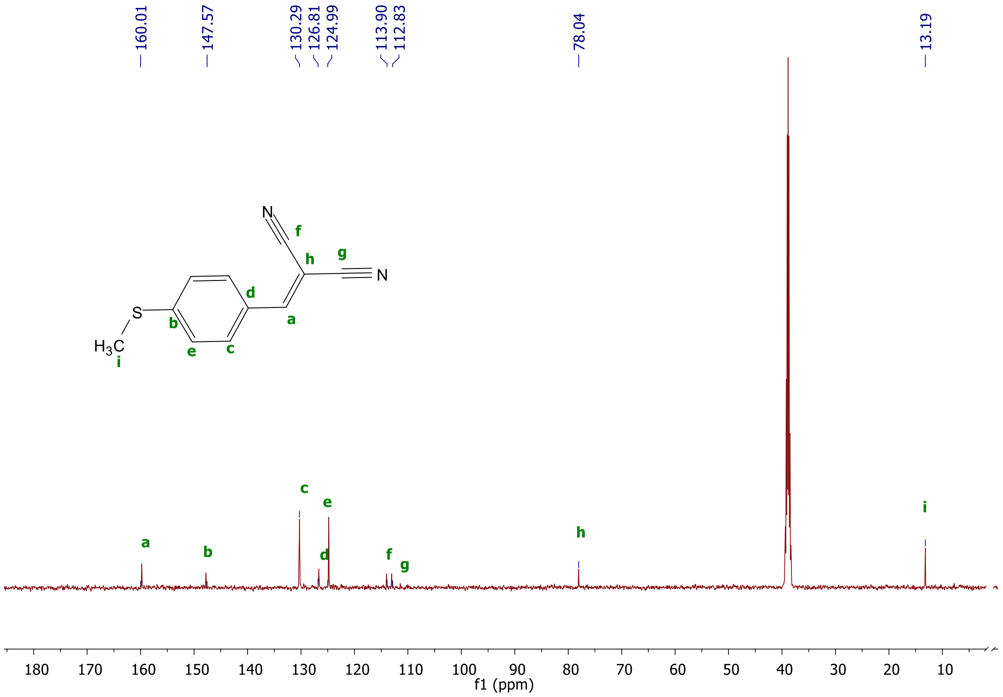

Figure S39. ^{13}C NMR of 2-(4-(methylthio)benzylidene) malononitrile

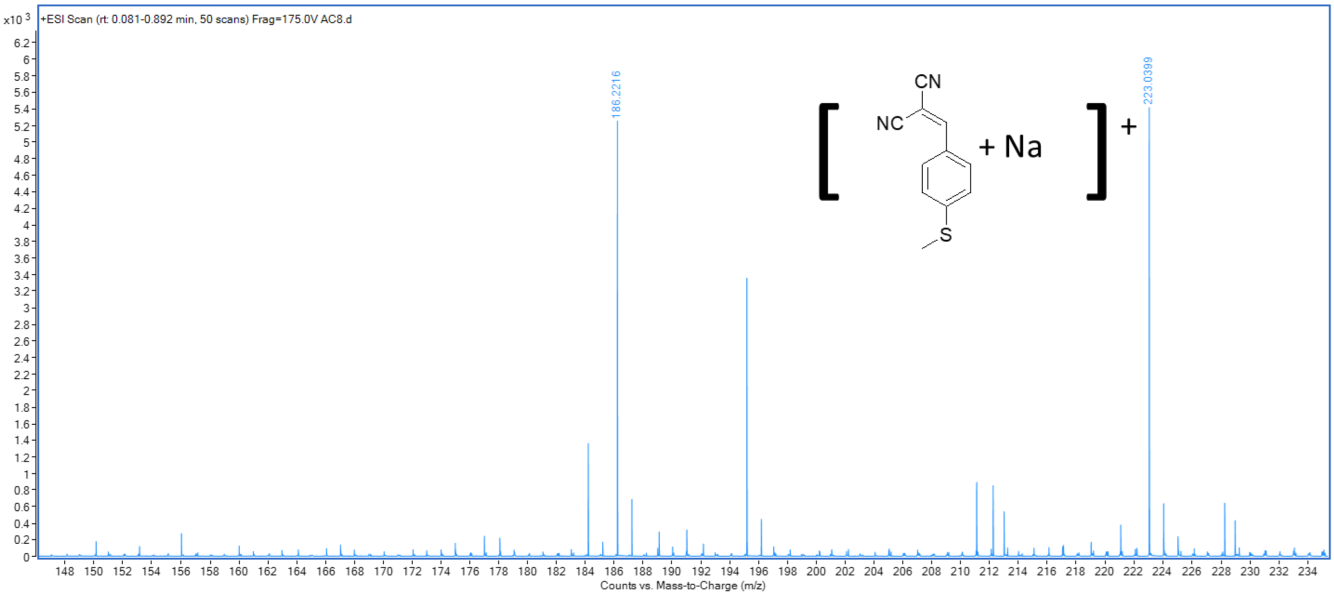

Figure S40. m/z spectra of 2-(4-(methylthio)benzylidene) malononitrile obtained from HRMS

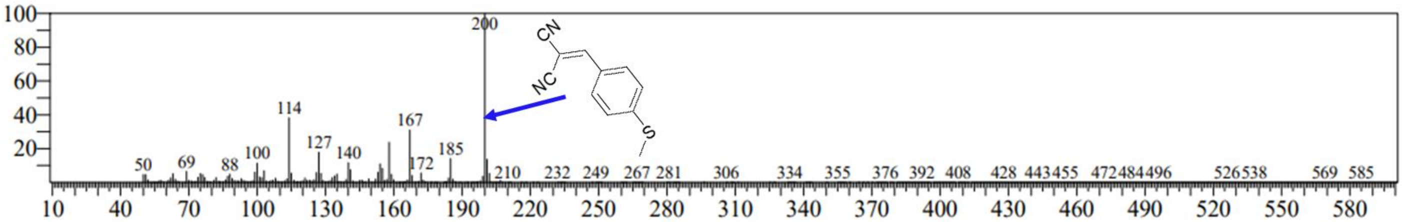

Figure S41. Mass of 2-(4-(methylthio)benzylidene) malononitrile obtained from GCMS

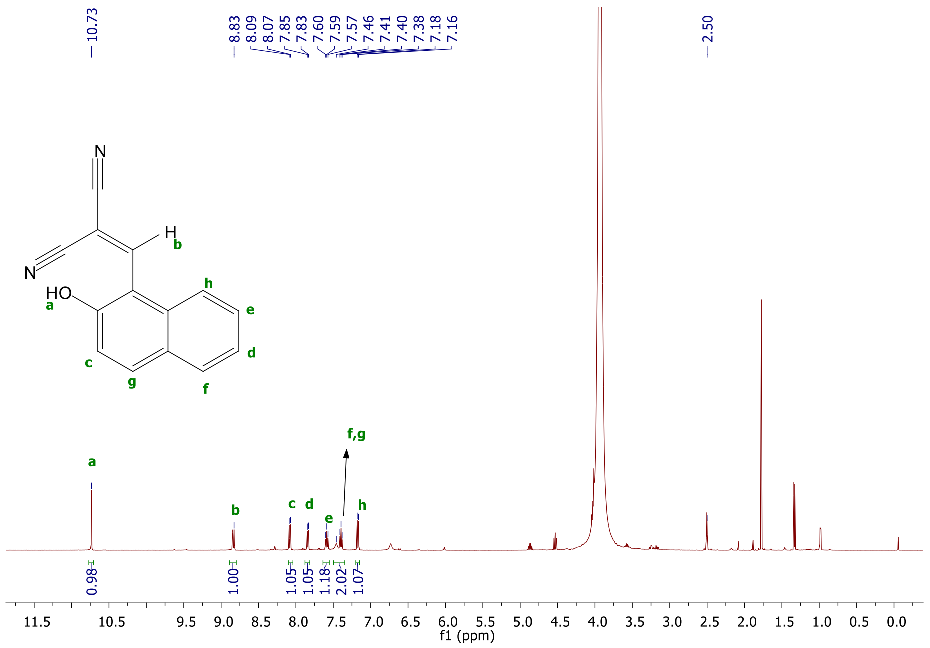

Figure S42. ^{1}H NMR of 2-((2-hydroxy-napthalen-1-yl) methylene) malononitrile

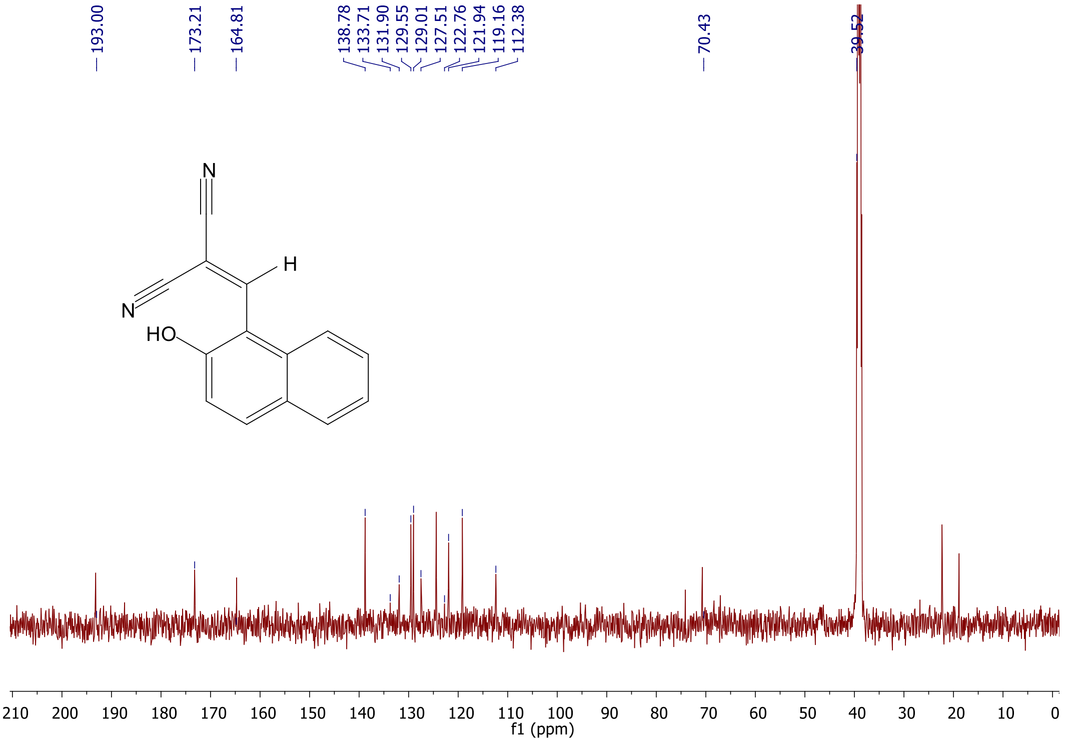

Figure S43. ^{13}C NMR of 2-((2-hydroxy-napthalen-1-yl) methylene) malononitrile

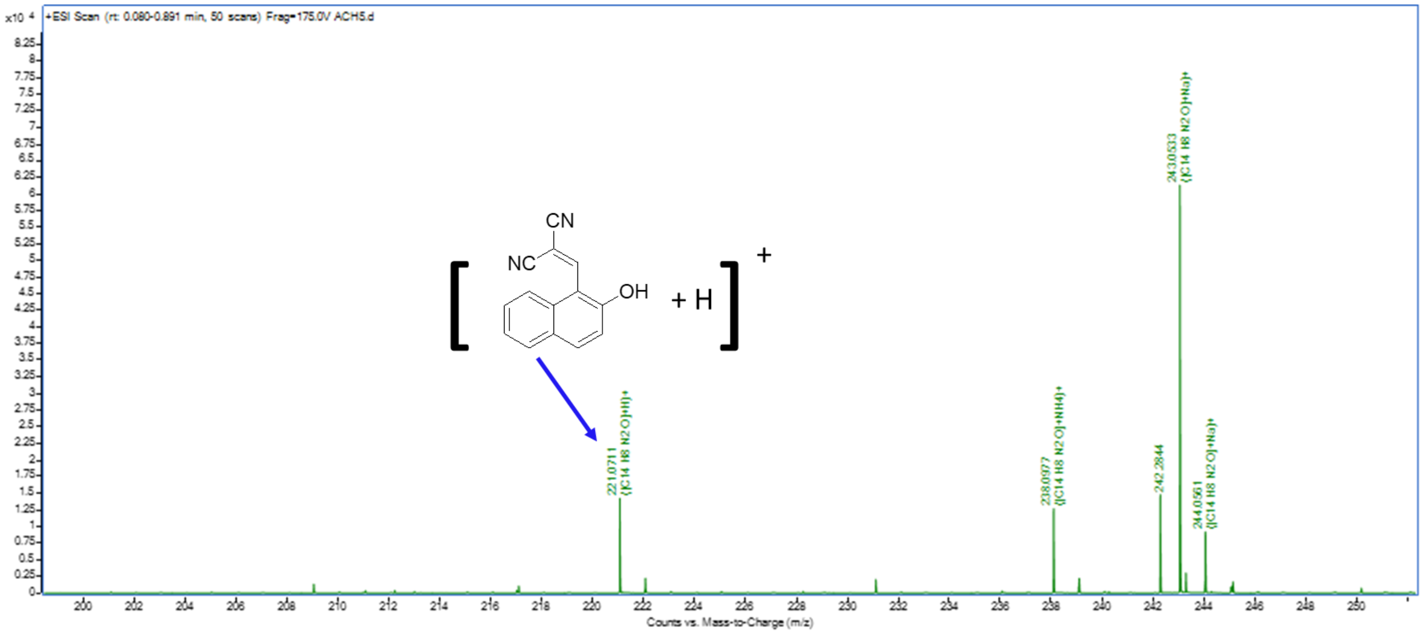

Figure S44. m/z spectra of 2-((2-hydroxy-napthalen-1-yl) methylene) malononitrile obtained from HRMS

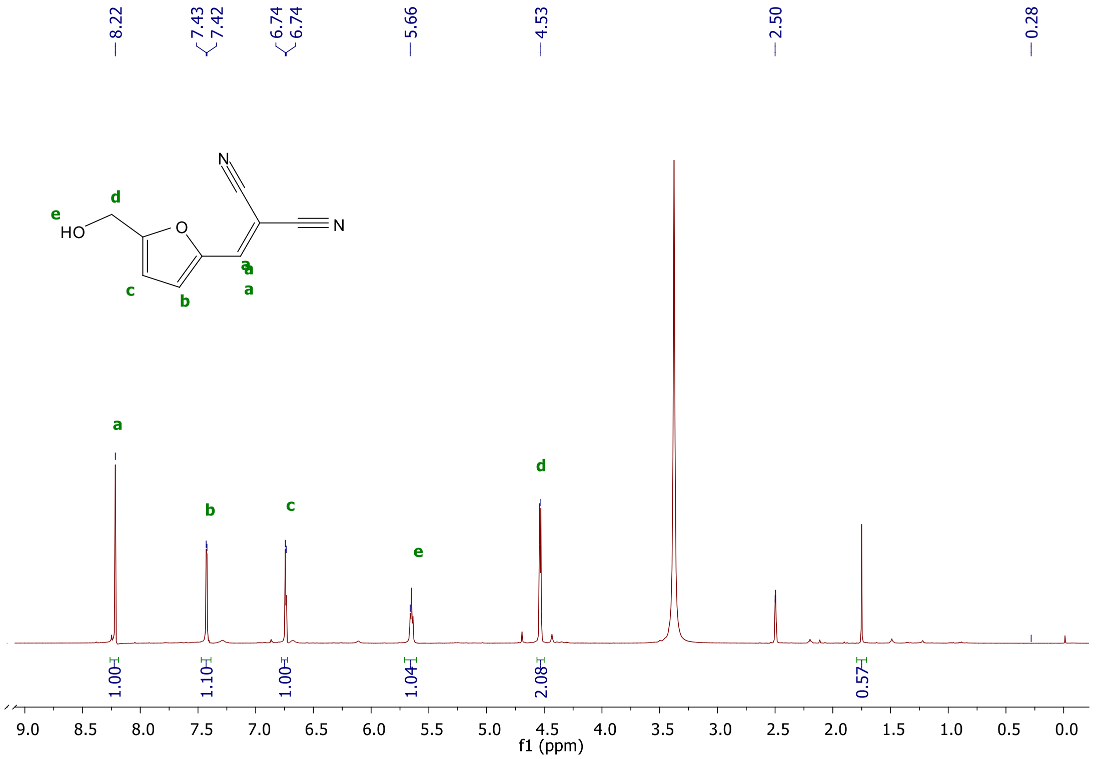

Figure S45. ^{1}H NMR of 2-((5-(hydroxymethyl) furan-2-yl) methylene) malononitrile

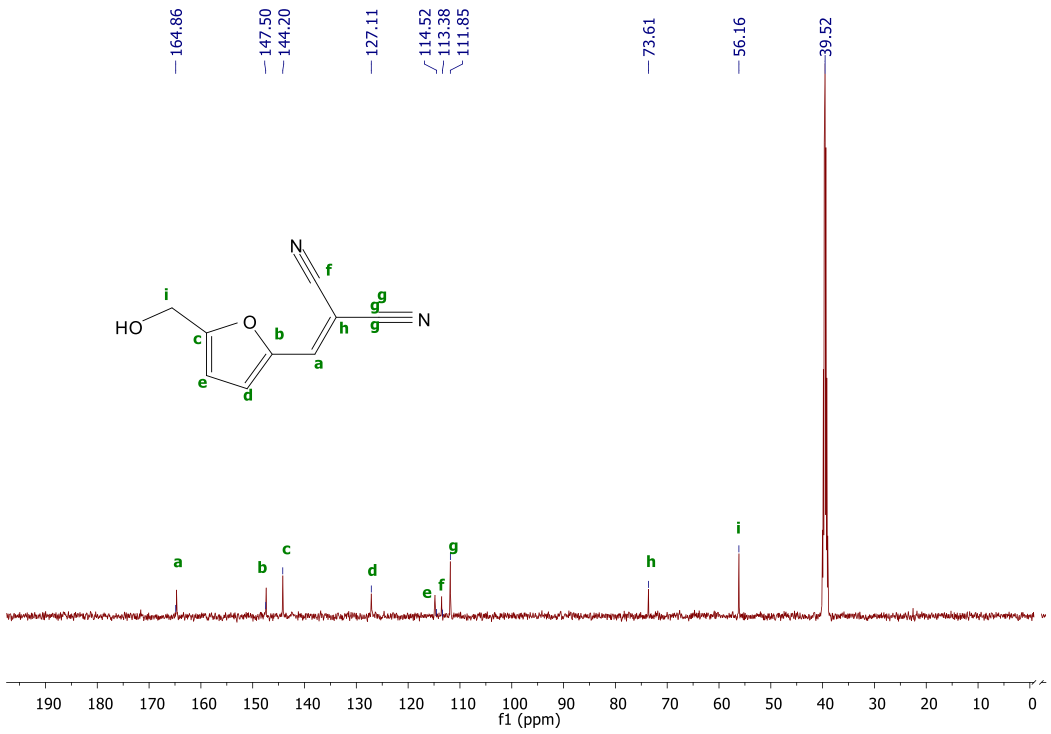

Figure S46. ^{13}C NMR of 2-((5-(hydroxymethyl) furan-2-yl) methylene) malononitrile

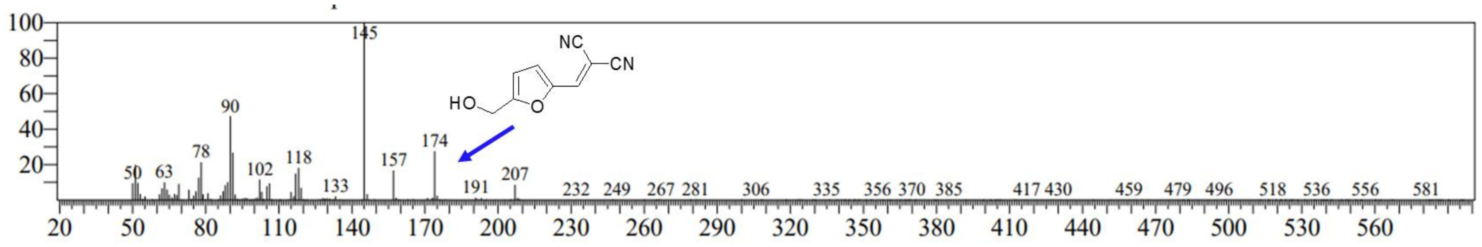

Figure S47. Mass of 2-((5-(hydroxymethyl) furan-2-yl) methylene) obtained from GCMS

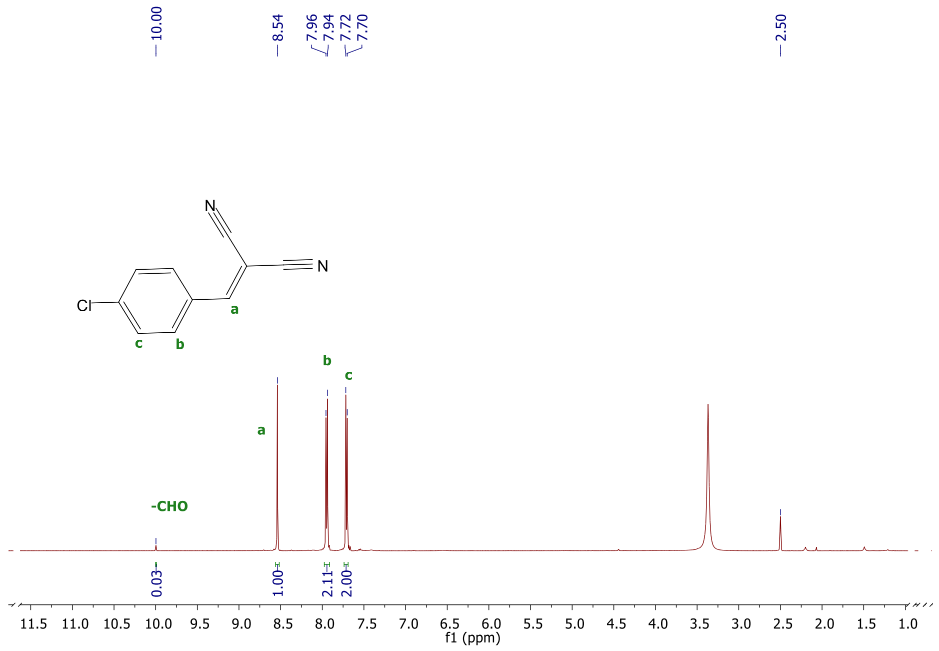

Figure S48. ^{1}H NMR of 2-(4-chlorobenzylidene) malononitrile

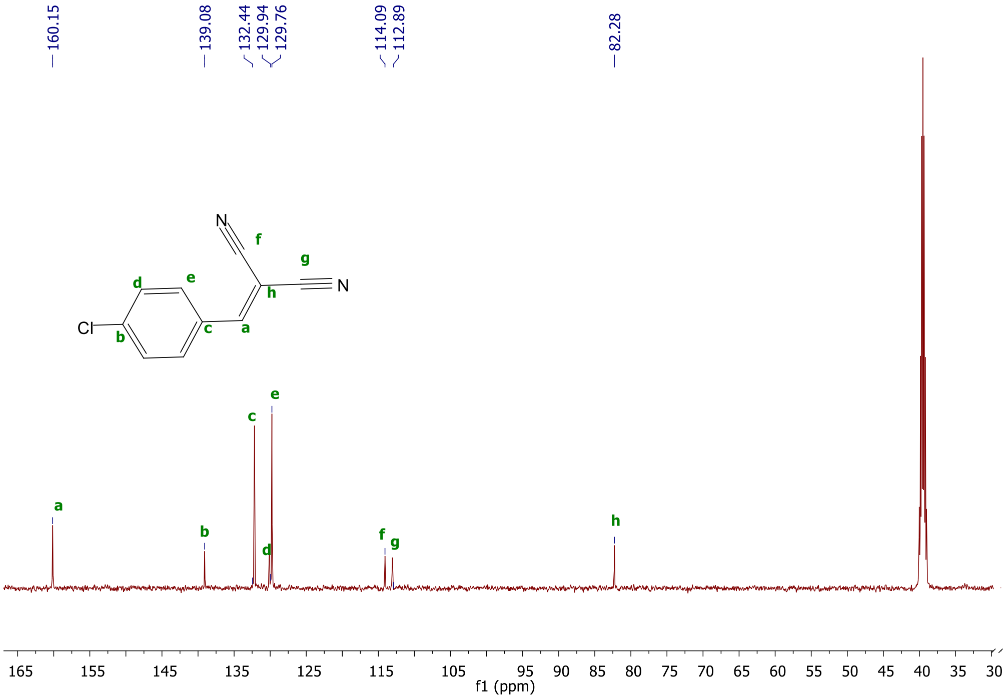

Figure S49. ^{13}C NMR of 2-(4-chlorobenzylidene) malononitrile

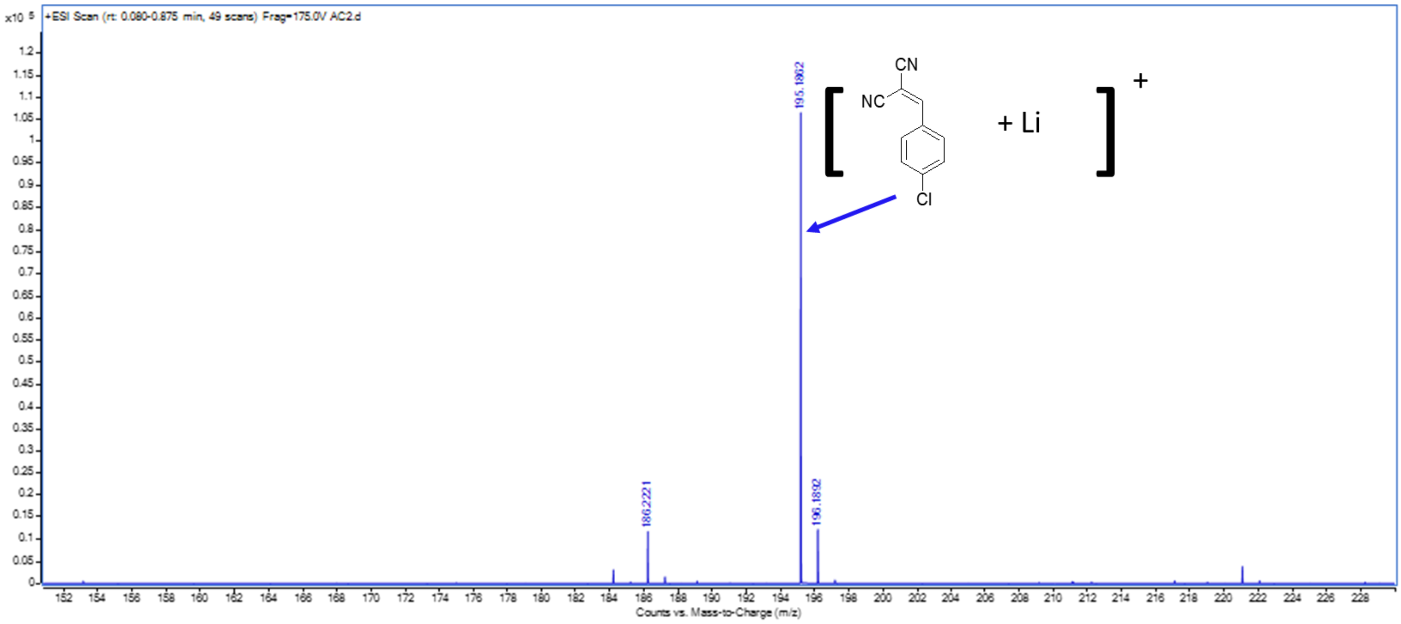

Figure S50. m/z spectra of 2-(4-chlorobenzylidene) malononitrile obtained from HRMS

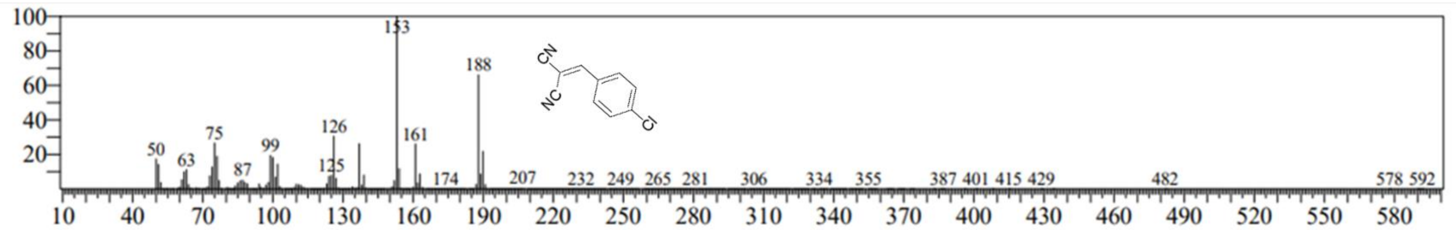

Figure S51. Mass of 2-(4-chlorobenzylidene) obtained from GCMS

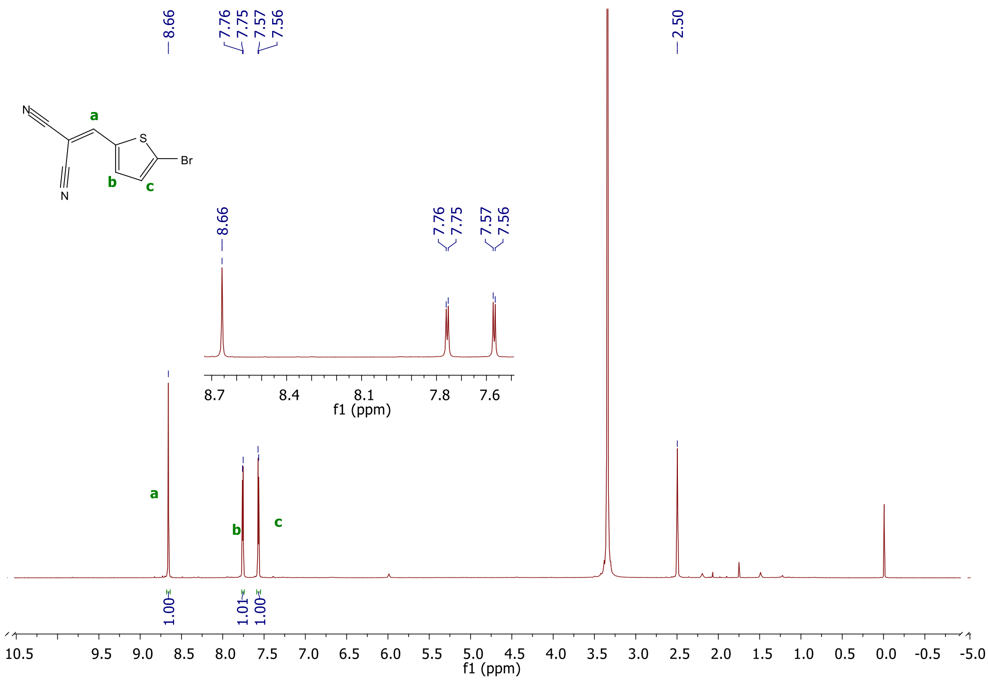

Figure S52. ^{1}H NMR of 2-((5-bromo thiophen-2-yl) methylene malononitrile

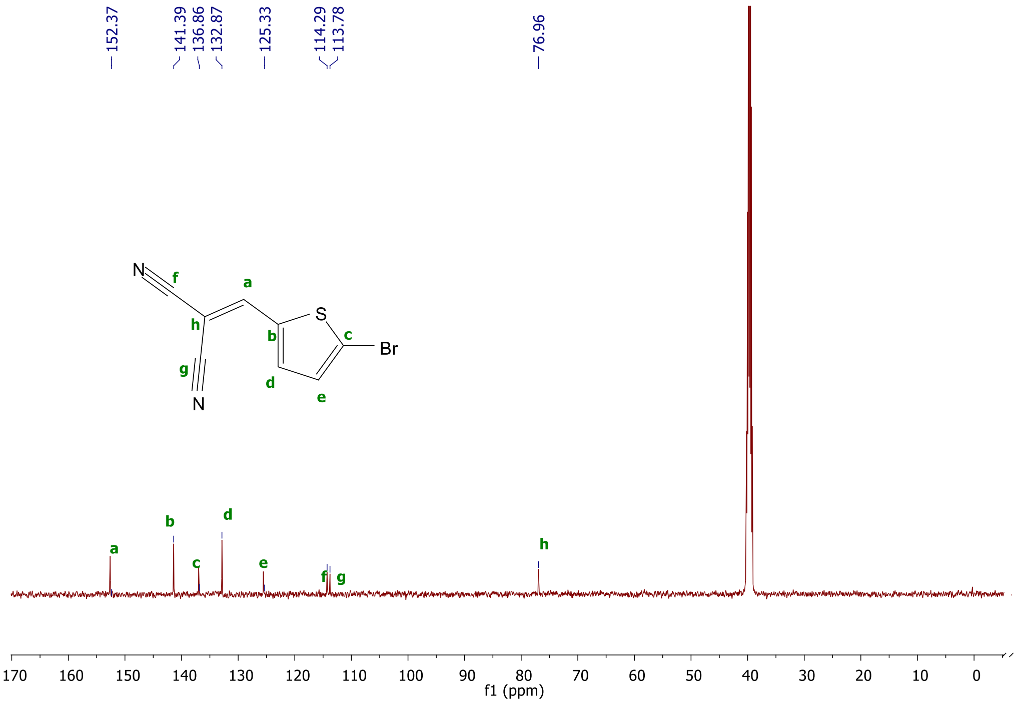

Figure S53. ^{13}C NMR of 2-((5-bromo thiophen-2-yl) methylene malononitrile

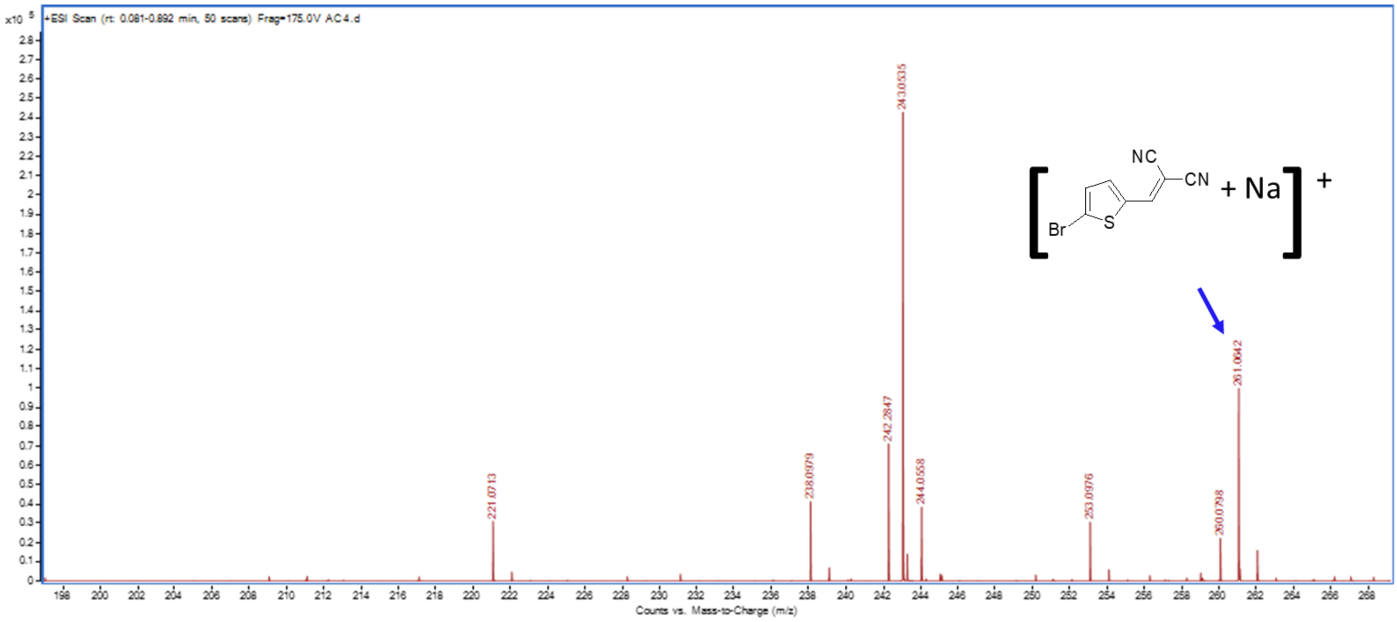

Figure S54. m/z spectra of 2-((5-bromo thiophen-2-yl) methylene malononitrile obtained from HRMS

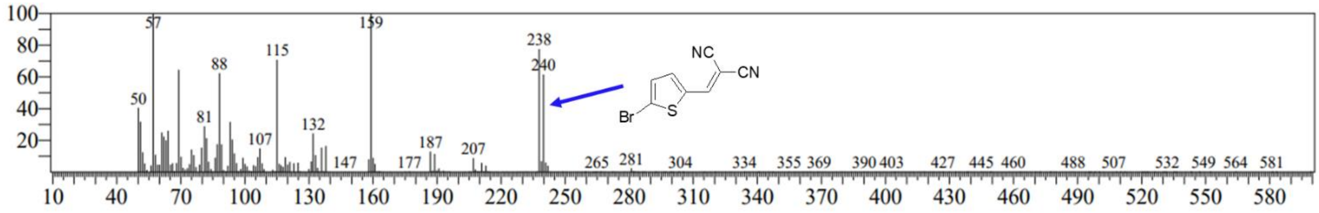

Figure S55. Mass of 2-((5-bromo thiophen-2-yl) methylene malononitrile obtained from GCMS

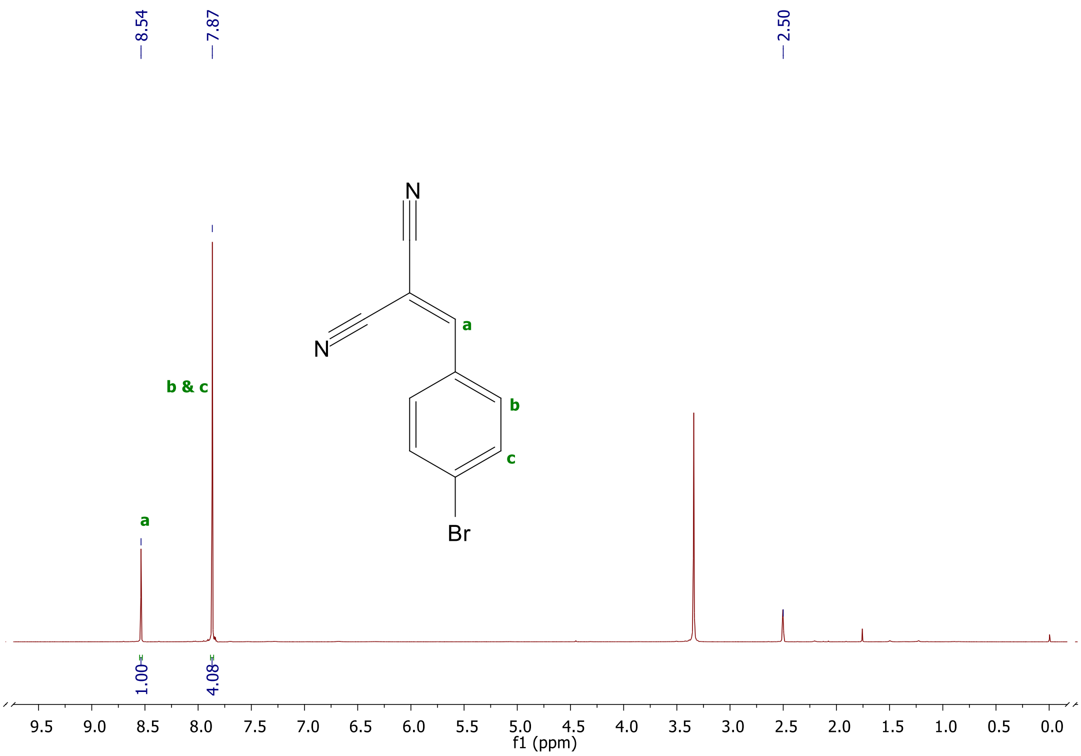

Figure S56. ^{1}H NMR of 2-(4-bromobenzylidene) malononitrile

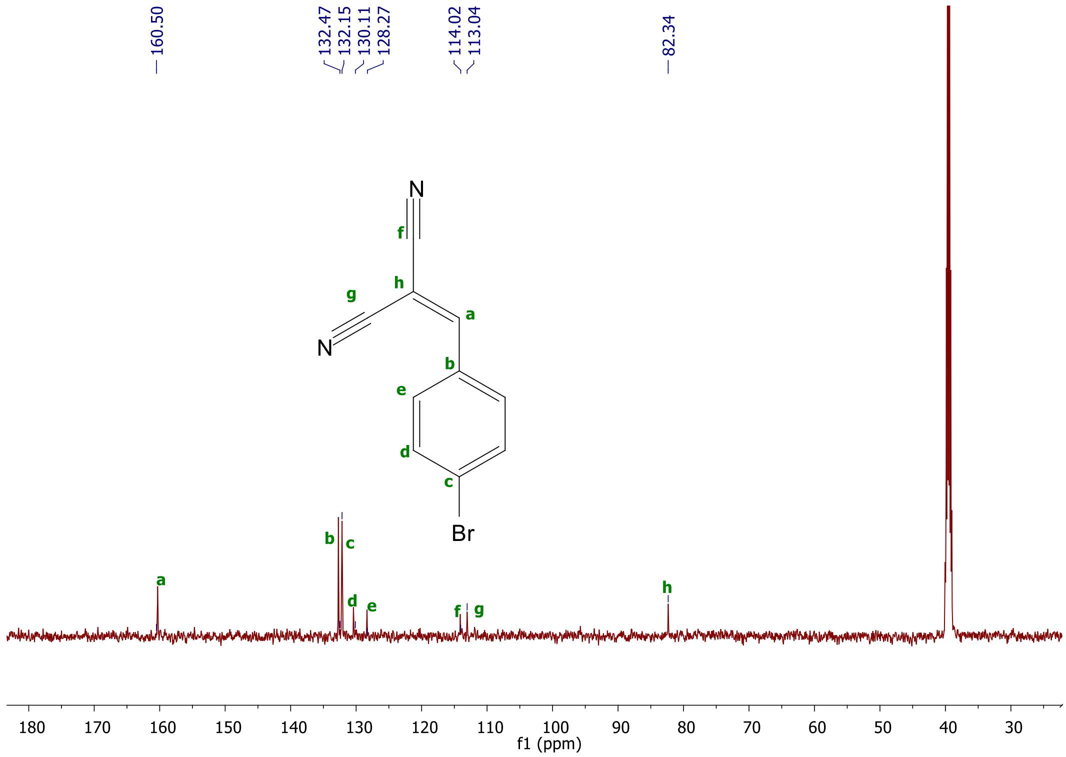

Figure S57. ^{13}C NMR of 2-(4-bromobenzylidene) malononitrile

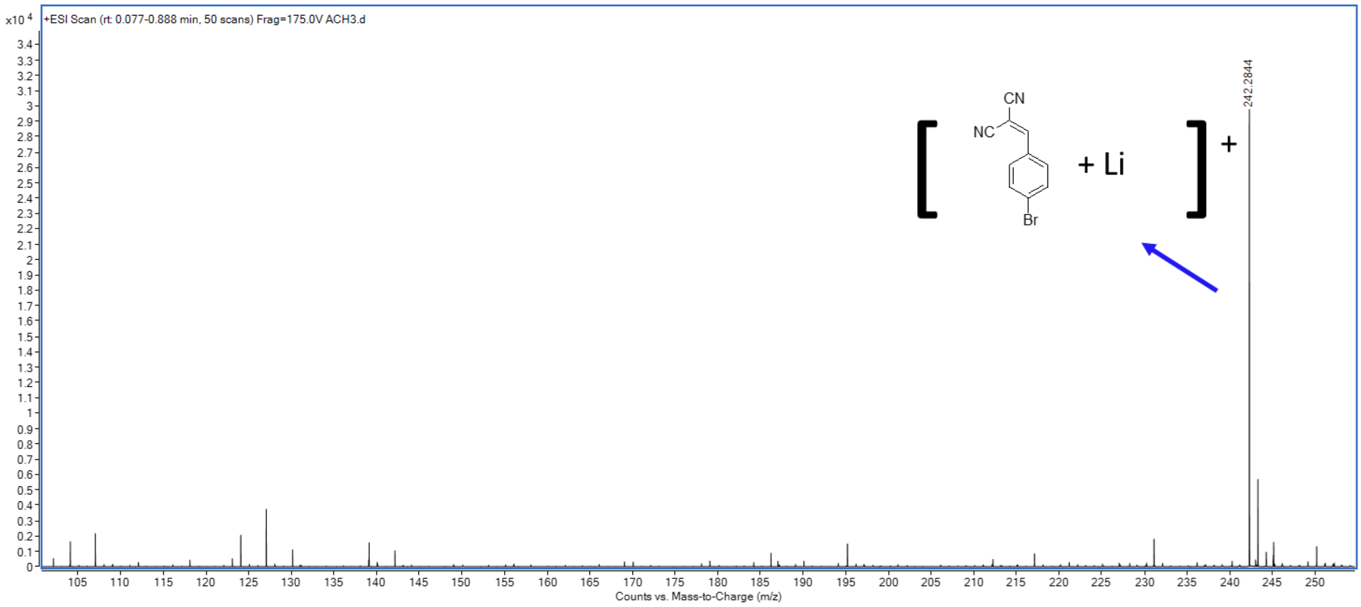

Figure S58. m/z spectra of 2-(4-bromobenzylidene) malononitrile obtained from HRMS

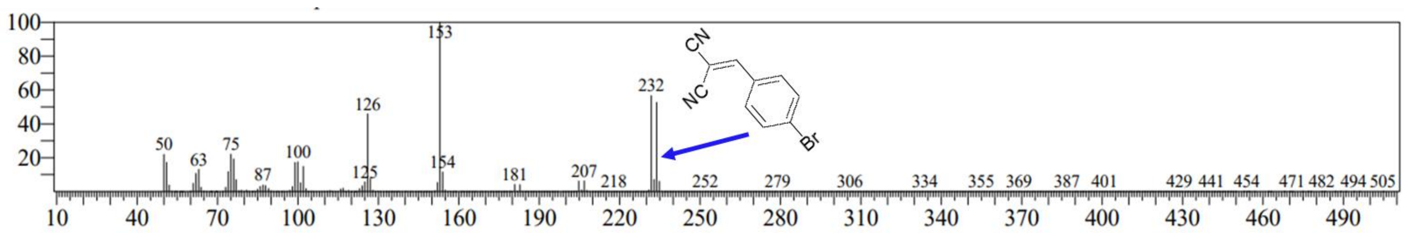

Figure S59. Mass of 2-(4-bromobenzylidene) malononitrile obtained from GCMS

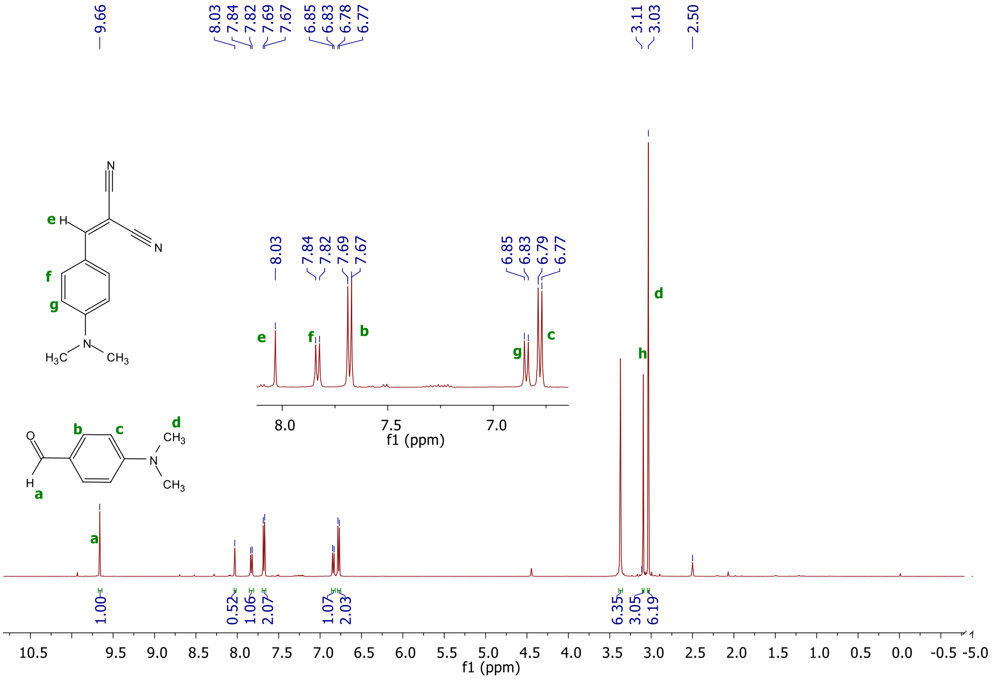

Figure S60. ^{1}H NMR of 4- (dimethyl amino) benzaldehyde

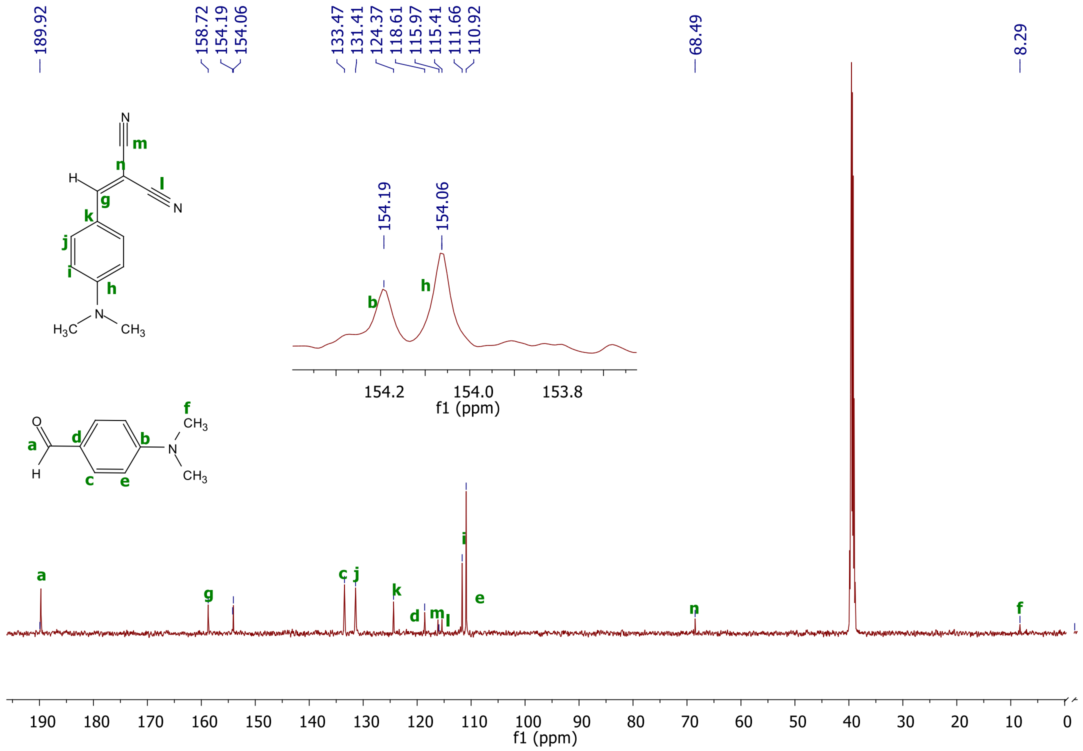

Figure S61. ^{13}C NMR of 4-(dimethyl amino) benzaldehyde.

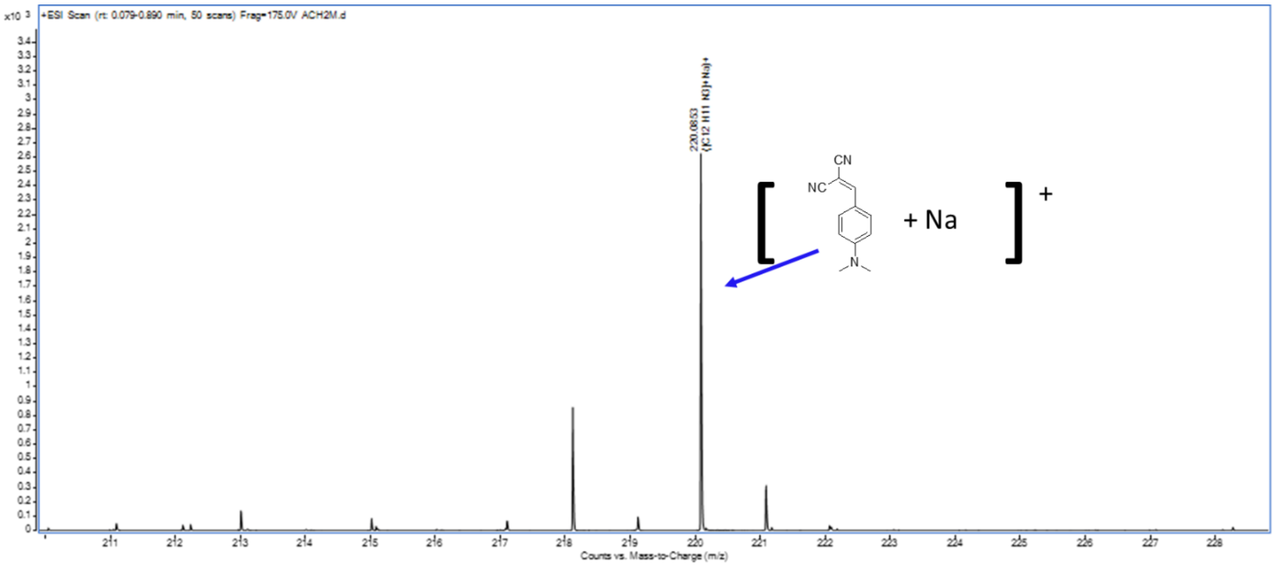

Figure S62. m/z spectra of 4- (dimethyl amino) benzaldehyde obtained from HRMS

References

[1] J. Johnson, E. Saha, A. Chhetri, E. Suresh, J. Mitra, *ACS Applied Polymer Materials* **2021**, *3*, 651-660.

[2] G. W. T. M. J. Frisch, H. B. Schlegel, G. E. Scuseria, M. A. Robb, J. R. Cheeseman, G. Scalmani, V. Barone, G. A. Petersson, H. Nakatsuji, X. Li, M. Caricato, A. Marenich, J. Bloino, B. G. Janesko, R. Gomperts, B. Mennucci, H. P. Hratchian, J. V. Ortiz, A. F. Izmaylov, J. L. Sonnenberg, D. Williams-Young, F. Ding, F. Lipparini, F. Egidi, J. Goings, B. Peng, A. Petrone, T. Henderson, D. Ranasinghe, V. G. Zakrzewski, J. Gao, N. Rega, G. Zheng, W. Liang, M. Hada, M. Ehara, K. Toyota, R. Fukuda, J. Hasegawa, M. Ishida, T. Nakajima, Y. Honda, O. Kitao, H. Nakai, T. Vreven, K. Throssell, J. A. Montgomery, Jr., J. E. Peralta, F. Ogliaro, M. Bearpark, J. J. Heyd, E. Brothers, K. N. Kudin, V. N. Staroverov, T. Keith, R. Kobayashi, J. Normand, K. Raghavachari, A. Rendell, J. C. Burant, S. S. Iyengar, J. Tomasi, M. Cossi, J. M. Millam, M. Klene, C. Adamo, R. Cammi, J. W. Ochterski, R. L. Martin, K. Morokuma, O. Farkas, J. B. Foresman, and D. J. Fox, , *Gaussian, Inc., Wallingford CT* **2016**.

[3] aA. D. Becke, *The Journal of Chemical Physics* **1993**, *98*, 5648-5652; bR. Krishnan, J. S. Binkley, R. Seeger, J. A. Pople, *The Journal of Chemical Physics* **1980**, *72*, 650-654; cS. Grimme, *J Comput Chem* **2006**, *27*, 1787-1799.
[4] J. Tomasi, B. Mennucci, R. Cammi, *Chem Rev* **2005**, *105*, 2999-3093.
[5] A. Paul, A. Karmakar, M. F. C. Guedes da Silva, A. J. Pombeiro, *Catalysts* **2021**, *11*, 90.
[6] A. Karmakar, A. Paul, G. M. Rúbio, M. M. Soliman, M. F. C. Guedes da Silva, A. J. Pombeiro, *Frontiers in Chemistry* **2019**, *7*, 699.
[7] A. Karmakar, M. M. Soliman, G. M. Rúbio, M. F. C. G. da Silva, A. J. Pombeiro, *Dalton Transactions* **2020**, *49*, 8075-8085.
[8] X.-J. Hu, Z.-X. Li, H. Xue, X. Huang, R. Cao, T.-F. Liu, *CCS Chemistry* **2020**, *2*, 616-622.
[9] I. W. Zapelini, L. L. da Silva, S. Mintova, D. Cardoso, *Microporous and Mesoporous Materials* **2023**, *362*, 112776.
[10] H. Chen, Z. Yang, H. Peng, K. Jie, P. Li, S. Ding, W. Guo, X. Suo, J. Liu, R. Yan, *Chem* **2021**, *7*, 3340-3358.
[11] N. R. Shiju, A. H. Alberts, S. Khalid, D. R. Brown, G. Rothenberg, *Angew Chem Int Ed Engl* **2011**, *50*, 9615-9619.
[12] A. A. Elmekawy, N. R. Shiju, G. Rothenberg, D. R. Brown, *Industrial & Engineering Chemistry Research* **2014**, *53*, 18722-18728.
[13] E. Gianotti, U. Diaz, A. Velty, A. Corma, *Catalysis Science & Technology* **2013**, *3*.
[14] S. Let, G. K. Dam, S. Fajal, S. K. Ghosh, *Chemical Science* **2023**, *14*, 10591-10601.
[15] F. Zhang, H. Jiang, X. Li, X. Wu, H. Li, *ACS Catalysis* **2014**, *4*, 394-401.
[16] D. Wang, B. Wang, Y. Ding, H. Wu, P. Wu, *Chemical Communications* **2016**, *52*, 12817-12820.
[17] F. Shang, J. Sun, H. Liu, C. Wang, J. Guan, Q. Kan, *Materials Research Bulletin* **2012**, *47*, 801-806.
